# Genomic islands and the evolution of livestock-associated *Staphylococcus aureus* genomes

**DOI:** 10.1042/BSR20202287

**Published:** 2020-11-25

**Authors:** Relangi Tulasi Rao, Shivani Sharma, Natesan Sivakumar, Kannan Jayakumar

**Affiliations:** 1 Department of Animal Behaviour and Physiology, School of Biological Sciences, Madurai Kamaraj University, Madurai 625021, Tamil Nadu, India; 2 Research Intern, Dr. Praveen K. Lab, Centre for Ecological Sciences, Indian Institute of Science, Bengaluru 560012, Karnataka, India; 3 Department of Molecular Microbiology, School of Biotechnology, Madurai Kamaraj University, Madurai 625021, Tamil Nadu, India

**Keywords:** genomic islands, horizontal gene transfer, Livestock-Associated Staphylococcus aureus, microbial genomics

## Abstract

**Background:** Genomic Islands (GIs) are commonly believed to be relics of horizontal transfer and associated with specific metabolic capacities, including virulence of the strain. Horizontal gene transfer (HGT) plays a vital role in the acquisition of GIs and the evolution and adaptation of bacterial genomes.

**Objective:** The present study was designed to predict the GIs and role of HGT in evolution of livestock-associated *Staphylococcus aureus* (LA-*SA*).

**Methods:** GIs were predicted with two methods namely, Ensemble algorithm for Genomic Island Detection (EGID) tool, and Seq word Sniffer script. Functional characterization of GI elements was performed with clustering of orthologs. The putative donor predictions of GIs was done with the aid of the pre_GI database.

**Results:** The present study predicted a pan of 46 GIs across the LA-*SA* genomes. Functional characterization of GI sequences revealed few unique results like the presence of metabolic operons like *leuABCD* and *folPK* genes in GIs and showed the importance of GIs in the adaptation to the host niche. The developed framework for GI donor prediction results revealed *Rickettsia* and *Mycoplasma* as the major donors of GI elements.

**Conclusions:** The role of GIs during the evolutionary race of LA-*SA* could be concluded from the present study. Niche adaptation of LA-*SA* enhanced presumably due to these GIs. Future studies could focus on the evolutionary relationships between *Rickettsia* and *Mycoplasma* sp. with *S. aureus* and also the evolution of Leucine/Isoleucine mosaic operon (*leuABCD*).

## Introduction

The gene transfer between the different bacterial species has a great impact on the evolution and transformation of bacterial pathogens. Genomic Islands (GIs) are unfamiliar gene blocks present in pathogenic and non-pathogenic prokaryotic genomes apart from the core genome [1]. They play a role in determining accessory functions, such as antibiotic resistance, secondary metabolic activities, symbiosis and other special functions related to sustaining during adverse environmental conditions. These GIs supposedly associate with the virulence of the pathogenic bacteria and are known as pathogenicity islands (PAIs) [2]. GIs have evidence of horizontal origins, i.e. Horizontal Gene Transfer (HGT), which means that an interchange of genetic information between phylogenetically distant organisms takes place [3]. The advantage of acquisition of GIs has an evolutionary edge and that is a large number of genes can be transferred and incorporated into the recipient genome. This transfer may lead to dramatic changes in an organism and ultimately result in a quantum leap in evolution [4].

During the evolution, many bacteria have equipped their genomes with DNA from other bacterial species or even genus with the help of mobile genetic elements (MGEs). These MGEs also referred to as accessory genetic elements, are therefore a potential resource for bacteria that provide adaptive strengths to improve the fitness and, potentially, pathogenicity and drug resistance [5].

For instance, in *Pseudomonas aeruginosa*, accessory gene elements have enhanced the virulence toward invertebrate *Caenorhabditis elegans* [6]. GIs’ another class of accessory gene elements contribute to the evolution and adaptation to the niche. *Staphylococcus aureus* also possesses several MGEs, including GIs but the knowledge on GIs of livestock-associated *S. aureus* (LA-*SA*) is limited.

Computational analysis of whole-genome sequences of several bacterial strains predicted that GIs are present in any given strain [7]. In the present genomic era, the number of sequenced bacterial whole genomes is increasing rapidly. Comparative genomic studies became the initial step in analyzing the microbial genomics and it helps in deciphering the evolutionary relationships [8]. Public accessibility of data of whole-genome sequences and development of bioinformatics methods makes it possible to study the evolution of bacteria, pathogenicity and other associated characteristics. Recognition and analysis of GIs contribute toward a better understanding of the evolution of the disease and the development of bacterial pathogenicity and even in understanding the evolution of host-specific strains [4,9]. Moreover, zoonotic transfer of *S. aureus* is becoming a major possible way of spreading resistant *S. aureus*. Hence, it is imperative to predict and analyze the evolution of the GIs in the livestock-associated strains, to comprehend the evolutionary developments. Pertaining to this context, the present study was planned-out in order to predict the GIs and the genetic elements’ coding by GIs in the available completed genomes of *S. aureus* which have livestock-association. Furthermore, an in-depth bioinformatics comparative analysis of predicted GIs and their functions was performed.

## Materials and methods

### Acquisition of genome sequences

Complete genome sequences of *S. aureus* strains available at NCBI’s FTP server (ftp://ftp.ncbi.nlm.nih.gov/genomes/genbank/bacteria/Staphylococcus_aureus) were retrieved only after manual verification of the Livestock association or origin. Chromosomal sequences alone were analyzed and annotated with Prokka: rapid prokaryotic genome annotation tool in local machine [10] for maintaining uniformity in the annotation files. The details of genome sequences were presented as supplementary data (Supplementary Table S1).

### Prediction of GIs

Initial comparative analysis was done with GView Server [11], to draw circular chromosomes based on the BLAST-core to check the similarity. The following existing methods were deployed to predict GIs of the genomes. These methods are Ensemble algorithm for Genomic Island Detection ((EGID)—12]), and SeqWord Sniffer-python language script [13]. EGID: a tool for improved GI detection in genomic sequences, is based on the predicted results of five existing GI programs, namely Alien Hunter [14], COLOMBO SIGI-HMM [15], INDeGenIUS [16], IslandPath [17] and PAI-DA [18]. The framework of this program includes (i) collection of prediction results from existing five programs; (ii) analysis and filtering of predicted results; and (iii) generating final consensus GI results. SeqWord Gene Island Sniffer program is based on the analysis of oligonucleotide usage variations in DNA sequences and detects putative horizontally transferred gene clusters. Chromosomal sequences alone were analyzed in the present study as GIs were integrated part of chromosomes.

### Clusters of orthologous group enrichment analysis

Clusters of Orthologous Group (COG) of proteins enrichment analysis was performed in order to establish the functional characterization of GIs. COG categorization was carried out online using the Batch version of Conserved Domain Database [19], searching against the COG database while using other default parameters. ‘NA’ was used instead when the genes did not match any COG accession numbers.

### Virulent genes analysis

To identify possible virulence factors, the curated and experimentally validated virulence factors of Virulence Factors Database (VFDB) were aligned with the ORF protein sequences. BLAST-based virulence gene analysis was performed in local machine, and virulence factor protein sequences were downloaded from VFDB (http://www.mgc.ac.cn/VFs/ [20]). A local database was built in the local machine with VFDB_SetA (curated and validated) protein sequences. Proteins encoded by GIs were aligned against this database with minimum e-value as 1e^−10^ using Blastp and were filtered with 75% identity and 95% aligned length.

### Distribution analysis of GIs

The predicted GI elements clustering analysis was performed with ClustAGE software [21] as described in the manual of the software. Heat map of Bray–Curtis (BC) [22] similarity values to the neighbor-joining tree were visualized in the online tree visualization software Interactive Tree Of Life [23].

### Credible donors of predicted GIs

A novel framework was proposed and developed to detect the GIs with the aid of the concept of island ontology and proposed island flow from the pre_GI database [24]. Initially, all available Oligonucleotide Usage Pattern (OUP) neighbors data, i.e. proposed host, compositional similarity (CS) and island distance (D) for Query Island were retrieved from the pre_GI database, except the data for the proposed island flow from the query to subject with the compositional similarity cutoff of 80%. The values of D were normalized and the data with values > 0 were removed from the analysis (*x*_i_ > 0, regarded as false positives); lower D values indicate the most probable donor. The finalized data were manually curated and the bacterial donors were removed with which there is no possible interaction, e.g. marine thermophiles and archaea have a low possibility of terrestrial bacteria to interact. Finally, all the probable donors were grouped into three categories based on the normalized values of D. Group I was regarded as potent probable neighbors, Group II had moderately probable neighbors and group III had the least probable donors (Supplementary Data S2). The credible donor relationship was established by comparing the two GIs [Query and Subject (probable donor)] with LingvoCom [24].

## Results

### Properties and comparative studies of genomes

All the points of interest of the genomes used as a part of the present study have been classified (Table 1). Among these strains, *S. aureus* ST398 has the large genome with the size of 2.87 Mb, which has bovine origin but isolated from human and the smallest genome is of *S. aureus* 71193 strain with 2.71 Mb. The genomes demonstrated a noteworthy deviation in size from one another and the GC content of all genomes appeared to be same with an estimation of 33%.

**Table 1:** Characteristic features and properties of LA-*SA* genomes selected for the present study

Strain/Feature	08BA02176	08S00974	71193	E154	ED98	ED133	ISU935	LGA251	Newbould_305	NZ15MR0322	O11	O46	RF122	ST398
Sequence type	st398	st398	st398	st398	st5	st133	st5	st130	st115	st398	st130	st130	st151	st398
Size (Mb)	2.78	2.8	2.71	2.83	2.82	2.83	2.86	2.75	2.8	2.83	2.76	2.79	2.74	2.87
Genes	2593	2613	2531	2671	2681	2710	2683	2564	2662	2750	2596	2614	2656	2738
CDS	2532	2551	2472	2609	2618	2649	2621	2501	2601	2688	2569	2562	2594	2676
tRNA genes	58	61	58	61	62	60	61	61	59	61	29	51	61	61
tmRNA genes	1	1	1	1	1	1	1	1	1	1	1	1	1	1
Repeat regions	2	0	0	0	0	0	0	0	1	0	0	0	0	0
GC%	32.94	32.94	32.93	33	32.84	32.92	32.91	32.96	32.92	32.86	32.78	32.83	32.78	32.92
Predicted GIs	14	11	13	13	12	13	13	14	13	12	11	11	14	17
Coverage in genome (%)	14.3	9.63	9.96	11.4	11.16	13.97	11.42	14.13	13.96	10.9	14.1	10.59	13.64	15.55
Coverage in proteome (%)	13.57	6.97	10.5	8.34	11.76	15.31	9.35	12.24	13.14	9.89	13.04	9.41	13.67	15.23

Abbreviation: CDS, Conserved Domain Search.

**Figure 1:**
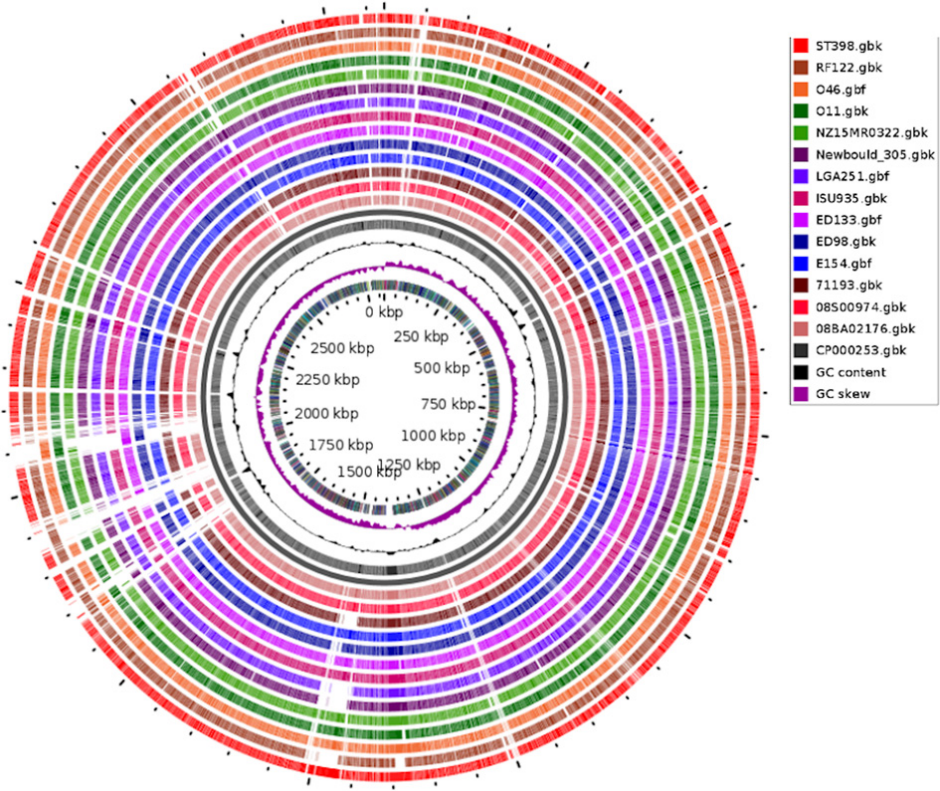
BLAST-based comparative genome maps The comparative genome map generated with the GView server using *S. aureus* NCTC8325 as a reference sequence to other *S. aureus* genomes. The outermost ring depicts the genes of a reference strain followed by the genomes of other strains based on the BLAST similarity. The genome sequences are colored differently, and regions without colors are absent from the respective strain, and highlight differences in the accessory gene content.

GView server, a BLAST-based approach, for genome comparisons was employed in order to further analyze the collinearity between genomes. It demonstrated a similarity between genomes at approximately 90–98% on sequence level and can be seen in Figure 1. Similar results of Mauve analysis were repeated here with a better understanding of the unique regions of genomes when compared with that of the reference genome. The loss and gain of genes could be seen from the whole-genome BLAST results, wherein ED133 strain had much more similar nucleotide content while RF122 strain had the least similarity. The regions around 1.8 and 2.1 Mb were unique to the majority of strains in the study. The GView server results of Blast-based comparisons were similar to the results obtained from Mauve alignment.

### Prediction of GIs

GIs were predicted with the EGID tool, and then followed by the SeqWord Gene Island Sniffer and. EGID uses the resulted GI coordinates of five existing GI prediction programs mentioned in the methods section, followed by filtering statistically significant and consensus GI coordinates [12]. SeqWord Gene Island Sniffer program analyzes the oligonucleotide usage variations in DNA sequences and detects putative horizontally transferred gene clusters [13]. The overlapping GIs from these methods was removed manually and examined the HGT mechanisms of GIs based on the annotations. Further, the coordinates of GIs were adjusted on the basis of the HGT mechanism. Additionally, the false-positive predicted GIs (e.g. ribosomal genes) and essential genes were removed from the analyses.

It was concluded that 46 different GIs prevailed across the study genomes, most GIs were 17 in number as observed in *S. aureus* ST398 strain and least were only 11 in number as found in 08S00974 strain, in spite of its larger genome-size elevated coverage by the GIs was also in accordance with the predicted number of GIs and was found to be 15.5% in whole-genome sequence and 15.3% in proteome in case of *S. aureus* ST398 (large genome) strain, whereas the least coverage was seen in *S. aureus* 08S00974 (Table 1). LA-*SA* strains possess an average of 13 GIs in their genomes and the least number of GIs was 11.

**Figure 2:**
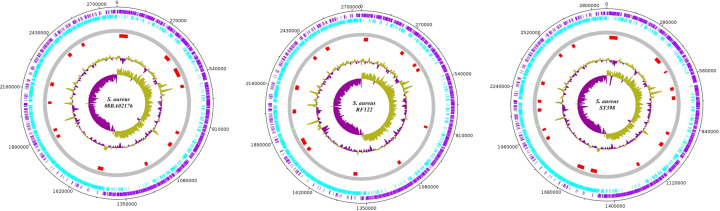
Location of GI coordinates on the circular genome The circular genome of *S. aureus* strains represent the predicted GI locations. The inner circle represents the GC skew and the next represents the GC content plot and the red dots represent the predicted GI location on the circular genome (plotted by *DNAplotter*).

Some of the known islands from other *S. aureus* strains were identified in the LA-*SA* genomes and represented in Table 2. They were regarded as PAIs since these Islands coded for virulence factors like enterotoxins and clumping factors [25]. The visualization of GIs of 08BA02176, RF122 and ST398 strains was done with *DNAplotter*, a Java-based program [26] as seen in Figure 2.

**Table 2:** Previously published Staphylococcal GIs predicted in these LA-*SA*’ genomes*

GI	LASA-GI	Coordinates	Function	08BA20716	08S00974	71193	E154	ED98	ED133	ISU935	LGA251	N305^†^	NZ15	O11	O46	RF122	ST398
φSa2	08BA02176_GI2	354723-392677	Virulence	+	+	-	+	+	+	+	-	+	+	+	+	-	+
νSaβ	LGA251_GI11	1870085-1902095	Virulence	-	-	-	-	-	+	-	+	-	-	-	-	+	-
φSa3	77193_GI10	1934043-1976257	Virulence	-	-	+	-	-	+	-	-	-	-	+	+	-	-
νSaγ	08BA02176_GI7	1182896-1202263	Virulence	+	-	+	-	+	+	-	+	+	-	-	-	+	+
SaIbov	08BA02176_GI3	458784-517982	Virulence	+	+	+	-	+	+	-	+	+	-	-	-	+	+
Type-V SSCmec	08BA02176_GI1	16107-72549	Resistance	+	+	+	-	-	+	-	+	-	-	-	-	+	+

* From PAI-DB [32].

† Newbould_305.

### Mechanisms involved in the transfer of GIs

The three common mechanisms mainly meant for HGT are through Phage integrase (Transduction), Transposon – Transposase (Insertion Sequences) and tRNA [27]. Mostly, phage-related integrase genes are present on these predicted GIs, suggesting that they are integrated and excised in a method similar to prophages*. S. aureus* Pathogenicity Island (SaPI) is mobilizable with the assistance of the transducing phages [28]. The majority of the predicted GIs (13/46) were noted to flank by transposon – transposase genes and followed by phage integrases suggesting that transposase is majorly responsible for the acquisition of GIs (Supplementary Table S2). The present study also suggests that transducing phages are responsible for the acquisition of GIs. Finally, it was concluded that from the mechanisms involved in gene transfer, transposon – transposase and phage infections are important mechanisms involved in the HGT events of the studied genomes.

### Homologous GIs

Homologs from the predicted GIs were retrieved with GET_HOMOLOGUES software [29], for understanding the relationship among LA-*SA* genomes, and also for performing comparative analysis. Further, the conserved domain superfamily analysis was also carried out for the retrieved homologs representing each strain [19]. These findings showed that only eight ORFs of all GIs are common to all strains under the study. Unique proteins of each strain are majorly noted as hypothetical proteins that belong to an unknown family of proteins. The common proteins are mainly Phage proteins and virulent proteins. The Conserved Domain Search (CDS) analysis also showed that many hypothetical proteins are assigned to the unknown family and other hypothetical proteins belong to proteins. The presence of phage proteins indicates frequent phage infections. RF122 strain’s GIs harbor the highest unique ORFs and lowest in 08S00974.

### Functional categorization and COG enrichment analysis

**Figure 3:**
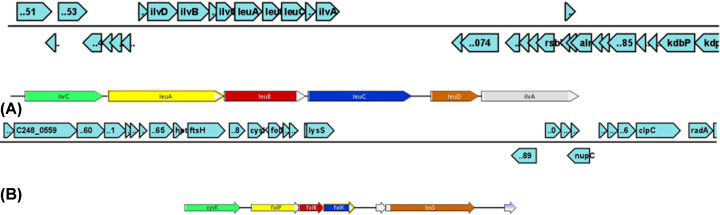
Genetic maps of the operons present in the GIs Gene maps of GIs indicating the presence of genes involved in metabolism (**A**) 08BA02176_GI11 with *leuABCD*, (**B**) 08BA02176_GI14 with folic acid biosynthesis genes.

Based on the annotation results of genomes, the predicted GIs mostly possess phage and hypothetical proteins (Supplementary Data S1). Besides the usual hypothetical proteins, several GIs encode for some virulent proteins, and also confer antibiotic resistance, as expected. Apart from these functions, some GIs encode for metabolism-related functions as well, e.g. LASA-GI4 genomes encode genes for folate synthesis and LASA-GI19 encodes for trehalose metabolism. This unique finding from the study shows the mosaic operon cluster transfer of LASA-GI11, encoding for Leucine/Isoleucine biosynthesis (Figure 3).

It was found that potent toxin genes like enterotoxins and exotoxins genes were associated with GIs and were found to have HGT origin because of their anomalous GC content (Supplementary Data S1). Phage infections were responsible for the transfer of these toxin genes. The other important genes, which have a horizontal origin, are fibronectin-binding proteins. Fibronectin-binding proteins are adhesins, cell wall-associated proteins involved in critical host–pathogen interactions [30]. These genes, found across all GIs of the study genomes and other putative fibronectin-binding proteins, are also associated with GIs. Furthermore, these proteins were reported to evolve in *S. aureus* in the ruminant host habitat [31]. When we compare the GIs including PAIs of other *S. aureus* strains (human pathogenic *S. aureus*) published in PAI-DB [32], it was observed that the GIsmajorly encoded for toxins and antibiotic resistance genes but not for fibronectin-binding proteins in major. Since all the strain genomes used in the present study have animal association, the association of fibronectin-binding proteins with GIs is significant in the view of evolutionary strategies adopted by *S. aureus*.

For approximately 1250 homologous protein sequences representing the overall GI elements of LA-*SA* genomes of the present study, only 440 sequences were assigned with COG accession numbers and categorized into different functional groups (Supplementary Data S1), since the majority of the sequences are annotated as hypothetical proteins which do not belong to the existing functional classes. Hence, the number of sequences assigned to the COG functional class was less. The overall enrichment analysis resulted in the GI elements that were with molecular functions-related elements like replication, transcription and translation regulatory proteins (25%), followed by general prediction class and proteins with unknown functions (COG class R and S – 14%), amino acid transport and metabolism-related proteins (COG class E – 9%), Phage-related elements (COG class X – 8%) and Defense-related proteins (COG class V – 5%).

### Virulent genes across the GIs

The Blastp analysis of GIs against VFDB resulted in identifying the major genes contributing the virulence in LA-*SA* (Supplementary Table S3). The enrichment analysis against a database indicated that these GIs carried important virulent genes. This analysis identified that the GIs predominantly encode for toxin genes, especially different kinds of enterotoxins followed by adherence-related genes like fibronectin and fibrinogen binding proteins, serine–aspartate rich fibrinogen-binding proteins (*sdr* genes) and clumping factor. Exotoxins and Type VII Secretion System (T7SS) genes that have been associated with virulence in *S. aureus* were also part of GIs. These results suggested that the genes associated with virulence in LA-*SA* were hustled through HGT mechanisms.

### The GI elements distribution and their relatedness

**Figure 4:**
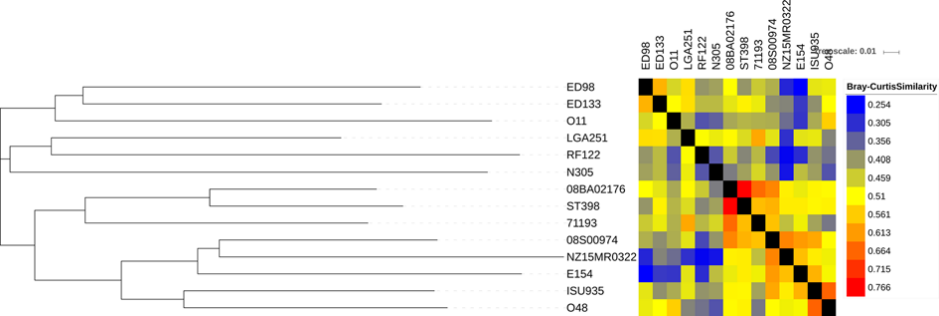
Cluster analysis of GI elements BC distances (d) calculated for every pair-wise comparison of shared GI element content between strains. Neighbor-joining tree (left) is a consensus across 1000 bootstrap resampling of distributions. The heatmap shows relative pairwise similarity (1 - d) between strains.

The homologous GI elements were distributed among the *S. aureus* genomes in the present study. The distribution and relatedness of these GI elements help to understand the implications of GIs on the strain-based relatedness, and the evolution of LA-*SA* genomes. The sequence similarity and the distribution of related GI elements among the LA-*SA* genomes were depicted in Figure 4 with BC distance-based heat map and Neighbor-joining tree. The heat map based results suggested that the GI elements among the LA-*SA* genomes have intermediate relatedness. During clustering, the GI elements resulted in BC distance ranging only from 0.25 to 0.76. This was presumably due to the differences in the composition of GI elements. The highest BC (0.766) similarity was seen between 08BA20176 and ST398, presumably, because they both belonged to st398 type. But the average BC similarity coefficient was found to be only 0.5, a moderate similarity coefficient likely due to different phage infections (frequency of certain phage groups varied between *S. aureus* Clonal Lineages – [33]), barriers such as restriction–modification systems (R–M systems) and niche separation reducing the opportunities for HGT [34]. Sequence-type based clustering was not observed when the phylogenetic tree was constructed with GI elements; and this observation suggests similar HGT events that were, presumably, not dependent on STs of strains. The highest BC similarity was seen between 08BA20176 and ST398 strains while least was found in between E154 and ED98 strains and also in NZ15MR0322 and RF122 strains. These results indicated the uniqueness of GI composition of each strain.

### Credible donors of GI elements

From the above results, it was obvious that HGT played an important role in the acquisition of GIs and the evolution of virulence and adaptation of LA-*SA* strains. Thus, it is practically significant to explore the donors of GIs in order to understand the interactions with donors, as well as to interpret the HGT events. Predictions were performed for all 14 genomes of the present study and all the results were cumulated for understanding the donors for LA-*SA* GIs better. The donor–recipient island ontology further confirmed with LingvoCom (Supplementary Data S3).

**Figure 5:**
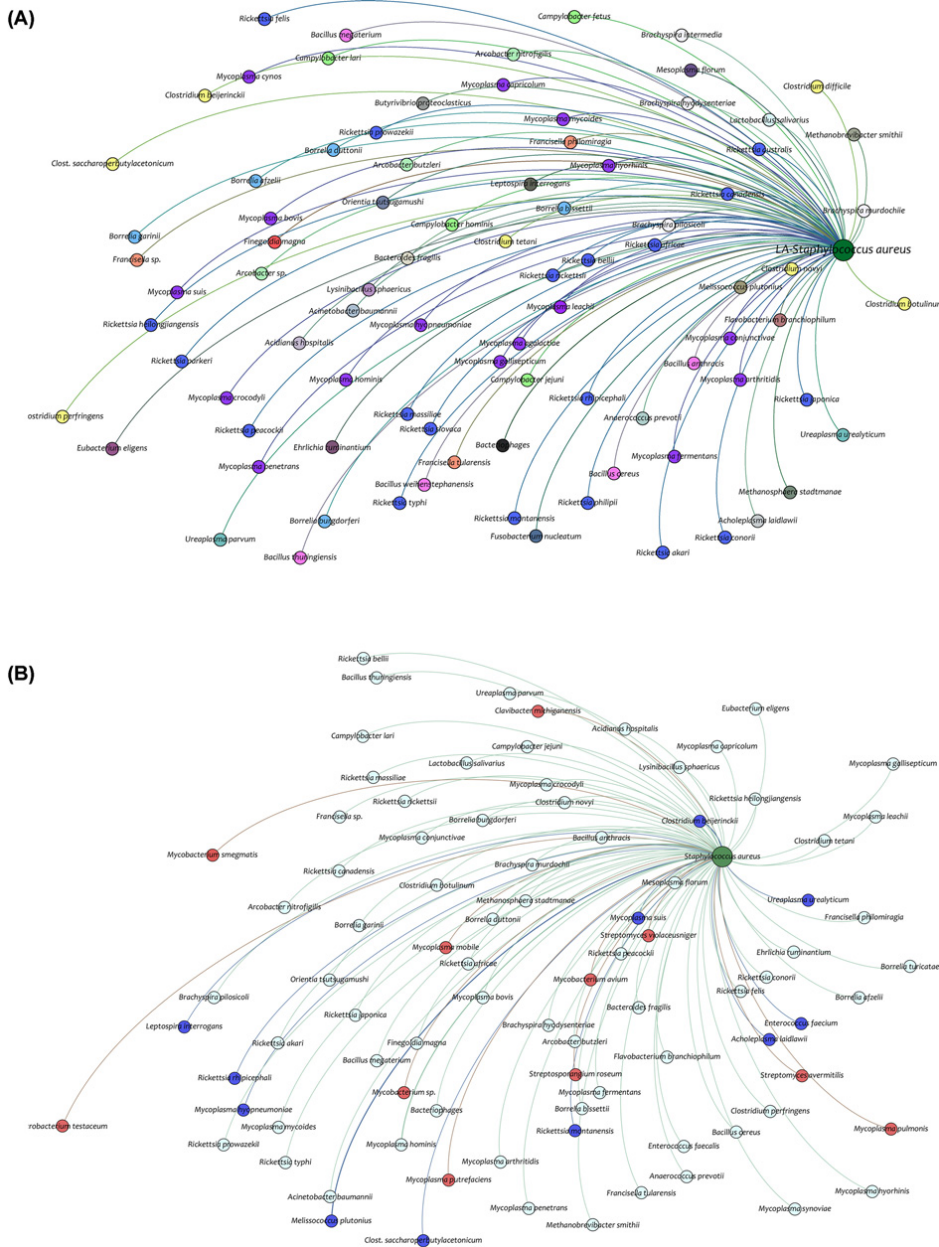
Representation of GI donors in the network model (**A**) Donors of GIs of LA-*SA* strains ad each colored node represent a different genus. (**B**) This network depicts the GI donors of both LA-*SA* strains and HA-*SA* strains. The red color nodes represent the unique donors to HA-*SA* strains while blue color for LA-*SA* strains and cyan colored nodes represents the common donors.

This framework predicted several probable donors of GIs and the data were plotted as a network with gephi tool [35]. The results showed that *Rickettsia* sp. and *Mycoplasma* sp. were over-represented as donors of GI elements (Figure 5A). The family-level representation of the donors’ list resulted in the same observation that Rickettsiaceae followed by Mycoplasmataceae were dominant donors of the GI elements of the LA-*SA* strains (Supplementary Figure S1). Further, these results compared with donors of GI elements of human-associated *S. aureus* (HA-*SA*), revealed that these strains presented with *Mycobacterium* sp. were also part of donors while some species of *Rickettsia* and *Mycoplasma* were absent from donors list of HA-*SA* (Figure 5B). These results suggested LA-*SA* and HA-*SA* strains were presented with diverse GIs, in the due course of evolution.

## Discussion

### GIs and their importance in LA-*SA* genomes

To have a better idea of the evolution of LA-*SA*, the roles of the accessory genome, and HGT events in pathogenicity, drug resistance and epidemiological information, GI prediction, and functional categorization were carried out with the available LA-*SA*’s 14 complete genomes. Several studies emphasized the importance of HGT and GIs in the evolution of *S. aureus* and identified GIs including PAIs [36,37]. A different study reported the detection of 13 known GIs including PAIs across the 5 MRSA strains [38], comparable with the present study where 13 GIs have been predicted, on average, in the 14 LA-*SA* strains. Some GIs were widely distributed across these 14 LA-*SA* genomes while some were confined to two or three strains (Supplementary Data S1). This was so perhaps due to the barriers such as DNA R–M systems and niche separation which reduce the opportunities for HGT [34].

Phages and Transposon – Transposases were found to be predominantly involved in the exchange of genetic material through HGT in LA-*SA* genomes in the present study. Moon et al. [39] in 2016 suggested that phages involved in mediating GIs which confers virulence and resistance in some *S. aureus* strains. While transposons are not able to transpose within the bacterium, but they are capable to integrate with various sites of the other host’s genome and are therefore able to transpose in the host genome [40]. Primarily, Transposon – Transposase-mediated GIs confer resistance to host strains [34]; but here we report that Transposon – Transposase-mediated GIs were predicted to encode virulence and some metabolism-related functions, apart from antibiotic resistance. A recent study by Jani et al. [38] also reported the Transposase-mediated GIs.

Functional characterization of GIs revealed that apart from the virulence and antibiotic resistance functions, certain GIs encode some metabolism-related functions. The Leucine/Isoleucine biosynthesis genes are a mosaic *leuABCD* operon and known to have horizontal transfer origin and probable source would be archaea [41]. This operon in *S. aureus* is involved in leucine and pyruvate metabolism. There were no available reports on the acquisition of *leuABCD* operon through HGT in *S. aureus*. The folic acid synthesis genes in *S. aureus* also seem to be a part of HGT events. The genes *folP, folB* and *folK*, involved in folate synthesis in *S. aureus*, were encoded by a GI in LA-*SA* genomes. This functional characterization of GIs also suggests the role of HGT in host-niche adaptation and the similar finding was well established in *Prochlorococcus* spp. and *Klebsiella pneumoniae* as well [42,43]. Another recent study on *S. aureus* also suggests the importance of GIs in the host specificity [44].

Even though many genes encoded by GIs were hypothetical proteins and phage-related protein, COG enrichment analysis revealed that certain genes encoding vital molecular functions, such as transcriptional regulatory genes, were enriched in GIs. These genes were primarily flanked with phage elements like integrase and capsid proteins, suggesting that temperate phages may responsible for this enrichment. Apart from these regulatory functions, phage proteins are known to increase the virulence of host bacterium, through regulation of expression of the virulence genes [45].

The other important genes which have horizontal origin are fibronectin-binding proteins. Fibronectin-binding proteins are adhesins, cell wall-associated proteins, involved in critical host–pathogen interactions [30]. The insights into the predicted GIs of strains clearly state that important virulence factors are associated with GIs. Sui et al. [46] reported that GIs inconsistently harbor greater number of virulence factors than the rest of the host genome, and are enriched for proteins like toxins or hypothetical pathogen-associated genes. Similar results were obtained in the present study, which located proximity to the earlier studies. Analyzing the closely related pathogenic genomes have suggested that genes involved in virulence are apparently associated with PAIs, a subset of GIs [9,46]. This again supports the notion of the role that GIs play in the transformation of non- virulent strain to virulent strain.

### GIs and their credible donors

GIs played a vital role in adaptation, survival and virulence of strains but which organisms contributed to acquiring such traits was yet to discover in the field of accessory genomics of bacteria. In the present study, an attempt was made to predict the donors of such traits, and also the relationships between host and donors. Predictions were performed for the 14 genomes of the present study and *Rickettsia* sp. and *Mycoplasma* sp. were found to have been over-represented as donors. Wan and Che [47] in 2014, also developed a similar kind of approach to predict the GI donors. They reported that *Gordonia, Nocardia* and *Rhodopseudomonas* species as major donors of GI elements of *Mycobacterium tuberculosis.* The predicted donor results also suggested the frequent phage infections and incorporated phage related genes in the genomes of LA-*SA* strains. There were few unique donors for LA-*SA* GIs and HA-*SA* GIs, presumably, because of niche separation.

There were not enough literature or reports available to support these findings of GI donors. While previous experimental research suggested that capsular polysaccharide genes of *S. aureus* were found to have homologs in the Rickettsia genome [48]. And there could be a possible membrane fusion between *Mycoplasma* and *S. aureus, Bacillus subtilis* for facilitating the transfer of conjugative elements between hosts and recipient cells [49,50]. The other plausible reasons for these results could be the interactions between *S. aureus* and other donors (or part of the microbiome of hosts) during the due course of infection. It was also reported that *Mycoplasma* and *S. aureus* are believed to coexist and cause bovine mastitis [51]. This study paves the way to realize a need to understand the evolutionary relationships with other bacterial species and in particular between *Rickettsia* and *Mycoplasma* sp. with *S. aureus*.

## Conclusions

In conclusion, the established the fact that GIs plays a vital role in niche adaptation and evolution of LA-SA strains. The predicted GIs were observed to enhance the virulence capacity of strain as they primarily encoded for adherence-related proteins, like the fibronectin-binding proteins, clumping factors and also for most of the toxin genes, like entero- and exotoxins. The GIs functional enrichment and horizontal transfer of *leuABCD* mosaic operon and folic acid biosynthesis genes (*folBPK*) in *S. aureus* suggests their role in the niche adaptation. The donor prediction results of GI elements showed that *Rickettsia* sp. and *Mycoplasma* sp., over-represented as donors of GI elements in LA-*SA* strains and Mycobacterial sp., were unique donors for the HA-*SA* associated GI elements. Based on these findings, future studies could focus on the evolutionary relationships between *Rickettsia* and *Mycoplasma* sp. with *S. aureus*, and also the evolution of Leucine/Isoleucine mosaic operon (*leuABCD*) in *S. aureus*.

## Supplementary Material

Supplementary Figure S1 and Tables S1-S3

Supplementary Data S1-S3

## Abbreviations

BC: Bray–Curtis
COG: Cluster of Orthologous Group
EGID: Ensemble algorithm for Genomic Island Detection
GI: genomic island
HA-SA: human-associated *Staphylococcus aureus*
HGT: horizontal gene transfer
LA-SA: livestock-associated *Staphylococcus aureus*
MGE: mobile genetic element
PAI: pathogenicity island
R–M system: restriction–modification system
VFDB: Virulence Factors Database
