# Supplementary material for: Genomic islands and the evolution of livestock-associated Staphylococcus aureus genomes
Source: Biosci Rep. 2020 Nov 25;40(11):BSR20202287. doi: 10.1042/BSR20202287 (PMC7689654; doi:10.1042/BSR20202287)
Supplement: Supplementary Figure S1 and Tables S1-S3 [file BSR-2020-2287_supp.pdf]

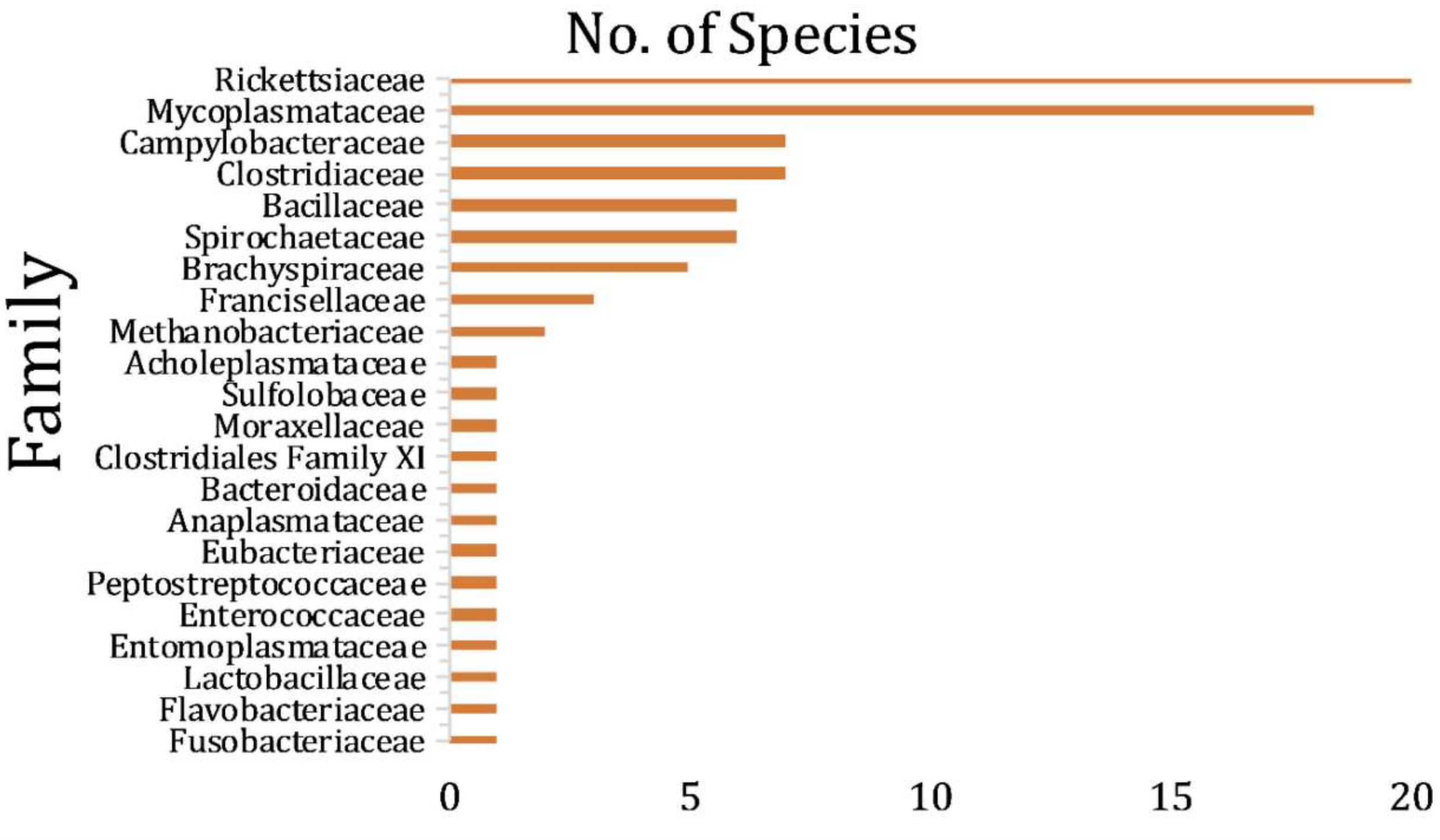

Figure S1 Categorization of GI element donors based on their family

Figure S1 Categorization of GI element donors based on their family. The predicted donors of LA-*SA* GIs' were grouped as family, and the number of species from that family was represented by the length of bars.

Table S1. General Properties of the 14 Livestock-Associated *Staphylococcus aureus* genomes retrieved from NCBI

Strain	Sequence Type	Accession Number	Sequencing Technology	Host
08BA02176	ST398	CP003808	GS FLX Titaium pyrosequencing	Cattle
08S00974	ST398	CP020019	Illumina MiSeq	Cattle
71193	ST398	CP003045	Roche genome sequencer	Cattle
E154	ST398	CP013218	--	Chicken
ED98	ST5	CP001781	454 pyrosequencing	Chicken
ED133	ST133	CP001996	454 pyrosequencing	Small Ruminants
ISU935	ST5	CP017090	--	Swine
LGA251	ST130	FR821779	454 pyrosequencing	Cattle
Newbould_305	ST115	AKYW010000	Illumina HiSeq	Cattle
NZ15MR0322	ST398	LT699704	PacBio	Cattle
O11	ST130	CP024649	Illumina Genome Analyzer GAII	Small Ruminants
O46	ST130	CP025395	Illumina Genome Analyzer GAII	Small Ruminants
RF122	ST398	AJ938182	Shotgun Sequencing	Cattle
ST398	ST398	AM990992	Pyrophospahate Sequencing	Cattle

Table S2 Distribution patterns of GIs across the fourteen LA-*SA* genomes

GI	08BA2176	08S974	71193	E154	ED98	ED133	ISU935	LGA251	Newboul	NZ1	O11	O46	RF122	ST398	GC Content	Mechanism	Major Contributions
LASA-GI1	+	-	+	-	-	+	-	+	-	-	-	-	+	+	31.6797477	Transposon	Immunity and antibiotc resistance
LASA-GI2	+	-	+	-	-	-	-	-	-	-	-	-	-	-	33.7610328	Phage	Hypothetical Proteins
LASA-GI3	+	+	+	-	+	+	-	+	+	-	-	-	+	+	32.2944644	Transposon	Toxins-Pathogenicity
LASA-GI4	+	+	-	+	-	+	+	+	-	+	-	-	-	+	39.6454862	tRNA	Folate Synthesis
LASA-GI5	+	+	+	+	+	-	+	-	-	-	-	-	-	+	30.7276935	NA	Virulence
LASA-GI6	+	-	-	+	-	-	-	-	-	+	-	-	-	+	36.167112	NA	Virulence and antibiotic resistance
LASA-GI7	+	-	+	-	+	+	-	+	+	-	-	-	+	+	30.1321768	NA	Virulence – Adherence
LASA-GI8	+	-	-	-	-	-	-	-	-	-	-	-	-	-	35.7181749	tRNA	Virulence
LASA-GI9	+	+	+	+	+	+	+	+	+	+	+	+	+	+	33.36027	tRNA	Antibiotic resistance
LASA-GI10	+	-	+	+	-	-	-	-	-	-	-	-	-	+	29.5847244	Transposon	NA
LASA-GI11	+	-	-	-	-	-	+	-	-	+	+	+	-	+	38.8052363	NA	Leucine/isoleucine biosynthesis
LASA-GI12	+	+	+	+	-	-	+	+	+	+	+	+	-	+	37.2816085	NA	Virulence – Adherence
LASA-GI13	+	+	-	-	+	-	+	+	+	+	+	+	+	+	34.3597446	NA	Virulence – Iron Aquisition
LASA-GI14	+	-	+	+	+	-	+	+	+	+	+	+	+	+	34.3101279	NA	Virulence and antibiotic resistance
LASA-GI15	-	+	+	+	-	+	-	+	-	+	-	-	-	+	31.205944	NA	Virulence – Adherence
LASA-GI16	-	+	+	+	+	+	+	+	+	-	+	+	-	+	39.9966572	NA	Virulence and antibiotic resistance
LASA-GI17	-	+	-	-	-	-	-	-	-	-	-	-	-	-	30.64652	Phage	Hypothetical Proteins
LASA-GI18	-	+	-	+	-	-	-	-	-	+	-	+	-	-	36.5712591	NA	Antibiotic resistance
LASA-GI19	-	-	+	+	+	+	+	+	-	+	+	+	+	-	37.2173272	Phage	Hypothetical Proteins
LASA-GI20	-	-	-	+	-	-	+	-	-	-	-	+	+	-	39.6322739	tRNA	Trehalose metabolism
LASA-GI21	-	-	-	-	+	-	-	-	-	-	-	-	-	-	26.8541583	NA	Antibiotic resistance
LASA-GI22	-	-	-	-	+	+	-	-	-	+	-	-	-	-	36.8922605	Transposon	Hypothetical Proteins
LASA-GI23	-	-	-	-	+	+	-	+	+	-	+	-	-	-	40.0605816	NA	Virulence – Toxins
LASA-GI24	-	-	-	-	+	-	+	-	+	-	-	-	-	-	32.0237296	Phage	Hypothetical Proteins
LASA-GI25	-	-	-	-	+	-	-	-	-	-	-	-	-	-	30.7045269	NA	NA
LASA-GI26	-	-	-	-	-	+	-	-	-	-	-	-	-	-	34.6094074	tRNA	Virulence – Toxins
LASA-GI27	-	-	-	-	-	+	-	-	-	-	-	-	-	-	29.8438981	Phage	Hypothetical Proteins
LASA-GI28	-	-	-	-	-	+	-	-	-	-	-	-	-	-	35.1715317	Phage	Toxins-Pathogenicity
LASA-GI29	-	-	-	-	-	-	+	-	-	-	-	-	-	-	29.8652591	NA	Toxins-Pathogenicity
LASA-GI30	-	-	-	-	-	-	+	+	-	-	-	-	-	-	35.3210565	Transposon	Virulence and antibiotic resistance
LASA-GI31	-	-	-	-	-	-	-	+	-	-	-	-	-	-	37.1447325	NA	NA
LASA-GI32	-	-	-	-	-	-	-	-	+	-	-	-	-	-	40.4467135	Transposon	NA
LASA-GI33	-	-	-	-	-	-	-	-	+	-	-	-	-	-	31.2197022	NA	Hypothetical Proteins
LASA-GI34	-	-	-	-	-	-	-	-	+	-	-	-	-	-	34.989423	NA	NA
LASA-GI35	-	-	-	-	-	-	-	-	+	-	-	-	-	-	35.1886396	Transposon	Virulence
LASA-GI36	-	-	-	-	-	-	-	-	-	+	-	+	-	+	30.722953	Phage	Hypothetical Proteins
LASA-GI37	-	-	-	-	-	-	-	-	-	-	+	-	-	-	36.8259804	Transposon	Toxins-Pathogenicity
LASA-GI38	-	-	-	-	-	-	-	-	-	-	+	-	-	+	30.2064079	NA	Metabolism related
LASA-GI39	-	-	-	-	-	-	-	-	-	-	-	+	-	-	35.419869	Transposon	Metabolism related
LASA-GI40	-	-	-	-	-	-	-	-	-	-	-	-	+	-	35.8041138	NA	Virulence
LASA-GI41	-	-	-	-	-	-	-	-	-	-	-	-	+	-	31.1702718	NA	Hypothetical Proteins
LASA-GI42	-	-	-	-	-	-	-	-	-	-	-	-	+	-	29.6798588	Transposon	Virulence
LASA-GI43	-	-	-	-	-	-	-	-	-	-	-	-	+	-	30.295941	NA	Virulence – Adherence
LASA-GI44	-	-	-	-	-	-	-	-	-	-	-	-	+	-	35.8607324	Transposon	Metabolism related
LASA-GI45	-	-	-	-	-	-	-	-	-	-	-	-	-	+	40.7430506	Transposon	Hypothetical Proteins
LASA-GI46	-	-	-	-	-	-	-	-	-	-	-	-	-	+	37.5417913	Phage	Hypothetical Proteins

Table S3 Virulent genes associated with predicted Gis of LA*SA*' genomes

**Protein ID**	**VFDB Accession No.**	**Related genes**	**Virulence Factors**	**VF Class**
ACY11319.1; ACY11320.1; ACY11321.1; AFR3846.1; ATV04656.1; CAI80978.1; CAI80979.1; CAI80980.1; CAI80981.1; CAI80982.1; CAI80989.1; SaureusN305_02140	VFG004506(gi:15927015)	*ebh*	Cell wall associated fibronectin binding protein	Adherence
ACY12392.1; AFR73793.1; ASC52031.1; SaureusN305_00371; SaureusN305_02431	VFG043588(gi:57651004)	*sasG*	cell wall surface anchor family protein	
AFR72873.1; ASC50323.1; CAI80432.1; SaureusN305_01293; YP_005733664.1	VFG004638(gi:87160156)	*clfA*	Clumping factor A	
AFR73178.1	VFG004496(gi:49483319)	*efb*	Fibrinogen binding protein	
AFR74495.1	VFG001283(gb|NP_647238)	*fnbA*	Fibronectin binding proteins	
AFR74492.1; AFR74494.1; ATV04655.1; SaureusNZMR0322_02541; SaureusNZMR0322_02542	VFG004615(gi:15928081)	*fnbB*		
Saureus08S00974_00548	VFG004606(gi:49482791)	*sdrC*	Ser-Asp rich fibrinogen-binding proteins	
Saureus08S00974_00549	VFG017111(gi:148267022)	*sdrD*		
Saureus08S00974_00550	VFG017226(gi:151220737)	*sdrE*		
AFR72875.1	VFG002418(gb|YP_001331791)	*vwbp*	secreted von Willebrand factor-binding protein precursor	
AFR72876.1	VFG043451(gi:57650133)	*emp*	secretory extracellular matrix and plasma binding protein	
AFR72040.1	VFG002420(gb|NP_644838)	*adsA*	Adenosine synthase A	Enzyme
AFR73846.1	VFG004744(gi:15927782)	*hysA*	Hyaluronate lyase	
ACY11709.1	VFG004703(gi:15927387)	*splA*	Serine Protease	
ADI98301.1; ATV02856.1	VFG004694(gi:82751395)	*splB*		
ADI98300.1; SaureusN305_00083; SaureusN305_00084	VFG004685(gi:82751394)	*splC*		
ACY11706.1	VFG004678(gi:87160514)	*splD*		
CAI81359.1; CCC88458.1	VFG004674(gi:82751393)	*splE*		
ADI98299.1	VFG004665(gi:82751392)	*splF*		
AFR72878.1	VFG004737(gi:49483047)	*nuc*	thermonuclease precursor	
AFH70185.1	VFG002422(gb|YP_001332911)	*chb*	chemotaxis-inhibiting protein	Immune evasion
CAI79911.1	VFG002405(gb|NP_645073)	*esxA*	Type VII Secretion System	Secretion System
CAI79912.1	VFG018114(gi:82749989)	*esaA*		
CAI79914.1	VFG002407(gb|NP_645076)	*esaB*		
CAI79915.1	VFG002408(gb|NP_645078)	*essB*		
CAI79916.1	VFG018165(gi:82749992)	*essC*		
CAI79917.1	VFG018167(gi:156978617)	*esaC*		
CAI79918.1	VFG018189(gi:82749994)	*esxB*		
AFR73182.1	VFG001293(gb|NP_645861)	*hly*	Alpha hemolysin	Toxin
AFH70247.1	VFG001798(gb|YP_186826)	*hlb*	beta-hemolysin	
ATV03271.1; ATV04671.1	VFG043452(gi:57651346)	*hld*	Delta hemolysin	
ADI96966.1	VFG005006(gi:82750124)	*sec*	Enterotoxin C-bovine	
ACY11722.1; CAI81385.1	VFG005003(gi:15927398)	*seg*	Enterotoxin G	
ADI96967.1	VFG004988(gi:82750125)	*sell*	Enterotoxin-like L	
ACY11727.1	VFG004985(gi:15927403)	*selm*	Enterotoxin-like M	
ACY11723.1	VFG004982(gi:15927399)	*seln*	Enterotoxin-like N	
ACY11728.1	VFG004978(gi:15927404)	*selo*	Enterotoxin-like O	
ADI97633.1	VFG005013(gi:15924938)	*selp*	Enterotoxin-like P	
CAI81387.1	VFG004970(gi:82751420)	*selU*	Enterotoxin-like U	
ACY11724.1	VFG004966(gi:15927400)	*yent2*	Enterotoxin Yent2	
ACY11725.1	VFG004968(gi:15927401)	*yent1*	Enterotoxin Yent1	
AFR73191.1	VFG004877(gi:82750780)	*eta*	Exfoliative toxin type A	
ACY10313.1	VFG004942(gi:15926105)	*set12*	exotoxin	
AFR72485.1	VFG004959(gi:15926099)	*set6*		
AFR72486.1	VFG004956(gi:15926100)	*set7*		
AFR72487.1; AFR72488.1	VFG004964(gi:49482653)	*set2*		
AFR72490.1	VFG004963(gi:49482655)	*set10*		
AFR72492.1	VFG004965(gi:49482656)	*set11*		
AFR72493.1	VFG004961(gi:49482657)	*set13*		
AFR72494.1	VFG004962(gi:49482658)	*set14*		
AFR72497.1	VFG004901(gi:82750145)	*set15*		
ADI98315.1	VFG004862(gi:82751408)	*lukD*	Leukotoxin D	
ADI98372.1	VFG004852(gi:82750532)	*lukE*	Leukotoxin E	
ADI98503.1; SaureusN305_02303	VFG044014(gi:87162038)	*lukH*	leukocidin family protein	
