# Supplementary material for: Genomic islands and the evolution of livestock-associated Staphylococcus aureus genomes
Source: Biosci Rep. 2020 Nov 25;40(11):BSR20202287. doi: 10.1042/BSR20202287 (PMC7689654; doi:10.1042/BSR20202287)
Supplement: Supplementary Data S1-S3 [file BSR-2020-2287_supp1.pdf]

Protein ID	08BA2176	08S974	71193	E154	ED98	ED133	ISU935	LGA251	Newbould_305	NZ15M	O11	O46	RF122	ST398	COG Accessio	COG Class	Function
APE88908.1	-	-	-	+	-	-	+	+	-	-	-	+	+	-	NA	NA	hypothetical protein AS852_09550
APE88909.1	-	-	-	+	-	-	+	-	-	-	-	-	-	-	NA	NA	hypothetical protein AS852_09555
APE88911.1	-	-	-	+	-	+	-	-	+	-	-	-	-	+	NA	NA	hypothetical protein AS852_09565
APE88913.1	-	-	-	+	-	-	+	-	-	-	-	+	+	+	NA	NA	hypothetical protein AS852_09575
APE88917.1	-	-	-	+	-	+	+	+	-	+	+	-	+	-	NA	NA	hypothetical protein AS852_09595
APE88918.1	-	-	-	+	-	+	-	+	-	-	-	-	+	-	NA	NA	hypothetical protein AS852_09600
APE88919.1	-	-	-	+	-	+	-	+	-	-	-	-	+	-	COG0305	L	damage-inducible protein
APE88920.1	-	-	-	+	-	+	-	+	-	-	-	+	+	-	NA	NA	hypothetical protein AS852_09610
APE88921.1	-	-	-	+	-	+	-	+	-	-	-	-	+	-	NA	NA	DnaD domain protein
APE88922.1	-	-	-	+	-	+	+	+	+	+	+	-	+	-	NA	NA	hypothetical protein AS852_09620
APE88963.1	-	-	-	+	-	-	-	-	-	+	-	+	-	-	NA	NA	hypothetical protein AS852_09870
APE88964.1	-	-	-	+	-	-	-	-	-	+	-	+	-	-	NA	NA	glucosamine-6-phosphate isomerase
AFR72383.1	+	-	+	-	+	+	-	-	-	-	-	+	-	+	NA	NA	Phage Holin
APE89083.1	-	-	-	+	-	-	+	-	-	+	+	+	-	+	COG0028	EH	acetolactate synthase, large subunit, biosynthetic type
APE89084.1	-	-	-	+	-	-	+	-	-	+	-	-	-	+	COG0440	E	acetolactate synthase 1 regulatory subunit
AFR72384.1	+	-	+	-	+	+	-	-	-	-	-	+	-	+	NA	NA	amidase
APE89093.1	-	-	-	+	-	-	-	-	-	+	+	+	-	-	COG1191	K	RNA polymerase sigma factor SigB
APE89094.1	-	-	-	+	-	-	-	-	-	+	+	+	-	-	COG2172	T	anti-sigma B factor RsbW
APE89095.1	-	-	-	+	-	-	-	-	-	+	+	+	-	-	COG1366	T	anti-anti-sigma factor
APE89096.1	-	-	-	+	-	-	-	-	-	+	+	+	-	-	COG2208	TK	serine/threonine protein phosphatase
APE89097.1	-	-	-	+	-	-	-	-	-	-	-	-	-	-	NA	NA	hypothetical protein AS852_10755
APE89098.1	-	-	-	+	-	-	-	-	-	-	-	-	-	-	COG2337	V	PemK family transcriptional regulator
APE89099.1	-	-	-	+	-	-	-	-	-	+	+	+	-	-	NA	NA	antitoxin MazE
APE89100.1	-	-	-	+	-	-	-	-	-	+	+	+	-	-	COG0787	M	alanine racemase
APE89101.1	-	-	-	+	-	-	-	-	-	+	+	+	-	-	COG0736	I	holo-ACP synthase
AFR72460.1	+	-	+	-	+	+	-	+	-	-	-	-	+	+	COG0518	F	putative GMP synthase
APE89102.1	-	-	-	+	-	-	-	-	-	+	+	+	-	-	COG3402	S	hypothetical protein AS852_10780
APE89103.1	-	-	-	+	-	-	-	-	-	+	+	+	-	-	COG3428	S	hypothetical protein AS852_10785
APE89104.1	-	-	-	+	-	-	-	-	-	-	-	-	-	-	NA	NA	hypothetical protein AS852_10790
AFR72461.1	+	-	+	-	-	+	-	+	+	-	-	-	+	+	COG0582	LX	Integrase
AFR72462.1	+	-	-	-	-	+	-	-	-	-	-	-	+	+	NA	NA	Transcriptional regulator
AFR72463.1	+	-	-	-	-	+	-	-	-	-	-	-	+	+	NA	NA	phage protein
AFR72464.1	+	-	-	-	-	+	-	-	+	-	-	-	+	+	NA	NA	pathogenicity island protein
AFR72465.1	+	-	-	-	+	+	-	+	+	-	-	-	+	+	NA	NA	hypothetical protein C248_0452
AFR72037.1	+	-	+	-	-	+	-	+	-	-	+	-	+	+	NA	NA	putative exported protein
APE89536.1	-	-	-	+	-	-	-	-	-	+	-	-	-	-	NA	NA	restriction endonuclease
AFR72466.1	+	-	-	-	+	+	-	+	+	-	-	-	+	+	NA	NA	hypothetical protein C248_0453
AFR72467.1	+	-	-	-	+	+	-	+	+	-	-	-	+	+	NA	NA	Hypothetical protein C248_0454
ADI96568.1	-	-	-	-	-	+	-	-	-	-	-	-	-	-	NA	NA	conserved hypothetical protein
ADI96569.1	-	-	-	-	-	+	-	-	-	-	-	-	-	-	NA	NA	putative helicase
AFR72468.1	+	-	-	-	-	+	-	-	+	-	-	-	-	+	NA	NA	putative primase
ADI96570.1	-	-	-	-	-	+	-	-	-	-	-	-	-	-	NA	NA	hypothetical protein SAOV_0028
ADI96571.1	-	-	-	-	-	+	-	-	-	-	-	-	-	-	NA	NA	hypothetical protein SAOV_0029
ADI96572.1	-	-	-	-	-	+	-	-	-	-	-	-	-	-	COG2963	X	putative insertion element protein
ADI96573.1	-	-	-	-	-	+	-	-	-	-	-	-	-	-	COG2801	X	putative insertion element protein
ADI96575.1	-	-	-	-	-	+	-	-	-	-	-	-	-	-	NA	NA	putative transposase, truncation
ADI96576.1	-	-	-	-	-	+	-	-	-	-	-	-	+	-	COG0042	J	hypothetical protein SAOV_0034
ADI96577.1	-	-	-	-	-	+	-	-	-	-	-	-	-	-	NA	NA	hypothetical protein SAOV_0035
ADI96578.1	-	-	-	-	+	+	-	+	+	-	-	-	+	+	COG0730	S	hypothetical protein SAOV_0036
ADI96579.1	-	-	-	-	+	+	-	+	+	-	-	-	+	+	COG1937	K	conserved hypothetical protein
AFR72469.1	+	-	-	-	+	+	-	-	+	-	-	-	+	+	NA	NA	hypothetical protein C248_0456

ADI96580.1	-	-	-	-	+	+	-	+	+	-	-	-	+	+	COG0425	O	conserved hypothetical protein
ADI96581.1	-	-	-	-	+	+	-	+	+	-	-	-	+	-	COG0607	P	metallo-beta-lactamase family protein
ADI96582.1	-	-	-	-	+	+	-	+	+	-	-	-	+	-	COG0446	I	conserved hypothetical protein
ADI96583.1	-	-	-	-	+	+	-	+	+	-	+	+	-	-	COG0042	J	hypothetical protein SAOV_0041
ADI96584.1	-	-	-	-	+	+	-	-	-	-	+	-	-	-	NA	NA	hypothetical protein SAOV_0042
ADI96585.1	-	-	-	-	+	+	-	+	+	-	+	+	-	-	COG3070	K	conserved hypothetical protein
ADI96586.1	-	-	-	-	+	+	-	-	+	-	+	+	-	-	NA	NA	hypothetical protein SAOV_0044
ADI96587.1	-	-	-	-	+	+	-	-	+	-	-	-	-	-	COG1112	L	hypothetical protein SAOV_0045
ADI96588.1	-	-	-	-	+	+	-	+	+	-	+	+	-	-	NA	NA	hypothetical protein SAOV_0046
AFR72470.1	+	-	-	-	-	+	-	-	+	-	-	-	-	+	NA	NA	hypothetical protein C248_0457
ADI96591.1	-	-	-	-	+	+	-	+	+	-	-	-	-	-	NA	NA	1-phosphatidylinositol phosphodiesterase
ADI96592.1	-	-	-	-	-	+	-	+	-	-	-	-	-	-	COG2207	K	putative transcriptional regulator
ADI96593.1	-	-	-	-	-	+	-	+	-	-	-	-	-	-	COG1473	R	putative peptidase
ADI96811.1	-	-	-	-	+	+	-	+	-	-	-	+	-	-	NA	NA	putative prophage integrase
ADI96812.1	-	-	-	-	-	+	-	-	-	-	-	+	-	-	COG1266	O	CAAX amino terminal protease family
ADI96813.1	-	-	-	-	-	+	-	-	-	-	-	-	-	-	NA	NA	hypothetical protein SAOV_0276
ADI96814.1	-	-	-	-	-	+	-	-	-	-	-	+	-	-	NA	NA	putative lipoprotein
ADI96815.1	-	-	-	-	-	+	-	-	-	-	+	+	-	+	NA	NA	hypothetical phage-related protein
ADI96816.1	-	-	-	-	-	+	-	-	-	-	+	+	-	-	COG1396	K	phage repressor
AFR72471.1	+	-	-	-	-	+	-	-	+	-	-	-	-	+	NA	NA	hypothetical protein C248_0458
ADI96817.1	-	-	-	-	-	+	-	-	-	-	+	+	-	-	NA	NA	hypothetical phage-related protein
ADI96818.1	-	-	-	-	-	+	-	-	-	-	-	-	-	-	NA	NA	hypothetical protein SAOV_0281
ADI96819.1	-	-	-	-	-	+	-	-	-	-	+	+	-	-	NA	NA	hypothetical protein SAOV_0282
ADI96824.1	-	-	-	-	-	+	-	-	-	-	-	-	-	-	NA	NA	hypothetical protein SAOV_0287
ADI96825.1	-	-	-	-	-	+	-	-	-	+	-	-	+	-	NA	NA	hypothetical protein SAOV_0288
ADI96826.1	-	-	-	-	-	+	-	-	-	-	-	-	+	-	NA	NA	putative topisomerase
AFR72472.1	+	-	-	-	+	+	-	+	+	-	-	-	+	+	NA	NA	hypothetical protein C248_0459
ADI96827.1	-	-	-	-	-	+	-	-	-	-	-	-	-	-	COG0629	L	Single-stranded DNA-binding protein, phage associated
ADI96829.1	-	-	-	-	-	+	-	-	-	-	-	-	-	-	NA	NA	Phage replication initiation
ADI96832.1	-	-	-	-	-	+	-	-	-	-	-	-	-	-	NA	NA	hypothetical phage-related protein
ADI96836.1	-	-	-	-	+	-	-	-	-	-	+	-	-	-	NA	NA	Transcriptional regulator, Cro/CI family
AFR72473.1	+	-	-	-	-	-	-	-	-	-	-	-	-	-	NA	NA	adenosine deaminase
ADI96841.1	-	-	-	-	-	+	-	-	-	-	+	+	-	-	NA	NA	hypothetical protein SAOV_0304
ADI96844.1	-	-	-	-	-	+	-	-	-	-	-	+	-	-	NA	NA	hypothetical protein SAOV_0307
ADI96845.1	-	-	-	-	-	+	-	-	-	-	-	-	-	-	NA	NA	hypothetical protein SAOV_0308
AFR72474.1	+	-	+	-	-	+	-	-	+	-	-	-	-	-	NA	NA	Hypothetical protein C248_0461
AFR72038.1	+	-	+	-	-	+	-	+	-	-	+	-	+	+	NA	NA	putative exported protein
AFR72476.1	+	-	-	-	-	-	-	-	-	-	-	-	-	-	NA	NA	hypothetical protein C248_0463
ADI96867.1	-	-	-	-	-	+	-	-	-	-	-	-	-	-	NA	NA	Phage tail length tape-measure protein
ADI96868.1	-	-	-	-	-	+	-	-	-	-	-	-	-	-	NA	NA	Phage tail length tape-measure protein
AFR72477.1	+	-	-	-	-	-	-	-	-	-	-	-	-	-	NA	NA	hypothetical protein C248_0464
ADI96877.1	-	-	-	-	-	+	-	-	-	-	-	-	-	-	NA	NA	hypothetical protein SAOV_0340
ADI96880.1	-	-	-	-	-	+	-	-	-	-	-	-	-	-	NA	NA	hypothetical protein SAOV_0343
ADI96881.1	-	-	-	-	-	+	-	-	+	-	-	+	+	-	NA	NA	hypothetical protein SAOV_0344
ADI96882.1	-	-	-	-	-	+	-	-	-	-	-	-	-	-	NA	NA	hypothetical protein SAOV_0345
ADI96883.1	-	-	-	-	-	+	-	-	-	-	-	-	-	-	COG0657	I	hypothetical protein SAOV_0346c
ADI96884.1	-	-	-	-	-	+	-	-	-	-	-	-	-	-	COG1902	C	NADH-dependent flavin oxidoreductase
AFR72478.1	+	-	+	-	+	+	-	+	+	-	-	-	-	-	NA	NA	hypothetical protein C248_0465
ADI96955.1	-	-	-	-	-	+	-	-	-	-	-	-	-	-	NA	NA	pathogenicity island protein
AFR72479.1	+	-	+	-	+	-	-	+	-	-	-	-	-	-	NA	NA	hypothetical protein C248_0466
ADI96961.1	-	-	-	-	-	+	-	-	-	-	-	-	-	-	NA	NA	pathogenicity island protein

ADI96962.1	-	-	-	-	-	+	-	-	-	-	-	-	-	-	NA	NA	pathogenicity island protein, putative terminase
ADI96964.1	-	-	-	-	-	+	-	-	-	-	-	-	+	-	NA	NA	pathogenicity island protein
ADI96965.1	-	-	-	-	-	+	-	-	+	-	-	-	+	-	NA	NA	pathogenicity island protein
ADI96966.1	-	-	-	-	-	+	-	-	-	-	-	-	+	-	NA	NA	staphylococcal enterotoxin C-ovine
ADI96967.1	-	-	-	-	+	+	+	-	-	-	-	-	+	-	NA	NA	staphylococcal enterotoxin L
ADI97584.1	-	-	-	-	-	+	-	-	-	-	-	-	-	-	NA	NA	hypothetical protein SAOV_1071c
ADI97585.1	-	-	-	-	-	+	-	-	-	-	-	-	-	-	NA	NA	hypothetical protein SAOV_1072c
ADI97586.1	-	-	-	-	-	+	-	-	-	-	-	-	-	-	NA	NA	hypothetical protein SAOV_1073c
ADI97588.1	-	-	-	-	-	+	-	-	-	-	-	-	-	-	NA	NA	hypothetical protein SAOV_1075
ADI97589.1	-	-	-	-	+	+	-	-	-	-	-	-	-	-	NA	NA	hypothetical phage-related protein
ADI97590.1	-	-	-	-	-	+	-	-	+	-	-	-	-	-	COG3617	X	prophage, antirepressor, putative
ADI97598.1	-	-	-	-	-	+	-	+	-	+	+	+	+	-	NA	NA	hypothetical phage-related protein
ADI97599.1	-	-	-	-	-	+	-	-	-	-	-	-	-	-	NA	NA	hypothetical phage-related protein
ADI97606.1	-	-	-	-	-	+	-	-	-	-	-	-	-	-	NA	NA	hypothetical protein SAOV_1093
ADI97607.1	-	-	-	-	-	+	-	-	-	-	-	-	-	-	NA	NA	hypothetical protein SAOV_1094
AFR72484.1	+	-	+	-	-	-	-	-	-	-	-	-	-	-	NA	NA	hypothetical protein C248_0471
AFR72039.1	+	-	+	-	-	+	-	+	-	-	+	-	+	+	COG1235	P	metallo-beta-lactamase superfamily protein
ADI97627.1	-	-	-	-	-	+	-	-	-	-	-	-	-	-	COG5412	X	Phage tail length tape-measure protein
ADI97628.1	-	-	-	-	-	+	-	-	-	-	-	-	-	-	NA	NA	Phage tail length tape-measure protein
ADI97632.1	-	-	-	-	+	+	-	-	-	+	-	-	-	-	NA	NA	hypothetical phage protein
ADI97633.1	-	-	-	-	-	+	+	-	-	-	-	-	+	-	NA	NA	Enterotoxin, phage associated
ADI97635.1	-	-	-	-	-	+	-	-	-	-	-	-	-	-	NA	NA	Phage amidase
ADI97665.1	-	-	-	-	+	+	-	-	-	-	-	-	-	-	NA	NA	hypothetical protein SAOV_1152
ADI97666.1	-	-	-	-	-	+	-	-	-	-	-	-	-	-	NA	NA	hypothetical protein SAOV_1153c
ADI97670.1	-	-	-	-	-	+	-	-	-	-	-	-	-	-	NA	NA	hypothetical protein SAOV_1157c
ADI97675.1	-	-	-	-	+	+	-	-	+	-	-	-	+	-	NA	NA	hypothetical protein SAOV_1162
ADI97683.1	-	-	-	-	-	+	-	-	-	-	-	-	+	-	NA	NA	probable membrane protein
AFR72489.1	+	-	+	-	-	-	-	-	-	-	-	-	-	-	NA	NA	Hypothetical protein C248_0476
ADI97947.1	-	-	-	-	-	+	-	-	-	-	-	-	-	-	NA	NA	conserved hypothetical protein
AFR72491.1	+	-	-	-	-	-	-	-	-	-	-	-	-	-	NA	NA	hypothetical protein C248_0478
ADI98288.1	-	-	-	-	-	+	-	-	-	-	+	-	-	-	COG1131	V	ABC transporter, ATP-binding protein
ADI98289.1	-	-	-	-	-	+	-	-	-	-	+	-	-	-	NA	NA	putative ABC transporter permease protein
ADI98290.1	-	-	-	-	-	+	-	-	-	-	+	-	-	-	NA	NA	conserved hypothetical protein
ADI98291.1	-	-	-	-	-	+	-	-	-	-	-	-	-	-	NA	NA	hypothetical protein SAOV_1787
ADI98295.1	-	-	-	-	-	+	-	-	+	-	-	-	-	-	NA	NA	hypothetical protein SAOV_1791
ADI98296.1	-	-	-	-	-	+	-	-	-	-	-	-	-	-	NA	NA	hypothetical protein SAOV_1792
ADI98299.1	-	-	-	-	+	+	-	+	+	-	+	-	+	-	COG3591	E	serine proteinase
ADI98300.1	-	-	-	-	+	+	-	+	-	-	+	-	+	-	COG3591	E	serine proteinase
ADI98301.1	-	-	-	-	+	+	-	+	+	-	+	-	+	-	COG3591	E	serine proteinase
ADI98302.1	-	-	-	-	+	+	-	+	+	-	-	-	+	-	NA	NA	beta-lactamase
ADI98303.1	-	-	-	-	-	+	-	+	-	-	-	-	+	-	NA	NA	putative lantibiotic ABC transporter protein
ADI98304.1	-	-	-	-	-	+	-	+	-	-	-	-	+	-	NA	NA	putative lantibiotic ABC transporter protein
ADI98305.1	-	-	-	-	-	+	-	+	-	-	-	-	+	-	COG1131	V	epidermin immunity protein F
ADI98306.1	-	-	-	-	-	+	-	+	-	-	-	-	+	-	COG1404	O	probable serine protease precursor
ADI98307.1	-	-	-	-	-	+	-	+	-	-	-	-	+	-	COG0452	H	Epidermin biosynthesis protein epiD
ADI98308.1	-	-	-	-	-	+	-	+	-	-	-	-	+	-	NA	NA	Epidermin biosynthesis protein epiC
ADI98309.1	-	-	-	-	-	+	-	+	-	-	-	-	+	-	NA	NA	epidermin biosynthesis protein EpiB
ADI98310.1	-	-	-	-	-	+	-	+	-	-	-	-	+	-	NA	NA	hypothetical protein SAOV_1807
ADI98311.1	-	-	-	-	-	+	-	-	-	-	-	-	+	-	NA	NA	hypothetical protein SAOV_1808
ADI98314.1	-	-	-	-	-	+	-	+	-	-	-	-	+	-	NA	NA	hypothetical protein SAOV_1811
ADI98315.1	-	-	-	-	+	+	-	+	+	-	+	-	+	-	NA	NA	leukotoxin D subunit

ADI98316.1	-	-	-	-	+	+	+	-	+	-	-	-	+	-	NA	NA	hypothetical protein SAOV_1814
ADI98317.1	-	-	-	-	+	+	+	-	+	-	-	-	+	-	NA	NA	hypothetical protein SAOV_1815
ADI98318.1	-	-	-	-	+	+	+	+	+	-	-	-	-	-	NA	NA	hypothetical protein SAOV_1816
AFR72040.1	+	-	+	-	-	+	-	+	-	-	+	-	+	+	COG0737	FV	putative 5'-nucleotidase
ADI98372.1	-	-	-	-	+	+	+	+	+	-	+	-	+	-	NA	NA	leukocidin chain lukM precursor
ADI98373.1	-	-	-	-	-	+	-	-	-	-	+	-	-	-	NA	NA	lytic enzyme
ADI98374.1	-	-	-	-	-	+	-	-	-	+	+	-	-	-	COG5546	S	phage-associated holin
ADI98381.1	-	-	-	-	+	+	-	-	-	-	+	-	-	-	NA	NA	hypothetical protein SAOV_1919c
ADI98382.1	-	-	-	-	+	+	-	-	-	-	+	-	-	-	NA	NA	hypothetical protein SAOV_1920c
ADI98383.1	-	-	-	-	+	+	-	-	-	-	+	-	-	-	NA	NA	hypothetical protein SAOV_1921c
ADI98384.1	-	-	-	-	+	+	-	-	-	-	+	-	-	-	NA	NA	hypothetical protein SAOV_1922c
ADI98385.1	-	-	-	-	+	+	-	-	-	-	+	-	-	-	NA	NA	hypothetical phage related protein
ADI98386.1	-	-	-	-	-	+	-	-	-	-	-	-	-	-	NA	NA	hypothetical protein SAOV_1924c
ADI98387.1	-	-	-	-	+	+	-	-	-	-	+	+	-	-	NA	NA	DNA packaging protein
ADI98388.1	-	-	-	-	+	+	-	-	-	-	+	+	-	-	NA	NA	hypothetical protein SAOV_1926c
ADI98389.1	-	-	-	-	+	+	-	-	-	-	+	+	-	-	NA	NA	Phage capsid protein
ADI98390.1	-	-	-	-	+	+	-	-	-	-	+	+	-	-	NA	NA	Prohead protease
ADI98391.1	-	-	-	-	+	+	-	-	-	-	+	+	-	-	NA	NA	Phage portal protein
ADI98392.1	-	-	-	-	+	+	-	-	-	-	+	+	-	-	NA	NA	phi PVL orf 3-like protein-related protein
ADI98393.1	-	-	-	-	+	+	-	-	-	-	+	+	-	-	COG4626	X	Terminase large subunit, phage associated
ADI98394.1	-	-	-	-	+	+	-	-	-	-	+	+	-	-	NA	NA	Phage Terminase Small Subunit
ADI98395.1	-	-	-	-	+	+	-	-	-	-	+	+	-	-	COG1403	V	hypothetical protein SAOV_1933c
ADI98396.1	-	-	-	-	+	+	-	-	-	-	+	+	-	-	NA	NA	hypothetical phage-related protein
AFR72498.1	+	-	-	-	-	-	-	-	-	-	-	-	-	-	NA	NA	putative exported protein
AFR72499.1	+	-	-	-	-	-	-	-	-	-	-	-	-	-	NA	NA	hypothetical protein C248_0486
ADI98412.1	-	-	-	-	-	+	-	-	-	-	-	-	-	-	NA	NA	DNA helicase, phage-associated
ADI98416.1	-	-	-	-	-	+	-	-	-	+	-	-	-	-	NA	NA	hypothetical protein SAOV_1954c
ADI98419.1	-	-	-	-	+	+	+	+	+	-	+	+	-	-	NA	NA	hypothetical protein SAOV_1957c
ADI98420.1	-	-	-	-	+	+	+	+	+	-	+	+	-	-	NA	NA	hypothetical protein SAOV_1958c
ADI98421.1	-	-	-	-	+	+	-	-	+	-	-	-	-	-	NA	NA	hypothetical protein SAOV_1959c
ADI98423.1	-	-	-	-	-	+	-	-	-	-	-	-	-	-	NA	NA	hypothetical protein SAOV_1961c
ADI98424.1	-	-	-	-	-	+	-	-	-	-	-	-	-	-	NA	NA	hypothetical protein SAOV_1962c
ADI98425.1	-	-	-	-	-	+	-	-	-	-	+	-	-	-	NA	NA	hypothetical protein SAOV_1963
ADI98426.1	-	-	-	-	-	+	-	-	-	-	-	-	-	-	NA	NA	hypothetical phage-related protein
ADI98428.1	-	-	-	-	-	+	-	-	-	-	-	-	-	-	NA	NA	hypothetical protein SAOV_1966
ADI98429.1	-	-	-	-	+	+	-	-	-	+	-	-	-	+	NA	NA	hypothetical protein SAOV_1967c
ADI98430.1	-	-	-	-	-	+	-	-	-	-	-	-	-	-	NA	NA	hypothetical protein SAOV_1968c
ADI98431.1	-	-	-	-	+	+	-	-	+	+	-	-	-	-	NA	NA	hypothetical protein SAOV_1969c
ADI98433.1	-	-	-	-	-	+	-	-	-	+	-	-	-	-	NA	NA	hypothetical protein SAOV_1971
ADI98435.1	-	-	-	-	-	+	-	-	-	-	+	+	-	-	NA	NA	hypothetical protein SAOV_1973
ADI98436.1	-	-	-	-	+	+	+	+	-	-	+	+	-	-	NA	NA	prophage integrase
ADI98503.1	-	-	-	-	-	+	-	-	+	-	+	-	-	-	NA	NA	probable leukocidin S subunit
ADI98504.1	-	-	-	-	-	+	-	-	+	-	+	-	+	-	COG0624	E	probable succinyldiaminopimelate desuccinylase
ADI98505.1	-	-	-	-	-	+	-	-	-	-	-	-	-	-	COG0697	GER	Permease of the drug/metabolite transporter (DMT)
ADI98506.1	-	-	-	-	-	+	-	-	-	-	-	-	+	-	COG0156	H	5-aminolevulinate synthase
ADI98507.1	-	-	-	-	-	+	-	-	+	-	+	-	+	-	COG0168	P	probable sodium transport protein
ADI98508.1	-	-	-	-	-	+	-	-	-	-	-	-	+	-	NA	NA	transposase
ADI98509.1	-	-	-	-	-	+	-	-	+	-	+	-	+	-	NA	NA	phage-like protein
ADI98510.1	-	-	-	-	-	+	-	-	+	-	+	-	+	+	NA	NA	hypothetical protein SAOV_2049
ADI98511.1	-	-	-	-	-	+	-	-	-	-	-	-	-	-	NA	NA	hypothetical protein SAOV_2050
ADI98517.1	-	-	-	-	-	+	-	-	-	-	-	-	-	-	NA	NA	hypothetical protein SAOV_2056c

ADI98522.1	-	-	-	-	-	+	-	-	-	-	-	-	+	-	NA	NA	hypothetical protein SAOV_2061c
ADI98523.1	-	-	-	-	-	+	-	-	-	-	-	-	-	-	NA	NA	putative prophage antirepressor
ADI98524.1	-	-	-	-	+	+	-	-	-	-	-	-	-	-	NA	NA	putative transcriptional regulator
ADI98525.1	-	-	-	-	-	+	-	-	-	-	-	-	-	-	COG1396	K	putative transcriptional regulator
ADI98526.1	-	-	-	-	-	+	-	-	-	-	-	-	+	-	COG0582	LX	probable integrase
ADI98527.1	-	-	-	-	-	+	-	-	-	-	+	-	+	-	COG0459	O	60 kDa chaperonin protein
AFR72041.1	+	-	+	-	-	-	-	+	-	-	+	-	+	+	COG1576	J	hypothetical protein C248_0027
ADI98883.1	-	-	-	-	-	+	-	-	-	-	-	-	+	-	NA	NA	hypothetical protein SAOV_2443c
ADI98893.1	-	-	-	-	+	+	+	+	-	-	-	-	+	-	COG4115	V	conserved hypothetical protein
ADI98894.1	-	-	-	-	+	+	+	+	-	-	+	-	+	-	COG2161	V	hypothetical protein SAOV_2454c
ADI98980.1	-	-	-	-	+	+	-	-	+	-	+	-	-	-	NA	NA	permease, putative
ADI98990.1	-	-	-	-	-	+	-	-	-	-	-	-	-	-	NA	NA	hypothetical protein SAOV_2551c
ADI98991.1	-	-	-	-	-	+	-	-	-	-	-	-	-	-	NA	NA	hypothetical protein SAOV_2552c
ACY09979.1	-	-	-	-	+	-	-	-	-	-	-	-	-	-	NA	NA	hypothetical protein SAAV_0030
ACY09980.1	-	-	-	-	+	-	-	-	-	-	-	-	-	-	NA	NA	conserved hypothetical protein
ACY09993.1	-	-	-	-	+	-	-	-	-	-	-	-	-	-	NA	NA	hypothetical protein SAAV_0044
ACY10313.1	-	-	-	-	+	-	-	+	-	-	-	-	-	-	NA	NA	superantigen-like protein
ACY10325.1	-	-	-	-	+	-	-	-	-	-	+	-	-	-	NA	NA	putative lipoprotein
ACY10326.1	-	-	-	-	+	-	-	+	-	-	+	-	-	-	NA	NA	putative lipoprotein
ACY10327.1	-	-	-	-	+	-	-	-	-	-	+	-	+	-	NA	NA	putative lipoprotein
ACY10330.1	-	-	-	-	+	-	-	-	-	-	-	-	-	-	NA	NA	conserved hypothetical protein
ACY10703.1	-	-	-	-	+	-	-	+	-	-	+	-	-	-	COG1464	P	ABC transporter, substrate-binding protein
AFR72514.1	+	-	+	-	+	-	-	+	-	-	+	-	+	-	COG0523	R	putative cobalamin synthesis protein
ACY10705.1	-	-	-	-	+	-	-	-	-	-	-	-	-	-	NA	NA	SAP domain protein
ACY10706.1	-	-	-	-	+	-	-	+	-	-	-	-	+	-	NA	NA	conserved hypothetical protein
ACY10711.1	-	-	-	-	+	-	-	+	-	-	-	-	+	-	NA	NA	pathogenicity island protein
ACY10713.1	-	-	-	-	+	-	-	-	-	-	-	-	+	-	NA	NA	pathogenicity island protein ORF15
ACY10714.1	-	-	-	-	+	-	-	-	-	-	-	-	+	-	COG5519	S	pathogenicity island protein ORF 14/13
AFR72515.1	+	-	+	-	+	-	-	+	-	-	+	-	+	-	COG1009	CP	Putative NADH Ubiquinone plastoquinone protein
AFR72042.1	+	-	-	-	-	-	-	-	-	-	-	-	-	-	COG1397	O	ADP-ribosylglycohydrolase
ACY10716.1	-	-	-	-	+	-	-	+	+	-	-	-	+	-	NA	NA	pathogenicity island protein ORF11
ACY10717.1	-	-	-	-	+	-	-	+	+	-	-	-	+	-	NA	NA	pathogenicity island protein ORF10
ACY10718.1	-	-	-	-	+	-	-	-	+	-	-	-	+	-	NA	NA	pathogenicity island protein ORF9
ACY10719.1	-	-	-	-	+	-	-	-	+	-	-	-	+	-	NA	NA	pathogenicity island protein ORF8
ACY10721.1	-	-	-	-	+	-	-	+	+	-	-	-	+	-	NA	NA	pathogenicity island protein ORF6
ACY10722.1	-	-	-	-	+	-	-	+	+	-	-	-	+	-	COG3728	X	pathogenicity island protein ORF5; phage terminase
ACY10723.1	-	-	-	-	+	-	-	+	-	-	-	-	-	-	NA	NA	hypothetical protein SAAV_0806
ACY10724.1	-	-	-	-	+	-	-	+	-	-	-	-	-	-	NA	NA	conserved hypothetical protein
AFR72516.1	+	-	+	-	+	-	-	+	-	-	+	-	+	-	COG3002	S	hypothetical protein C248_0503
ACY10725.1	-	-	-	-	+	-	-	+	-	-	-	-	-	-	NA	NA	conserved hypothetical protein
ACY10727.1	-	-	-	-	+	-	-	-	-	-	-	-	-	-	NA	NA	hypothetical protein SAAV_0810
ACY10728.1	-	-	-	-	+	-	-	+	-	-	-	-	-	-	COG3237	S	hypothetical protein SAAV_0811
ACY10736.1	-	-	-	-	+	-	-	-	-	-	-	-	-	-	NA	NA	DNA adenine methylase
ACY10737.1	-	-	-	-	+	-	-	-	-	-	-	-	-	-	COG0338	L	putative methylase
ACY10738.1	-	-	-	-	+	-	-	-	-	-	-	-	-	-	NA	NA	conserved hypothetical phage protein
AFR72517.1	+	-	+	-	+	-	-	+	-	-	-	-	+	-	COG5609	S	hypothetical protein C248_0504
ACY10743.1	-	-	-	-	+	-	-	-	-	-	-	-	+	-	NA	NA	conserved hypothetical phage protein
ACY10745.1	-	-	-	-	+	-	-	-	-	-	-	-	-	-	NA	NA	conserved hypothetical phage protein
ACY10746.1	-	-	-	-	+	-	-	-	-	-	-	-	-	-	NA	NA	conserved hypothetical phage protein
ACY10747.1	-	-	-	-	+	-	+	+	-	-	+	-	-	-	NA	NA	conserved hypothetical phage protein
AFR72518.1	+	-	+	-	+	-	-	+	-	-	-	-	+	-	NA	NA	putative membrane protein

ACY10753.1	-	-	-	-	+	-	-	-	-	-	-	-	-	-	NA	NA	hypothetical phage protein
ACY10754.1	-	-	-	-	+	-	-	-	-	-	-	-	-	-	NA	NA	conserved hypothetical phage protein
ACY10755.1	-	-	-	-	+	-	-	-	-	-	-	-	-	-	NA	NA	putative phage replication protein
ACY10756.1	-	-	-	-	+	-	+	-	+	+	+	-	-	-	COG1484	L	DnaC
ACY10757.1	-	-	-	-	+	-	+	-	+	+	-	-	-	-	NA	NA	conserved hypothetical phage protein
ACY10760.1	-	-	-	-	+	-	-	-	-	-	-	-	-	-	NA	NA	PVL ORF-50 family protein
AFR72519.1	+	-	+	-	+	-	-	+	-	-	+	-	-	-	COG0671	I	putative membrane protein
ACY10761.1	-	-	-	-	+	-	-	-	-	-	-	-	-	-	NA	NA	conserved hypothetical phage prtotein
ACY10763.1	-	-	-	-	+	-	-	-	-	-	-	-	-	-	NA	NA	conserved hypothetical phage protein
ACY10766.1	-	-	-	-	+	-	-	-	-	-	+	+	-	-	NA	NA	conserved hypothetical phage protein
AFR72520.1	+	-	+	-	+	-	-	+	-	-	+	-	-	-	COG1647	Q	hypothetical protein C248_0507
AFR72521.1	+	-	+	-	+	-	-	+	-	-	+	-	-	-	COG0733	R	sodium:neurotransmitter symporter family protein
ACY10790.1	-	-	-	-	+	-	-	-	-	-	-	-	-	-	NA	NA	holin, SPP1 family
AFR72522.1	+	-	+	-	+	-	-	+	-	-	+	-	-	-	COG0031	E	pyridoxal-phosphate dependent enzyme
ACY10791.1	-	-	-	-	+	-	-	-	-	-	-	-	-	-	NA	NA	phage amidase
ACY10792.1	-	-	-	-	+	-	-	-	-	-	-	-	-	-	NA	NA	Ear-like protein
ACY10793.1	-	-	-	-	+	-	-	-	+	-	-	-	-	-	NA	NA	hypothetical phage protein
ACY10794.1	-	-	-	-	+	-	-	-	-	-	-	-	-	-	NA	NA	hypothetical phage protein
ACY10795.1	-	-	-	-	+	-	-	-	+	-	-	-	-	-	NA	NA	hypothetical phage protein
AFR72523.1	+	-	+	-	+	-	-	+	-	-	+	-	-	-	COG0626	E	putative Cys/Met metabolism PLP-dependent enzyme
ACY11036.1	-	-	-	-	+	-	-	-	-	-	-	-	-	-	NA	NA	conserved hypothetical protein
ACY11038.1	-	-	-	-	+	-	+	-	+	-	-	-	-	-	COG3464	X	IS1181 transposase
AFR72524.1	+	-	+	-	+	-	-	+	-	-	+	-	-	-	COG1135	E	ABC transporter ATP-binding protein
AFR72525.1	+	-	+	-	+	-	-	+	-	-	+	-	-	-	COG2011	E	putative transport system membrane protein
AFR72043.1	+	-	-	-	-	-	-	-	-	-	-	-	-	-	NA	NA	putative permease
ACY11317.1	-	-	-	-	+	-	-	-	+	-	-	-	-	-	NA	NA	hypothetical protein SAAV_1415
ACY11319.1	-	-	-	-	+	-	-	-	-	-	-	-	-	-	NA	NA	surface protein, ECM binding protein-like protein
ACY11320.1	-	-	-	-	+	-	-	-	-	-	-	-	-	-	NA	NA	surface protein, ECM binding protein-like protein
ACY11321.1	-	-	-	-	+	-	-	-	-	-	-	-	-	-	COG5281	X	surface protein, ECM binding protein-like protein
ACY11701.1	-	-	-	-	+	-	-	-	-	-	-	-	-	-	COG2801	X	transposase
ACY11702.1	-	-	-	-	+	-	-	-	-	-	-	-	-	-	COG4584	X	transposase
ACY11706.1	-	-	-	-	+	-	-	-	+	-	-	-	-	-	COG3591	E	serine protease SplD, putative
ACY11709.1	-	-	-	-	+	-	-	+	+	-	+	-	-	-	COG3591	E	serine protease SplA
ACY11711.1	-	-	-	-	+	-	-	-	-	-	-	-	-	-	NA	NA	hypothetical protein SAAV_1823
ACY11712.1	-	-	-	-	+	-	-	-	+	-	-	-	-	-	NA	NA	epidermin immunity protein F
ACY11713.1	-	-	-	-	+	-	-	-	+	-	-	-	-	-	NA	NA	conserved hypothetical protein
ACY11714.1	-	-	-	-	+	-	-	-	+	-	-	-	-	-	NA	NA	conserved hypothetical protein
ACY11715.1	-	-	-	-	+	-	-	-	-	-	-	-	-	-	NA	NA	hypothetical protein SAAV_1827
ACY11718.1	-	-	-	-	+	-	+	-	-	-	-	-	-	-	NA	NA	hypothetical protein SAAV_1830
ACY11722.1	-	-	-	-	+	-	+	-	-	-	-	-	-	-	NA	NA	staphylococcal enterotoxin G
ACY11723.1	-	-	-	-	+	-	-	-	-	-	-	-	-	-	NA	NA	staphylococcal enterotoxin N
ACY11724.1	-	-	-	-	+	-	+	-	-	-	-	-	-	-	NA	NA	staphylococcal enterotoxin Yent1
ACY11725.1	-	-	-	-	+	-	+	-	-	-	-	-	-	-	NA	NA	staphylococcal enterotoxin Yent2
ACY11727.1	-	-	-	-	+	-	+	-	-	-	-	-	-	-	NA	NA	staphylococcal enterotoxin M
ACY11728.1	-	-	-	-	+	-	+	-	-	-	-	-	+	-	NA	NA	staphylococcal enterotoxin O
AFR72582.1	+	-	-	+	-	-	+	-	-	-	-	-	-	-	NA	NA	hypothetical protein C248_0573
ACY11887.1	-	-	-	-	+	-	-	-	-	-	-	-	-	-	NA	NA	conserved hypothetical phage protein
ACY11890.1	-	-	-	-	+	-	-	-	-	-	-	-	-	-	NA	NA	conserved hypothetical phage protein
ACY11892.1	-	-	-	-	+	-	-	-	-	-	-	-	-	-	NA	NA	hypothetical phage protein
ACY12283.1	-	-	-	-	+	-	-	-	-	-	+	+	-	-	COG2223	P	nitrite extrusion protein
AFR72044.1	+	-	-	-	-	-	-	-	-	-	-	-	-	-	COG0524	G	putative kinase

ACY12392.1	-	-	-	-	+	-	-	-	-	-	-	-	-	-	NA	NA	LPXTG-motif protein
ACY12393.1	-	-	-	-	+	-	+	-	+	-	-	-	-	-	COG1846	K	staphylococcal accessory regulator T
ACY12394.1	-	-	-	-	+	-	+	-	+	-	-	-	-	-	COG1846	K	Staphylococcal accessory regulator U
ACY12397.1	-	-	-	-	+	-	-	-	-	-	-	-	-	-	NA	NA	hypothetical protein SAAV_2567
ACY12604.1	-	-	-	-	+	-	-	-	-	-	-	-	-	-	NA	NA	hypothetical protein SAAV_2775
ACY12606.1	-	-	-	-	+	-	-	-	-	-	-	-	-	-	COG1476	K	transcriptional regulator, Cro/CI family
AFR72869.1	+	-	-	-	-	-	-	-	-	-	-	-	-	-	NA	NA	hypothetical protein C248_0875
ACY12607.1	-	-	-	-	+	-	-	-	-	-	-	-	-	-	NA	NA	hypothetical protein SAAV_2778
ACY12608.1	-	-	-	-	+	-	-	-	-	-	-	-	-	-	NA	NA	hypothetical protein SAAV_2779
ACY12609.1	-	-	-	-	+	-	-	-	-	-	-	-	-	-	COG1475	D	ParB family chromosome partioning protein
ACY12610.1	-	-	-	-	+	-	-	-	-	-	-	-	-	-	COG0357	J	16S rRNA methyltransferase GidB
ACY12611.1	-	-	-	-	+	-	-	-	-	-	-	-	-	-	COG0445	J	tRNA uridine 5-carboxymethylaminomethyl modification enzyme GidA
ACY12612.1	-	-	-	-	+	-	-	-	-	-	-	-	-	-	COG0486	J	tRNA modification GTPase TrmE
ACY12613.1	-	-	-	-	+	-	-	-	-	-	-	-	-	-	COG0594	J	ribonuclease P protein component
ACY12614.1	-	-	-	-	+	-	-	-	-	-	-	-	-	-	COG0230	J	50S ribosomal protein L34
AFR72870.1	+	-	+	+	-	-	+	-	-	-	-	-	-	-	NA	NA	Hypothetical protein C248_0876
ASC50314.1	-	-	-	-	-	-	+	-	-	-	-	-	-	-	COG0557	K	ribonuclease R
ASC50315.1	-	-	-	-	-	-	+	-	-	-	-	-	+	-	COG0691	O	SsrA-binding protein
ASC50316.1	-	-	-	-	-	-	+	-	-	-	-	-	-	-	NA	NA	hypothetical protein A2V17_04080
ASC50317.1	-	-	-	-	-	-	+	-	+	-	-	-	-	-	NA	NA	hypothetical protein A2V17_04085
ASC50318.1	-	-	-	-	-	-	+	-	-	-	-	-	-	-	NA	NA	hypothetical protein A2V17_04090
ASC50323.1	-	-	-	-	-	-	+	-	-	-	-	-	-	-	NA	NA	clumping factor A
AFR72874.1	+	-	-	-	-	-	-	-	-	-	-	-	-	-	NA	NA	Putative coagulase
AFR72875.1	+	-	-	-	-	-	-	-	-	-	-	-	-	-	NA	NA	von Willebrand factor binding protein
ASC50361.1	-	-	-	-	-	-	+	-	-	-	-	-	-	-	NA	NA	hypothetical protein A2V17_04305
ASC50362.1	-	-	-	-	-	-	+	-	-	-	-	-	-	-	COG0463	M	glycosyl transferase family A
ASC50363.1	-	-	-	-	-	-	+	+	-	-	-	-	-	-	COG1396	K	DNA-binding protein
ASC50364.1	-	-	-	-	-	-	+	-	-	-	-	-	-	-	NA	NA	transcriptional regulator
ASC52276.1	-	-	-	-	-	-	+	-	-	-	-	-	-	-	NA	NA	hypothetical protein A2V17_04325
AFR72045.1	+	-	-	-	-	-	-	-	-	-	-	-	+	+	COG3316	X	putative transposase
ASC50366.1	-	-	-	-	-	-	+	+	-	-	-	-	-	-	NA	NA	hypothetical protein A2V17_04340
ASC50375.1	-	-	-	-	-	-	+	-	-	+	+	-	-	-	NA	NA	replication protein
ASC50386.1	-	-	-	-	-	-	+	-	+	-	+	-	-	-	NA	NA	hypothetical protein A2V17_04445
AFR72879.1	+	-	-	-	-	-	-	-	-	-	-	-	-	+	NA	NA	Hypothetical protein C248_0885
ASC50944.1	-	-	-	-	-	-	+	-	-	-	-	-	-	-	NA	NA	hypothetical protein A2V17_07350
ASC50945.1	-	-	-	-	-	-	+	-	-	-	-	-	-	-	NA	NA	hypothetical protein A2V17_07355
AFR72046.1	+	-	-	-	-	-	-	-	-	-	-	-	-	-	NA	NA	HMG-CoA synthase
AFR72028.1	+	-	+	-	-	-	-	-	-	-	-	-	+	-	COG2021	E	putative hydrolase
AFR72887.1	+	-	-	-	-	-	-	-	-	-	-	-	-	-	COG0406	G	phosphoglycerate mutase family protein
ASC51455.1	-	-	-	-	-	-	+	-	-	-	-	-	-	-	NA	NA	hypothetical protein A2V17_10200
ASC51456.1	-	-	-	-	-	-	+	-	+	+	-	-	-	-	NA	NA	hypothetical protein A2V17_10205
ASC51569.1	-	-	-	-	-	-	+	-	-	-	-	-	-	-	COG0394	T	arsenate reductase (thioredoxin)
ASC51570.1	-	-	-	-	-	-	+	-	-	-	-	-	-	-	NA	NA	ATP-binding protein
ASC51571.1	-	-	-	-	-	-	+	-	-	-	-	-	-	-	COG2801	X	transposase
ASC51572.1	-	-	-	-	-	-	+	-	-	-	-	-	+	-	COG1961	L	transposon DNA-invertase
ASC51573.1	-	-	-	-	-	-	+	+	-	-	-	-	-	-	COG3682	K	penicillinase repressor BlaI
ASC51574.1	-	-	-	-	-	-	+	+	-	-	-	-	-	-	COG2602	V	BlaR1 family beta-lactam sensor/signal transducer
ASC51575.1	-	-	-	-	-	-	+	+	-	-	-	-	-	-	COG2367	V	BlaZ family class A beta-lactamase
ASC51576.1	-	-	-	-	-	-	+	-	-	-	-	-	-	-	NA	NA	hypothetical protein A2V17_10870
ASC51577.1	-	-	-	-	-	-	+	-	-	-	-	-	-	-	COG0640	K	transcriptional regulator
ASC51578.1	-	-	-	-	-	-	+	-	-	-	-	-	-	-	COG0701	S	permease

ASC51579.1	-	-	-	-	-	-	+	-	-	-	-	-	-	-	NA	NA	methyltransferase
AFR72892.1	+	-	-	-	-	-	-	-	-	-	-	-	-	-	COG0778	C	nitroreductase family protein
AFR72964.1	+	-	-	-	-	-	-	-	-	-	-	-	-	+	COG0179	Q	fumarylacetoacetate (FAA) hydrolase family protein
AFR72965.1	+	-	-	-	-	-	-	-	-	-	-	-	-	+	NA	NA	putative membrane protein
ASC52030.1	-	-	-	-	-	-	+	-	+	-	-	+	-	-	NA	NA	hypothetical protein A2V17_13270
ASC52031.1	-	-	-	-	-	-	+	-	-	-	-	-	-	-	NA	NA	Accumulation-associated protein
AFR72047.1	+	-	-	-	-	-	-	-	-	-	-	-	-	-	NA	NA	putative membrane protein
CCC86798.1	-	-	-	-	-	-	-	+	-	-	-	-	-	-	NA	NA	putative membrane protein
CCC86799.1	-	-	-	-	-	-	-	+	-	-	-	-	-	-	NA	NA	putative exported protein
CCC86800.1	-	-	-	-	-	-	-	+	-	-	-	-	-	-	NA	NA	conserved hypothetical protein
CCC86801.1	-	-	-	-	-	-	-	+	-	-	-	-	-	-	COG2003	L	conserved hypothetical protein
CCC86828.1	-	-	-	-	-	-	-	+	-	-	-	-	-	-	NA	NA	putative membrane protein
CCC86829.1	-	-	-	-	-	-	-	+	-	-	-	-	-	-	NA	NA	hypothetical protein SARLGA251_00600
CCC86830.1	-	-	-	-	-	-	-	+	+	-	-	-	-	-	NA	NA	hypothetical protein SARLGA251_00610
AFR72967.1	+	-	-	-	-	-	-	-	-	-	-	-	-	-	NA	NA	hypothetical protein C248_0973
CCC86837.1	-	-	-	-	-	-	-	+	-	-	-	-	-	-	COG0589	T	hypothetical protein SARLGA251_00680
CCC86840.1	-	-	-	-	-	-	-	+	-	-	-	-	-	-	COG2814	G	Major Facilitator Superfamily protein
CCC86841.1	-	-	-	-	-	-	-	+	-	-	-	-	-	-	COG0655	C	conserved hypothetical protein
AFR72969.1	+	-	-	-	-	-	-	-	-	-	-	-	-	-	NA	NA	hypothetical protein C248_0975
CCC86842.1	-	-	-	-	-	-	-	+	-	-	-	-	-	-	COG0583	K	LysR family regulatory protein
CCC87126.1	-	-	-	-	-	-	-	+	+	-	-	-	-	-	COG1683	S	conserved hypothetical protein
CCC87182.1	-	-	-	-	-	-	-	+	-	-	-	-	-	-	NA	NA	putative membrane protein
CCC87223.1	-	-	-	-	-	-	-	+	-	-	-	-	-	-	NA	NA	hypothetical protein SARLGA251_04550
AFR72048.1	+	-	-	-	-	-	-	-	-	-	-	-	-	+	COG0584	I	glycerophosphoryl diester phosphodiesterase
CCC87516.1	-	-	-	-	-	-	-	+	-	-	-	-	+	-	NA	NA	pathogenicity island protein
AFR72978.1	+	-	-	+	-	-	-	-	-	-	-	-	-	+	NA	NA	hypothetical protein C248_0984
CCC87522.1	-	-	-	-	-	-	-	+	-	-	-	-	-	-	NA	NA	pathogenicity island protein
CCC87523.1	-	-	-	-	-	-	-	+	-	-	-	-	-	-	NA	NA	pathogenicity island replication protein
CCC87524.1	-	-	-	-	-	-	-	+	-	-	-	-	-	-	NA	NA	pathogenicity island protein
CCC87527.1	-	-	-	-	-	-	-	+	-	-	-	-	-	-	NA	NA	pathogenicity island protein
CCC87528.1	-	-	-	-	-	-	-	+	-	-	-	-	-	-	NA	NA	pathogenicity island protein
CCC87536.1	-	-	-	-	-	-	-	+	-	-	-	-	-	-	NA	NA	pathogenicity island protein
CCC87538.1	-	-	-	-	-	-	-	+	-	-	-	-	-	-	COG2035	S	putative membrane protein
CCC87539.1	-	-	-	-	-	-	-	+	-	-	-	-	-	-	COG0396	O	ABC transporter ATP-binding protein
CCC87540.1	-	-	-	-	-	-	-	+	-	-	-	-	-	-	COG0719	O	conserved hypothetical protein
CCC87541.1	-	-	-	-	-	-	-	+	-	-	-	-	-	-	COG0520	E	putative selenocysteine lyase
CCC87542.1	-	-	-	-	-	-	-	+	-	-	-	-	-	-	COG0822	O	NifU-like protein
CCC87545.1	-	-	-	-	-	-	-	+	-	-	-	-	-	-	NA	NA	phage protein
CCC87547.1	-	-	-	-	-	-	-	+	-	-	-	-	-	-	NA	NA	phage DNA-binding protein
CCC87548.1	-	-	-	-	-	-	-	+	-	-	-	-	-	-	NA	NA	phage protein
CCC87549.1	-	-	-	-	-	-	-	+	-	-	-	-	-	-	NA	NA	phage protein
AFR72982.1	+	-	-	+	-	-	-	-	-	-	-	-	-	-	NA	NA	hypothetical protein C248_0988
CCC87565.1	-	-	-	-	-	-	-	+	-	-	-	-	-	-	NA	NA	phage protein
CCC87568.1	-	-	-	-	-	-	-	+	-	+	-	-	-	+	NA	NA	phage protein
CCC87582.1	-	-	-	-	-	-	-	+	-	-	-	-	-	-	NA	NA	phage protein
CCC87589.1	-	-	-	-	-	-	-	+	-	-	-	-	-	-	NA	NA	phage protein
AFR72986.1	+	-	-	-	-	-	-	-	-	-	-	-	-	-	NA	NA	hypothetical protein C248_0992
AFR72049.1	+	-	-	-	-	-	-	-	-	-	-	-	-	+	COG2030	I	acyl dehydratase
AFR72988.1	+	-	-	-	-	-	-	-	-	-	-	-	-	-	NA	NA	Putative transposase
CCC88454.1	-	-	-	-	-	-	-	+	-	-	-	-	-	-	COG0739	M	peptidase family M23/M37 protein
CCC88458.1	-	-	-	-	-	-	+	+	-	+	-	-	-	-	COG3591	E	serine protease

CCC88475.1	-	-	-	-	-	-	-	+	-	-	-	-	-	-	NA	NA	hypothetical protein SARLGA251_17100
AFR72993.1	+	-	-	-	-	-	-	-	-	-	-	-	-	-	NA	NA	hypothetical protein C248_0999
CCC89034.1	-	-	-	-	-	-	-	+	+	-	-	-	-	-	NA	NA	putative helicase
CCC89035.1	-	-	-	-	-	-	-	+	+	-	-	-	-	-	COG0494	V	putative NUDIX hydrolase
AFR73173.1	+	-	+	-	+	+	-	+	+	-	-	-	+	+	COG0622	R	hypothetical protein C248_1181
AFR73174.1	+	-	+	-	+	+	-	-	-	-	-	-	+	+	NA	NA	Hypothetical protein C248_1182
AFR72050.1	+	-	-	-	-	-	-	+	-	-	-	-	-	+	COG0768	DM	penicillin-binding protein 2 prime
SaureusN305_00073	-	-	-	-	-	-	-	-	+	-	-	-	+	-	NA	NA	T5orf172 domain protein
SaureusN305_00074	-	-	-	-	-	-	-	-	+	-	-	-	+	-	NA	NA	hypothetical protein
AFR73175.1	+	-	+	-	+	+	-	+	+	-	-	-	+	+	NA	NA	putative exported protein
SaureusN305_00083	-	-	-	-	-	-	-	-	+	-	-	-	-	-	COG3591	E	Serine protease SplC precursor
SaureusN305_00084	-	-	-	-	-	-	-	-	+	-	-	-	-	-	NA	NA	Serine protease SplC precursor
AFR73176.1	+	-	+	-	+	-	-	+	+	-	-	-	+	+	NA	NA	FPRL1 inhibitory protein
AFR73177.1	+	-	+	-	+	+	-	+	+	-	-	-	+	+	NA	NA	putative membrane protein
SaureusN305_00161	-	-	-	-	-	-	-	-	+	-	-	+	-	+	COG0624	E	Putative dipeptidase
SaureusN305_00368	-	-	-	-	-	-	-	-	+	-	-	-	-	-	NA	NA	hypothetical protein
SaureusN305_00371	-	-	-	-	-	-	-	-	+	-	-	-	-	-	NA	NA	Surface protein G precursor
AFR73178.1	+	-	+	-	+	+	-	+	+	-	-	-	+	+	NA	NA	fibrinogen-binding protein precursor
AFR73179.1	+	-	+	-	+	+	-	+	+	-	-	-	+	+	NA	NA	putative exported protein
SaureusN305_00384	-	-	-	-	-	-	-	-	+	+	-	-	+	-	NA	NA	hypothetical protein
SaureusN305_00385	-	-	-	-	-	-	-	-	+	-	-	-	+	-	NA	NA	putative sulfoacetate transporter SauU
SaureusN305_00386	-	-	-	-	-	-	-	-	+	-	-	-	+	-	COG0586	S	Inner membrane protein YqjA
SaureusN305_00626	-	-	-	-	-	-	-	-	+	-	-	-	-	-	COG0042	J	putative tRNA-dihydrouridine synthase
AFR73180.1	+	-	-	-	+	+	-	+	+	-	-	-	+	+	NA	NA	Hypothetical protein C248_1188
SaureusN305_00637	-	-	-	-	-	-	-	-	+	-	-	-	-	-	NA	NA	1-phosphatidylinositol phosphodiesterase precursor
AFR73181.1	+	-	-	-	+	+	-	+	+	-	-	-	+	+	NA	NA	hypothetical protein C248_1189
SaureusN305_00938	-	-	-	-	-	-	-	-	+	-	-	-	-	-	NA	NA	Helix-turn-helix
SaureusN305_00939	-	-	-	-	-	-	-	-	+	-	-	-	-	-	NA	NA	Helix-turn-helix
AFR73182.1	+	-	-	-	+	+	-	+	+	-	-	-	+	+	NA	NA	Alpha-hemolysin
SaureusN305_00948	-	-	-	-	-	-	-	-	+	-	-	-	-	-	NA	NA	hypothetical protein
SaureusN305_00949	-	-	-	-	-	-	-	-	+	-	-	-	-	-	NA	NA	Aminodeoxyfutalosine deaminase
SaureusN305_00952	-	-	-	-	-	-	-	-	+	-	-	-	-	-	NA	NA	hypothetical protein
SaureusN305_00953	-	-	-	-	-	-	-	-	+	-	-	-	-	-	NA	NA	hypothetical protein
SaureusN305_00955	-	-	-	-	-	-	-	-	+	-	-	-	-	-	NA	NA	hypothetical protein

AFR73183.1	+	-	+	+	+	+	-	+	+	-	-	-	+	+	NA	NA	hypothetical protein C248_1191
SaureusN305 _00960	-	-	-	-	-	-	-	-	+	-	-	-	-	-	NA	NA	hypothetical protein
AFR73184.1	+	-	+	-	+	+	-	+	+	-	-	-	+	+	NA	NA	exotoxin
AFR72051.1	+	-	-	-	-	-	-	-	-	-	-	-	-	+	COG3316	X	Transposase for insertion sequence element IS431
AFR73185.1	+	-	+	-	+	+	-	+	+	-	-	-	+	+	NA	NA	exotoxin
SaureusN305 _01293	-	-	-	-	-	-	-	-	+	-	-	-	-	-	NA	NA	Clumping factor A precursor
AFR73186.1	+	-	+	-	+	+	-	+	-	-	-	-	+	+	NA	NA	exotoxin
AFR73187.1	+	-	+	-	+	+	-	+	+	-	-	-	+	+	COG0078	E	ornithine carbamoyltransferase
AFR73188.1	+	-	+	-	+	+	-	+	+	-	-	-	+	+	COG0549	E	putative carbamate kinase
AFR73189.1	+	-	+	-	+	+	-	+	+	-	-	-	+	+	COG1288	R	putative membrane protein
AFR73190.1	+	-	+	-	+	-	-	+	+	-	-	-	-	+	NA	NA	putative membrane protein
SaureusN305 _01842	-	-	-	-	-	-	-	-	+	-	-	-	-	-	NA	NA	hypothetical protein
SaureusN305 _01843	-	-	-	-	-	-	-	-	+	-	-	-	-	-	NA	NA	hypothetical protein
SaureusN305 _01851	-	-	-	-	-	-	-	-	+	-	-	-	-	-	NA	NA	hypothetical protein
SaureusN305 _01852	-	-	-	-	-	-	-	-	+	-	-	-	-	-	COG0286	V	N-6 DNA Methylase
AFR73191.1	+	-	+	-	+	+	-	+	+	-	-	-	+	+	COG1275	V	exfoliative toxin A/B
SaureusN305 _01854	-	-	-	-	-	-	-	-	+	-	-	-	-	-	NA	NA	hypothetical protein
SaureusN305 _01856	-	-	-	-	-	-	-	-	+	-	-	-	-	-	NA	NA	hypothetical protein
SaureusN305 _01858	-	-	-	-	-	-	-	-	+	-	-	-	-	-	NA	NA	hypothetical protein
SaureusN305 _01860	-	-	-	-	-	-	-	-	+	-	-	-	-	-	NA	NA	hypothetical protein
AFR73192.1	+	-	+	-	+	+	-	+	+	-	-	-	+	+	NA	NA	putative DNA binding protein
SaureusN305 _01889	-	-	-	-	-	-	-	-	+	-	-	-	-	-	NA	NA	Phage tail repeat like protein
SaureusN305 _01890	-	-	-	-	-	-	-	-	+	-	-	-	-	-	NA	NA	hypothetical protein
AFR73193.1	+	-	+	-	+	+	-	+	+	-	-	-	-	+	NA	NA	antibacterial protein
SaureusN305 _01962	-	-	-	-	-	-	-	-	+	-	-	-	-	-	NA	NA	Tyrosine recombinase XerD
SaureusN305 _01963	-	-	-	-	-	-	-	-	+	-	-	-	-	-	COG0582	LX	site-specific tyrosine recombinase XerC
SaureusN305 _01964	-	-	-	-	-	-	-	-	+	-	+	-	-	-	NA	NA	Host cell surface-exposed lipoprotein
SaureusN305 _01965	-	-	-	-	-	-	-	-	+	-	-	-	-	-	NA	NA	hypothetical protein
SaureusN305 _01966	-	-	-	-	-	-	-	-	+	-	-	-	-	-	NA	NA	transcriptional repressor DicA
SaureusN305 _01968	-	-	-	-	-	-	-	-	+	-	-	-	-	-	NA	NA	hypothetical protein
SaureusN305 _01969	-	-	-	-	-	-	-	-	+	-	-	-	-	-	NA	NA	hypothetical protein
SaureusN305_ 1970	-	-	-	-	-	-	-	-	+	-	-	-	-	-	NA	NA	hypothetical protein
SaureusN305_	-	-	-	-	-	-	-	-	+	-	-	-	-	-	NA	NA	hypothetical protein

1971	-	-	-	-	-	-	-	-	+	-	-	-	-	-	NA	NA	hypothetical protein
AFR73194.1	+	-	+	-	+	+	-	+	+	-	-	-	-	+	NA	NA	Hypothetical protein C248_1203
AFR72052.1	+	-	-	-	-	-	-	-	-	-	-	-	-	-	COG3316	X	Transposase for insertion sequence element IS431
SaureusN305_ 1972	-	-	-	-	-	-	-	-	+	-	-	-	-	-	NA	NA	hypothetical protein
SaureusN305_ 1977	-	-	-	-	-	-	-	-	+	-	-	-	-	-	NA	NA	Bacteriophage Mu Gam like protein
SaureusN305_ 1978	-	-	-	-	-	-	-	-	+	+	-	-	-	-	NA	NA	hypothetical protein
SaureusN305_ 1979	-	-	-	-	-	-	-	-	+	+	-	-	-	-	NA	NA	hypothetical protein
AFR73195.1	+	-	+	-	+	+	-	+	+	-	-	-	+	+	COG1011	H	putative haloacid dehalogenase-like hydrolase
SaureusN305_ 1983	-	-	-	-	-	-	-	-	+	-	-	-	-	-	NA	NA	hypothetical protein
SaureusN305_ 1984	-	-	-	-	-	-	-	-	+	-	-	-	-	-	NA	NA	hypothetical protein
SaureusN305_ 1985	-	-	-	-	-	-	-	-	+	-	-	-	-	-	NA	NA	hypothetical protein
SaureusN305_ 1986	-	-	-	-	-	-	-	-	+	-	-	-	-	-	NA	NA	hypothetical protein
SaureusN305_ 1987	-	-	-	-	-	-	-	-	+	-	-	-	-	-	NA	NA	positive control sigma-like factor
SaureusN305_ 1988	-	-	-	-	-	-	-	-	+	-	-	-	-	-	NA	NA	hypothetical protein
SaureusN305_ 1989	-	-	-	-	-	-	-	-	+	-	-	-	+	-	NA	NA	Phage terminase large subunit
SaureusN305_ 1991	-	-	-	-	-	-	-	-	+	-	-	-	-	-	NA	NA	Phage Mu protein F like protein
AFR73457.1	+	-	+	+	-	+	+	-	-	-	+	+	+	+	COG0207	F	thymidylate synthase
SaureusN305_ 1992	-	-	-	-	-	-	-	-	+	-	-	-	-	-	NA	NA	hypothetical protein
SaureusN305_ 1993	-	-	-	-	-	-	-	-	+	-	-	-	+	-	NA	NA	hypothetical protein
SaureusN305_ 1994	-	-	-	-	-	-	-	-	+	-	-	-	-	-	NA	NA	hypothetical protein
SaureusN305_ 1996	-	-	-	-	-	-	-	-	+	-	-	-	+	-	NA	NA	Phage gp6-like head-tail connector protein
SaureusN305_ 1997	-	-	-	-	-	-	-	-	+	-	-	-	+	-	NA	NA	hypothetical protein
SaureusN305_ 1998	-	-	-	-	-	-	-	-	+	-	-	-	+	-	NA	NA	hypothetical protein
SaureusN305_ 1999	-	-	-	-	-	-	-	-	+	-	-	-	+	-	NA	NA	hypothetical protein
SaureusN305_ 2000	-	-	-	-	-	-	-	-	+	-	-	-	+	-	NA	NA	Phage major tail protein 2
SaureusN305_ 2001	-	-	-	-	-	-	-	-	+	-	-	-	+	-	NA	NA	Phage protein
SaureusN305_ 2002	-	-	-	-	-	-	-	-	+	-	-	-	+	-	NA	NA	hypothetical protein
SaureusN305_ 2004	-	-	-	-	-	-	-	-	+	-	-	-	+	-	NA	NA	Phage tail protein
SaureusN305_ 2005	-	-	-	-	-	-	-	-	+	-	-	-	-	-	NA	NA	Prophage endopeptidase tail

SaureusN305_ 2006	-	-	-	-	-	-	-	-	+	-	-	-	-	-	NA	NA	hypothetical protein
SaureusN305_ 2007	-	-	-	-	-	-	-	-	+	-	-	-	-	-	NA	NA	hypothetical protein
SaureusN305_ 2008	-	-	-	-	-	-	-	-	+	-	-	-	-	-	NA	NA	Holin family protein
SaureusN305_ 2009	-	-	-	-	-	-	-	-	+	-	-	-	-	-	NA	NA	N-acetylmuramoyl-L-alanine amidase CwlA precursor
SaureusN305_ 2140	-	-	-	-	-	-	-	-	+	-	-	-	-	-	NA	NA	Extracellular matrix-binding protein EbhA
AFR73461.1	+	-	+	+	-	-	+	+	-	-	-	-	+	-	NA	NA	hypothetical protein C248_1471
SaureusN305_ 2292	-	-	-	-	-	-	-	-	+	-	-	-	+	-	NA	NA	putative MFS-type transporter EfpA
SaureusN305_ 2293	-	-	-	-	-	-	-	-	+	-	-	-	+	-	NA	NA	hypothetical protein
SaureusN305_ 2295	-	-	-	-	-	-	-	-	+	-	-	-	-	-	NA	NA	hypothetical protein
SaureusN305_ 2299	-	-	-	-	-	-	-	-	+	-	-	-	-	-	COG0614	P	Iron(3+)-hydroxamate-binding protein FhuD precursor
SaureusN305_ 2300	-	-	-	-	-	-	-	-	+	-	-	-	-	-	COG3315	Q	Leucine carboxyl methyltransferase
SaureusN305_ 2303	-	-	-	-	-	-	-	-	+	-	+	-	-	-	NA	NA	putative leukocidin-like protein 1 precursor
SaureusN305_ 2309	-	-	-	-	-	-	-	-	+	-	+	-	-	+	COG1131	V	ABC transporter ATP-binding protein YtrB
SaureusN305_ 2310	-	-	-	-	-	-	-	-	+	-	+	-	-	+	NA	NA	hypothetical protein
SaureusN305_ 2312	-	-	-	-	-	-	-	-	+	-	+	-	-	+	NA	NA	ABC-2 family transporter protein
SaureusN305_ 2313	-	-	-	-	-	-	-	-	+	-	+	-	-	+	COG0526	O	hypothetical protein
SaureusN305_ 2314	-	-	-	-	-	-	-	-	+	-	+	-	-	+	NA	NA	hypothetical protein
SaureusN305_ 2315	-	-	-	-	-	-	-	-	+	-	+	-	-	+	NA	NA	hypothetical protein
SaureusN305_ 2316	-	-	-	-	-	-	-	-	+	-	+	-	-	-	NA	NA	hypothetical protein
SaureusN305_ 2317	-	-	-	-	-	-	-	-	+	-	+	-	-	-	COG4927	R	Acyl-coenzyme A:6-aminopenicillanic acid acyl-transferase
SaureusN305_ 2318	-	-	-	-	-	-	-	-	+	-	+	-	-	-	NA	NA	hypothetical protein
SaureusN305_ 2319	-	-	-	-	-	-	-	-	+	-	+	-	-	-	NA	NA	YolD-like protein
SaureusN305_ 2320	-	-	-	-	-	-	-	-	+	-	+	-	-	-	COG2706	G	6-phosphogluconolactonase
SaureusN305_ 2321	-	-	-	-	-	-	-	-	+	-	+	-	-	-	COG1012	C	Aldehyde dehydrogenase
SaureusN305_ 2322	-	-	-	-	-	-	-	-	+	-	+	-	-	-	COG1227	CP	putative manganese-dependent inorganic pyrophosphatase
SaureusN305_ 2323	-	-	-	-	-	-	-	-	+	-	+	-	-	-	COG1335	HR	Peroxyureidoacrylate/ureidoacrylate amidohydrolase RutB
SaureusN305_ 2324	-	-	-	-	-	-	-	-	+	-	+	-	-	-	NA	NA	Plasmin and fibronectin-binding protein A precursor

SaureusN305_2325	-	-	-	-	-	-	-	-	+	-	+	-	-	-	COG0471	G	Sodium-dependent dicarboxylate transporter SdcS
SaureusN305_2326	-	-	-	-	-	-	-	-	+	-	+	-	-	-	COG0077	E	P-protein
SaureusN305_2327	-	-	-	-	-	-	-	-	+	-	+	-	-	-	COG4362	P	Nitric oxide synthase oxygenase
SaureusN305_2328	-	-	-	-	-	-	-	-	+	-	+	-	-	-	COG1488	H	Nicotinate phosphoribosyltransferase pncB2
SaureusN305_2355	-	-	-	-	-	-	-	-	+	-	+	-	-	-	NA	NA	hypothetical protein
SaureusN305_2356	-	-	-	-	-	-	-	-	+	-	+	-	-	-	COG4758	S	hypothetical protein
SaureusN305_2357	-	-	-	-	-	-	-	-	+	-	+	-	-	-	COG4585	T	Sensor protein VraS
SaureusN305_2359	-	-	-	-	-	-	-	-	+	-	+	-	-	-	COG1295	S	hypothetical protein
SaureusN305_2360	-	-	-	-	-	-	-	-	+	-	+	-	-	-	NA	NA	hypothetical protein
SaureusN305_2361	-	-	-	-	-	-	-	-	+	-	+	+	-	-	COG0394	T	Low molecular weight protein-tyrosine-phosphatase PtpA
AFR72053.1	+	-	-	-	-	-	-	-	-	-	-	-	-	+	NA	NA	hypothetical protein C248_0039
SaureusN305_2362	-	-	-	-	-	-	-	-	+	-	+	+	-	-	NA	NA	hypothetical protein
SaureusN305_2363	-	-	-	-	-	-	-	-	+	-	+	+	-	-	COG2309	E	Aminopeptidase PepS
SaureusN305_2364	-	-	-	-	-	-	-	-	+	-	+	+	-	-	COG1607	I	putative acyl-CoA thioester hydrolase
SaureusN305_2365	-	-	-	-	-	-	-	-	+	-	+	+	-	-	COG0535	R	7-carboxy-7-deazaguanine synthase
SaureusN305_2366	-	-	-	-	-	-	-	-	+	-	-	-	-	-	NA	NA	hypothetical protein
SaureusN305_2367	-	-	-	-	-	-	-	-	+	-	-	-	-	-	COG0693	R	Putative cysteine protease YraA
SaureusN305_2368	-	-	-	-	-	-	-	-	+	-	-	-	-	-	COG0744	M	Monofunctional glycosyltransferase
SaureusN305_2369	-	-	-	-	-	-	-	-	+	-	-	-	-	-	COG2137	O	Regulatory protein RecX
SaureusN305_2370	-	-	-	-	-	-	-	-	+	-	-	-	-	-	NA	NA	hypothetical protein
SaureusN305_2371	-	-	-	-	-	-	-	-	+	-	-	-	-	-	COG1134	GM	Teichoic acids export ATP-binding protein TagH
SaureusN305_2431	-	-	-	-	-	-	-	-	+	-	-	-	-	-	NA	NA	Putative surface protein precursor
SaureusN305_2440	-	-	-	-	-	-	-	-	+	+	-	-	-	-	NA	NA	putative lipoprotein precursor
SaureusN305_2442	-	-	-	-	-	-	-	-	+	-	-	-	-	-	NA	NA	hypothetical protein
SaureusN305_2444	-	-	-	-	-	-	-	-	+	+	-	-	-	-	NA	NA	hypothetical protein
SaureusN305_2445	-	-	-	-	-	-	-	-	+	+	-	-	-	-	COG4221	C	putative oxidoreductase
AFR72054.1	+	-	-	-	-	-	-	-	-	-	-	-	-	+	NA	NA	hypothetical protein C248_0040
AFR73801.1	+	-	+	-	-	-	+	-	-	-	-	-	-	+	NA	NA	Hypothetical Protein C248_1811

SaureusNZMR 0322_01447	-	-	-	-	-	-	-	-	+	-	-	-	-	-	NA	NA	chromosome segregation protein
SaureusNZMR 0322_01754	-	-	-	-	-	-	-	-	+	-	-	-	-	-	NA	NA	ribulose-1,5-biphosphate synthetase
AFR73803.1	+	-	+	-	-	+	-	-	-	-	-	-	-	-	COG0054	H	6,7-dimethyl-8-ribityllumazine synthase
AFR73835.1	+	-	+	-	+	+	-	-	-	-	-	-	+	+	NA	NA	putative lipoprotein
SaureusNZMR 0322_01857	-	-	-	-	-	-	-	-	+	-	-	-	-	-	NA	NA	hypothetical protein
AFR73836.1	+	-	+	-	+	+	-	-	-	-	-	-	+	+	NA	NA	hypothetical protein C248_1846
SaureusNZMR 0322_01897	-	-	-	-	-	-	-	-	+	-	-	-	-	-	NA	NA	hypothetical protein
SaureusNZMR 0322_02007	-	-	-	-	-	-	-	-	+	-	+	-	-	-	COG0533	J	tRNA N6-adenosine threonylcarbamoyltransferase
SaureusNZMR 0322_02008	-	-	-	-	-	-	-	-	+	-	+	-	-	-	COG0456	J	ribosomal-protein-alanine N-acetyltransferase
SaureusNZMR 0322_02009	-	-	-	-	-	-	-	-	+	-	+	-	-	-	COG1214	J	tRNA threonylcarbamoyladenosine biosynthesis protein TsaB
SaureusNZMR 0322_02010	-	-	-	-	-	-	-	-	+	-	+	-	-	-	COG0802	J	tRNA threonylcarbamoyladenosine biosynthesis protein TsaE
AFR73837.1	+	-	+	-	+	+	-	-	-	-	-	-	+	+	NA	NA	hypothetical protein C248_1847
SaureusNZMR 0322_02011	-	-	-	-	-	-	-	-	+	-	+	-	-	-	COG0129	EG	Dihydroxy-acid dehydratase
SaureusNZMR 0322_02020	-	-	-	-	-	-	-	-	+	-	-	-	-	-	NA	NA	Cell wall-associated hydrolase
AFR73838.1	+	-	+	-	-	-	-	-	-	-	-	-	-	+	NA	NA	hypothetical protein C248_1848
AFR73839.1	+	-	+	-	-	-	-	-	-	-	+	-	-	+	NA	NA	hypothetical protein C248_1849
SaureusNZMR 0322_02123	-	-	-	-	-	-	-	-	+	-	-	-	-	-	NA	NA	Transposon Tn7 transposition protein TnsC
SaureusNZMR 0322_02124	-	-	-	-	-	-	-	-	+	-	-	-	-	-	COG3464	X	Transposase
SaureusNZMR 0322_02125	-	-	-	-	-	-	-	-	+	-	-	-	-	-	COG3666	X	Transposase DDE domain protein
SaureusNZMR 0322_02126	-	-	-	-	-	-	-	-	+	-	-	-	-	-	COG3666	X	hypothetical protein
AFR73840.1	+	-	+	-	-	-	-	-	-	-	-	-	-	+	NA	NA	hypothetical protein C248_1850
AFR72055.1	+	-	-	-	-	-	-	-	-	-	-	-	-	-	NA	NA	hypothetical protein C248_0041
AFR73841.1	+	-	+	-	-	-	-	-	-	-	-	-	-	+	NA	NA	Transposase
AFR73843.1	+	-	+	-	-	-	-	-	-	-	-	-	-	+	NA	NA	Hypothetical protein C248_1853
SaureusNZMR 0322_02186	-	-	-	-	-	-	-	-	+	-	-	-	-	-	NA	NA	hypothetical protein
SaureusNZMR 0322_02187	-	-	-	-	-	-	-	-	+	-	-	-	-	-	NA	NA	hypothetical protein
SaureusNZMR 0322_02188	-	-	-	-	-	-	-	-	-	+	-	-	-	+	NA	NA	YopX protein
AFR73844.1	+	-	+	-	-	+	-	-	+	-	-	-	+	+	NA	NA	hypothetical protein C248_1854
AFR73845.1	+	-	+	-	-	-	-	+	+	-	+	-	+	+	NA	NA	putative exported protein
SaureusNZMR 0322_02211	-	-	-	-	-	-	-	-	+	-	-	-	-	-	NA	NA	hypothetical protein
SaureusNZMR 0322_02212	-	-	-	-	-	-	-	-	+	-	-	-	-	-	NA	NA	hypothetical protein
AFR73846.1	+	-	+	-	-	-	-	+	+	-	-	-	+	+	NA	NA	hyaluronate lyase precursor 1
AFR73847.1	+	-	+	-	+	+	-	+	+	+	-	-	+	+	NA	NA	putative exported protein
SaureusNZMR	-	-	-	-	-	-	-	-	+	-	-	-	-	-	COG1251	C	Nitrite reductase [NAD(P)H]

0322_02438	-	-	-	-	-	-	-	-	+	-	-	-	-	-	COG1251	C	Nitrite reductase [NAD(P)H]
SaureusNZMR 0322_02439	-	-	-	-	-	-	-	-	+	-	-	-	-	-	COG1251	C	Nitrite reductase [NAD(P)H]
AFR73848.1	+	-	+	-	-	-	-	-	+	-	-	-	-	+	NA	NA	putative exported protein
SaureusNZMR 0322_02445	-	-	-	-	-	-	-	-	+	-	-	-	-	-	COG0803	P	Metal-binding protein ZinT precursor
SaureusNZMR 0322_02446	-	-	-	-	-	-	-	-	+	-	-	-	-	-	NA	NA	High-affinity zinc uptake system binding-protein ZnuA
SaureusNZMR 0322_02449	-	-	-	-	-	-	-	-	+	-	-	-	-	-	COG2348	M	Aminoacyltransferase FemA
SaureusNZMR 0322_02522	-	-	-	-	-	-	-	-	+	-	-	-	-	-	COG2963	X	Transposase
SaureusNZMR 0322_02523	-	-	-	-	-	-	-	-	+	-	-	-	-	-	NA	NA	hypothetical protein
AFR73849.1	+	-	-	-	-	-	-	-	-	-	-	-	-	+	NA	NA	hypothetical protein C248_1859
AFR72056.1	+	-	-	-	-	-	-	-	-	-	-	-	-	+	NA	NA	hypothetical protein C248_0042
AFR72029.1	+	-	+	-	-	-	-	-	-	-	-	-	+	-	COG4241	S	putative membrane protein
SaureusNZMR 0322_02541	-	-	-	-	-	-	-	-	+	-	-	-	-	-	NA	NA	Fibronectin-binding protein A precursor
SaureusNZMR 0322_02542	-	-	-	-	-	-	-	-	+	-	-	-	-	-	NA	NA	Fibronectin-binding protein A precursor
AFR73851.1	+	-	-	+	-	-	-	-	-	-	-	-	-	+	COG4584	X	Putative transposase
AFR73852.1	+	-	+	+	-	-	-	-	-	+	-	-	-	+	NA	NA	putative lipoprotein
ATV02856.1	-	-	-	-	-	-	-	-	-	-	+	-	-	-	COG3591	E	Serine protease
AFR73853.1	+	-	-	-	+	+	+	+	-	-	+	-	+	+	COG3545	R	hypothetical protein C248_1871
ATV02866.1	-	-	-	-	-	-	-	-	-	-	+	-	-	-	NA	NA	hypothetical protein SaO11_00040
ATV02868.1	-	-	-	-	-	-	-	-	-	-	+	+	-	-	NA	NA	hypothetical protein SaO11_00042
ATV02869.1	-	-	-	-	-	-	-	-	-	-	+	+	-	-	NA	NA	DNA helicase protein
ATV02874.1	-	-	-	-	-	-	-	-	-	-	+	+	-	-	NA	NA	BioC protein
AFR74016.1	+	-	-	+	-	-	+	-	-	+	+	+	-	+	COG0059	EH	ketol-acid reductoisomerase
ATV02876.1	-	-	-	-	-	-	-	-	-	-	+	+	-	-	COG3208	Q	Iron aquisition yersiniabactin synthesis enzyme (YbtT,resembles
ATV02877.1	-	-	-	-	-	-	-	-	-	-	+	+	-	-	NA	NA	4'-phosphopantetheinyl transferase
ATV02878.1	-	-	-	-	-	-	-	-	-	-	+	+	-	-	NA	NA	2,3-dihydroxybenzoate-AMP ligase
ATV02879.1	-	-	-	-	-	-	-	-	-	-	+	+	-	-	NA	NA	Non-ribosomal peptide synthetase
ATV02880.1	-	-	-	-	-	-	-	-	-	-	+	+	-	-	NA	NA	Polyketide synthase
ATV02881.1	-	-	-	-	-	-	-	-	-	-	+	+	-	-	NA	NA	hypothetical protein SaO11_00055
ATV02882.1	-	-	-	-	-	-	-	-	-	-	+	+	-	-	COG1020	Q	Iron aquisition yersiniabactin synthesis enzyme (Irp2)
ATV02883.1	-	-	-	-	-	-	-	-	-	-	+	-	-	-	NA	NA	Substrate-specific component of ECF transporter
ATV02884.1	-	-	-	-	-	-	-	-	-	-	+	-	-	-	COG0619	H	Transmembrane component of energizing module of
ATV02885.1	-	-	-	-	-	-	-	-	-	-	+	-	-	-	COG1122	PR	Duplicated ATPase component of energizing module
AFR74017.1	+	-	-	+	-	-	+	-	-	+	+	+	-	+	COG0119	E	2-isopropylmalate synthase
AFR74018.1	+	-	-	+	-	-	+	-	-	+	+	+	-	+	COG0473	CE	3-isopropylmalate dehydrogenase
ATV03138.1	-	-	-	-	-	-	-	-	-	+	+	-	-	-	NA	NA	Phage tail protein
AFR74019.1	+	-	-	+	-	-	+	-	-	+	+	+	-	+	COG0065	E	3-isopropylmalate dehydratase large subunit
AFR74020.1	+	-	-	+	-	-	+	-	-	+	+	+	-	+	COG0066	E	3-isopropylmalate dehydratase small subunit
ATV03255.1	-	-	-	-	-	-	-	-	-	-	+	-	-	-	NA	NA	hypothetical protein SaO11_00429
AFR74021.1	+	-	-	+	-	-	+	-	-	+	+	+	-	+	COG1171	E	threonine dehydratase biosynthetic
ATV03264.1	-	-	-	-	-	-	-	-	-	-	+	-	-	-	NA	NA	hypothetical protein SaO11_00438
ATV03271.1	-	-	-	-	-	-	-	-	-	-	+	-	-	-	COG1388	M	N-acetylmuramoyl-L-alanine amidase sle1
ATV03272.1	-	-	-	-	-	-	-	-	-	-	+	-	-	-	NA	NA	hypothetical protein SaO11_00446
ATV03273.1	-	-	-	-	-	-	-	-	-	-	+	-	+	-	COG0494	V	MutT domain containing protein
AFR74022.1	+	-	-	+	-	-	+	-	-	+	+	+	-	+	COG3091	R	hypothetical protein C248_2073

AFR72057.1	+	-	-	-	-	-	-	-	-	-	-	-	-	+	NA	NA	hypothetical protein C248_0043
ATV03361.1	-	-	-	-	-	-	-	-	-	-	+	-	-	-	NA	NA	Putative deoxyadenosine kinase protein
AFR74023.1	+	-	-	+	-	-	-	-	-	+	+	+	-	+	COG2183	K	putative RNA binding protein
ATV04437.1	-	-	-	-	-	-	-	-	-	-	+	+	-	-	NA	NA	Hypothetical protein SaO11_01611
ATV04473.1	-	-	-	-	-	-	-	-	-	-	+	-	-	-	NA	NA	hypothetical protein SaO11_01647
ATV04475.1	-	-	-	-	-	-	-	-	-	-	+	-	-	-	NA	NA	Putative membrane protein
ATV04477.1	-	-	-	-	-	-	-	-	-	-	+	-	-	-	NA	NA	NTPase
ATV04535.1	-	-	-	-	-	-	-	-	-	-	+	-	-	-	NA	NA	Phage protein
ATV04562.1	-	-	-	-	-	-	-	-	-	-	+	-	-	-	NA	NA	Phage protein
ATV04567.1	-	-	-	-	-	-	-	-	-	-	+	-	-	-	NA	NA	hypothetical protein SaO11_01741
ATV04572.1	-	-	-	-	-	-	-	-	-	-	+	-	-	-	NA	NA	hypothetical protein SaO11_01746
AFR72058.1	+	-	-	-	-	-	-	-	-	-	-	-	-	+	NA	NA	Hypothetical protein C248_0044
ATV04584.1	-	-	-	-	-	-	-	-	-	-	+	+	-	-	NA	NA	hypothetical protein SaO11_01758
AFR74376.1	+	-	+	+	-	-	-	-	-	+	-	-	-	+	COG5504	S	hypothetical protein C248_2436
ATV04604.1	-	-	-	-	-	-	-	-	-	-	+	-	-	-	COG0024	J	Putative metallopeptidase
ATV04605.1	-	-	-	-	-	-	-	-	-	-	+	-	-	-	COG4129	S	Putative membrane protein
ATV04606.1	-	-	-	-	-	-	-	-	-	-	+	-	-	-	NA	NA	Hypothetical protein SaO11_01780
ATV04607.1	-	-	-	-	-	-	-	-	-	-	+	-	-	-	COG3442	R	Hypothetical protein SaO11_01781
ATV04608.1	-	-	-	-	-	-	-	-	-	-	+	-	-	-	COG0769	M	Mur ligase family protein
ATV04609.1	-	-	-	-	-	-	-	-	-	-	+	-	-	-	COG1528	P	Ferritin
ATV04610.1	-	-	-	-	-	-	-	-	-	-	+	-	-	-	COG0847	L	Putative exonuclease
ATV04611.1	-	-	-	-	-	-	-	-	-	-	+	-	-	-	COG0389	L	ImpB/MucB/SamB family protein
ATV04612.1	-	-	-	-	-	-	-	-	-	-	+	-	-	-	NA	NA	Putative membrane protein
ATV04613.1	-	-	-	-	-	-	-	-	-	-	+	-	-	-	COG2265	J	Putative RNA methyltransferase
ATV04614.1	-	-	-	-	-	-	-	-	-	-	+	-	-	-	COG1597	IR	Hypothetical protein SaO11_01788
ATV04615.1	-	-	-	-	-	-	-	-	-	-	+	-	-	-	NA	NA	hypothetical protein SaO11_01789
ATV04616.1	-	-	-	-	-	-	-	-	-	-	+	-	-	-	COG0064	J	Glutamyl-tRNA amidotransferase subunit B
ATV04617.1	-	-	-	-	-	-	-	-	-	-	+	-	-	-	COG0154	J	Glutamyl-tRNA amidotransferase subunit A
ATV04618.1	-	-	-	-	-	-	-	-	-	-	+	-	-	-	COG0721	J	Aspartyl/glutamyl-tRNA(Asn/Gln) amidotransferase subunit C
ATV04619.1	-	-	-	-	-	-	-	-	-	-	+	-	-	-	COG0591	E	High affinity proline permease
ATV04620.1	-	-	-	-	-	-	-	-	-	-	+	-	-	-	COG1733	K	Transcriptional regulator
ATV04621.1	-	-	-	-	-	-	-	-	-	-	+	-	-	-	COG2070	R	Nitronate monooxygenase
ATV04622.1	-	-	-	-	-	-	-	-	-	-	+	-	-	-	COG4851	R	Putative lipoprotein
ATV04623.1	-	-	-	-	-	-	-	-	-	-	+	-	-	-	COG0272	L	DNA ligase
ATV04624.1	-	-	-	-	-	-	-	-	-	-	+	-	-	-	COG0210	L	ATP-dependent DNA helicase
ATV04625.1	-	-	-	-	-	-	-	-	-	-	+	-	-	-	COG1646	I	PcrB family protein
ATV04626.1	-	-	-	-	-	-	-	-	-	-	+	-	-	-	COG4496	S	hypothetical protein SaO11_01800
ATV04627.1	-	-	-	-	-	-	-	-	-	-	+	-	-	-	COG0015	F	Adenylosuccinate lyase
ATV04628.1	-	-	-	-	-	-	-	-	-	-	+	-	-	-	NA	NA	Staphopain A
ATV04629.1	-	-	-	-	-	-	-	-	-	-	+	-	-	-	COG4843	S	hypothetical protein SaO11_01803
ATV04630.1	-	-	-	-	-	-	-	-	-	-	+	-	-	-	COG0171	H	NAD(+) synthetase
ATV04638.1	-	-	-	-	-	-	-	-	-	-	+	-	-	-	NA	NA	hypothetical protein SaO11_01812
ATV04652.1	-	-	-	-	-	-	-	-	-	-	+	-	-	+	COG1725	K	GntR family transcriptional regulator
ATV04653.1	-	-	-	-	-	-	-	-	-	-	+	-	-	+	NA	NA	hypothetical protein SaO11_01827
ATV04654.1	-	-	-	-	-	-	-	-	-	-	+	-	-	+	COG0436	E	Hypothetical protein SaO11_01828
ATV04655.1	-	-	-	-	-	-	-	-	-	-	+	-	-	-	NA	NA	Extracellular adherence protein Eap/Map
ATV04656.1	-	-	-	-	-	-	-	-	-	-	+	-	-	-	NA	NA	Extracellular adherence protein of broad specificity
ATV04661.1	-	-	-	-	-	-	-	-	-	-	+	-	-	-	NA	NA	hypothetical protein SaO11_01835
ATV04666.1	-	-	-	-	-	-	-	-	-	-	+	-	-	-	COG0234	O	10 kDa chaperonin
ATV04667.1	-	-	-	-	-	-	-	-	-	-	+	-	-	-	COG1266	O	Putative membrane protein
ATV04668.1	-	-	-	-	-	-	-	-	-	-	+	-	-	-	NA	NA	Serine-aspartate repeat family protein, SdrH

ATV04669.1	-	-	-	-	-	-	-	-	-	-	+	-	-	-	COG3560	R	Hypothetical protein SaO11_01843
ATV04670.1	-	-	-	-	-	-	-	-	-	-	+	-	-	-	COG0388	R	Putative carbon-nitrogen hydrolase
ATV04671.1	-	-	-	-	-	-	-	-	-	-	+	-	-	-	NA	NA	Delta-hemolysin
ATV04672.1	-	-	-	-	-	-	-	-	-	-	+	-	-	-	NA	NA	Accessory regulator protein B
ATV04673.1	-	-	-	-	-	-	-	-	-	-	+	-	-	-	NA	NA	AgrD protein
ATV04674.1	-	-	-	-	-	-	-	-	-	-	+	-	-	-	NA	NA	Autoinducer sensor AgrC
ATV04675.1	-	-	-	-	-	-	-	-	-	-	+	-	-	-	COG3279	KT	DNA-binding response regulator
ATV04676.1	-	-	-	-	-	-	-	-	-	-	+	-	-	-	COG0524	G	hypothetical protein SaO11_01850
ATV04677.1	-	-	-	-	-	-	-	-	-	-	+	-	-	-	COG1621	G	Invertase
ATV04678.1	-	-	-	-	-	-	-	-	-	-	+	-	-	-	COG1609	K	Sucrose operon repressor
AFR72059.1	+	-	-	-	-	-	-	-	-	-	-	-	-	+	NA	NA	hypothetical protein C248_0045
ATV04679.1	-	-	-	-	-	-	-	-	-	-	+	-	-	-	COG0004	P	Ammonium transporter family protein
ATV04680.1	-	-	-	-	-	-	-	-	-	-	+	-	-	-	COG0425	O	SirA family protein
ATV04681.1	-	-	-	-	-	-	-	-	-	-	+	-	-	-	COG2391	R	Membrane protein
ATV04682.1	-	-	-	-	-	-	-	-	-	-	+	-	-	-	COG2344	K	Hypothetical protein SaO11_01856
ATV04683.1	-	-	-	-	-	-	-	-	-	-	+	-	-	-	NA	NA	ABC transporter ATP-binding protein
ATV04684.1	-	-	-	-	-	-	-	-	-	-	+	-	-	-	COG0249	L	Putative membrane protein
ATV04691.1	-	-	-	-	-	-	-	-	-	-	+	+	-	-	NA	NA	Acetolactate synthase 1 regulatory subunit
ATV04704.1	-	-	-	-	-	-	-	-	-	-	+	+	-	-	COG2337	V	Endoribonuclease MazF
ATV05031.1	-	-	-	-	-	-	-	-	-	-	+	-	-	-	NA	NA	Addiction module antitoxin
AUG72740.1	-	-	-	-	-	-	-	-	-	-	-	+	-	-	NA	NA	hypothetical protein SAO46_00265
AUG72752.1	-	-	-	-	-	-	-	-	-	-	-	+	-	-	NA	NA	Nuclease
AFR74393.1	+	-	-	-	+	+	+	+	-	-	+	+	+	-	COG0803	P	putative solute binding lipoprotein
AUG72754.1	-	-	-	-	-	-	-	-	-	-	-	+	-	-	NA	NA	hypothetical protein SAO46_00280
AUG72755.1	-	-	-	-	-	-	-	-	-	-	-	+	-	+	NA	NA	PVL ORF-50-like family protein
AFR74394.1	+	-	-	-	+	-	+	-	-	+	+	-	-	-	COG1651	O	putative lipoprotein
AFR72060.1	+	-	-	-	-	+	-	-	-	-	-	-	-	+	COG1961	L	Recombinase CcrC
AUG72767.1	-	-	-	-	-	-	-	-	-	-	-	+	-	+	NA	NA	hypothetical protein SAO46_00293
AFR74395.1	+	-	-	-	+	-	+	-	-	+	-	-	+	+	NA	NA	putative lipoprotein
AFR74488.1															COG1109	G	putative phosphomannomutase
AUG72787.1	-	-	-	-	-	-	-	-	-	-	-	+	-	-	NA	NA	Phage protein
AUG72790.1	-	-	-	-	-	-	-	-	-	-	-	+	-	-	NA	NA	Phage-like protein
AUG72791.1	-	-	-	-	-	-	-	-	-	-	-	+	-	-	NA	NA	XkdX family protein
AFR74489.1	+	-	+	+	+	-	+	+	+	+	-	+	+	-	NA	NA	putative exported protein
AFR74490.1	+	-	+	+	+	-	+	+	+	+	-	+	+	+	NA	NA	putative membrane protein
AFR74491.1	+	-	+	+	+	+	+	+	+	+	-	+	+	+	COG1210	M	UTP--glucose-1-phosphate uridylyltransferase
AFR74492.1	+	-	-	-	-	-	-	-	-	+	-	-	-	+	NA	NA	Fibronectin-binding protein A
AUG74185.1	-	-	-	-	-	-	-	-	-	-	-	+	-	-	NA	NA	Nitrogen regulation protein NIFR3
AFR74494.1	+	-	-	-	-	-	-	-	-	-	-	-	-	+	NA	NA	Fibronectin binding protein precursor
AFR74493.1	+	-	-	-	-	-	-	-	-	-	-	-	-	+	NA	NA	Hypothetical protein C248_2553
AFR74495.1	+	-	+	+	+	+	+	+	+	+	-	+	+	+	NA	NA	Fibronectin-binding protein A
AUG74243.1	-	-	-	-	-	-	-	-	-	-	-	+	-	-	NA	NA	Phage protein
AFR74496.1	+	-	-	-	-	-	-	-	-	-	-	-	-	+	NA	NA	Hypothetical protein C248_2556
AFR72061.1	+	-	-	-	-	-	-	-	-	-	-	-	-	+	NA	NA	Hypothetical protein C248_0047
AUG74246.1	-	-	-	-	-	-	-	-	-	-	-	+	-	-	NA	NA	hypothetical protein SAO46_01835
AUG74249.1	-	-	-	-	-	-	-	-	-	-	-	+	-	-	NA	NA	hypothetical protein SAO46_01838
AUG74250.1	-	-	-	-	-	-	-	-	-	-	-	+	-	-	COG0305	L	DNA helicase, phage-associated
AFR74497.1	+	-	+	+	+	+	-	+	+	+	-	+	+	+	COG2610	GR	putative gluconate permease
AUG74263.1	-	-	-	-	-	-	-	-	-	-	-	+	-	-	NA	NA	hypothetical protein SAO46_01852
AFR74498.1	+	-	+	+	+	+	-	+	+	+	-	+	+	+	COG1070	G	putative gluconokinase
AUG74264.1	-	-	-	-	-	-	-	-	-	-	-	+	-	-	NA	NA	hypothetical protein SAO46_01853

AFR74499.1	+	-	+	+	-	+	-	+	+	+	-	+	+	+	COG1802	K	gluconate operon transcriptional repressor
AFR74500.1	+	-	+	+	-	+	-	-	+	+	-	+	+	+	COG0789	K	MerR family regulatory protein
AFR74501.1	+	-	+	+	-	+	-	-	+	+	-	+	+	+	COG2357	FT	hypothetical protein C248_2561
AFR74502.1	+	-	+	+	-	-	-	-	+	+	-	+	+	+	COG4876	S	hypothetical protein C248_2562
AFR74503.1	+	-	+	+	-	-	-	-	+	+	-	+	+	+	COG1289	S	putative membrane protein
AFR72480.1	+	+	+	-	+	-	-	+	+	-	-	-	+	+	COG2963	X	Transposase
AFR72481.1	+	+	+	-	+	-	-	+	+	-	-	-	-	-	NA	NA	hypothetical protein C248_0468
AFR72482.1	+	+	+	-	+	-	-	+	+	-	-	-	-	-	COG3759	S	Hypothetical Protein C248_0469
AFR72062.1	+	-	-	-	-	-	-	-	-	-	-	-	-	+	NA	NA	hypothetical protein C248_0048
CAI79713.1	-	-	-	-	-	-	-	-	-	-	-	-	+	-	NA	NA	conserved hypothetical protein
CAI79714.1	-	-	-	-	-	-	-	-	-	-	-	-	+	-	NA	NA	probable enterotoxin protein
AFR72483.1	+	+	+	-	+	-	-	+	+	-	-	-	-	-	COG0702	R	hypothetical protein C248_0470
CAI79911.1	-	-	-	-	-	-	-	-	-	-	-	-	+	-	COG4842	S	conserved hypothetical protein
CAI79912.1	-	-	-	-	-	-	-	-	-	-	-	-	+	-	COG1511	S	probable membrane protein
CAI79914.1	-	-	-	-	-	-	-	-	-	-	-	-	+	-	COG5417	S	conserved hypothetical protein
CAI79915.1	-	-	-	-	-	-	-	-	-	-	-	-	+	-	COG4499	S	probable membrane protein
CAI79916.1	-	-	-	-	-	-	-	-	-	-	-	-	+	-	COG1674	D	probable DNA segregation ATPase and related
CAI79917.1	-	-	-	-	-	-	-	-	-	-	-	-	+	-	NA	NA	hypothetical protein SAB0229
CAI79918.1	-	-	-	-	-	-	-	-	-	-	-	-	+	-	NA	NA	hypothetical protein SAB0230
CAI79919.1	-	-	-	-	-	-	-	-	-	-	-	-	+	-	NA	NA	hypothetical protein SAB0231
CAI79920.1	-	-	-	-	-	-	-	-	-	-	-	-	+	-	NA	NA	hypothetical protein SAB0232
AFR72485.1	+	+	+	-	+	-	-	+	+	-	-	-	-	-	NA	NA	superantigen-like protein
CAI79922.1	-	-	-	-	-	-	-	-	-	-	-	-	+	-	NA	NA	conserved hypothetical protein
CAI79923.1	-	-	-	-	-	-	-	-	-	-	-	-	+	-	NA	NA	hypothetical protein SAB0235
CAI79924.1	-	-	-	-	-	-	-	-	-	-	-	-	+	-	NA	NA	truncated hypothetical protein
CAI79929.1	-	-	-	-	-	-	-	-	-	-	-	-	+	-	NA	NA	probable membrane protein
CAI79930.1	-	-	-	-	-	-	-	-	-	-	-	-	+	-	COG2116	P	probable formate/nitrite transport protein
CAI79931.1	-	-	-	-	-	-	-	-	-	-	-	-	+	-	COG1114	E	probable branched-chain amino acid transport system
AFR72486.1	+	+	+	-	+	-	-	+	+	-	-	-	-	-	NA	NA	exotoxin
CAI80035.1	-	-	-	-	-	-	-	-	-	-	-	-	+	-	NA	NA	hypothetical bovine pathogenicity island protein
CAI80036.1	-	-	-	-	-	-	-	-	-	-	-	-	+	-	NA	NA	bovine pathogenicity island protein Orf17
AFR72487.1	+	+	+	-	+	-	-	+	+	-	-	-	-	-	NA	NA	exotoxin
CAI80042.1	-	-	-	-	-	-	-	-	-	-	-	-	+	-	NA	NA	bovine pathogenicity island protein Orf10
AFR72488.1	+	+	+	-	+	-	-	+	+	-	-	-	-	-	NA	NA	Exotoxin
CAI80078.1	-	-	-	-	-	-	-	-	-	-	-	-	+	-	NA	NA	probable lipoprotein
CAI80079.1	-	-	-	-	-	-	-	-	-	-	-	-	+	-	NA	NA	hypothetical protein SAB0391
AFR72490.1	+	+	+	-	+	-	-	+	-	-	-	-	-	-	NA	NA	superantigen-like protein
AFR72492.1	+	+	+	-	+	-	-	+	-	-	-	-	-	-	NA	NA	exotoxin 1
AFR72493.1	+	+	+	-	+	-	-	+	-	-	-	-	-	-	NA	NA	exotoxin 5
CAI80428.1															NA	NA	hypothetical protein SAB0740
AFR72494.1	+	+	+	-	+	+	-	+	-	-	-	-	+	-	NA	NA	exotoxin 4
CAI80432.1															NA	NA	truncated clumping factor
AFR72495.1	+	+	+	-	+	+	-	+	+	-	-	-	+	-	COG0286	V	putative type I restriction enzyme modification
AFR72063.1	+	-	-	-	-	-	-	-	-	-	-	-	-	+	NA	NA	hypothetical protein C248_0049
CAI80711.1	-	-	-	-	-	-	-	-	-	-	-	-	+	-	NA	NA	probable membrane protein
AFR72496.1	+	+	+	-	+	+	-	+	+	-	-	-	+	-	COG0732	V	Restriction modification system DNA specificity subunit
AFR72497.1	+	+	+	-	+	-	-	+	-	-	+	-	-	-	NA	NA	Exotoxin type C
CAI80726.1	-	-	-	-	-	-	-	-	-	-	-	-	+	-	NA	NA	hypothetical protein SAB1038
CAI80978.1	-	-	-	-	-	-	-	-	-	-	-	-	+	-	NA	NA	truncated cell surface fibronectin-binding protein
Saureus08S 00974_00415	-	+	+	-	+	-	-	+	-	+	+	-	+	+	NA	NA	hypothetical protein
CAI80979.1	-	-	-	-	-	-	-	-	-	-	-	-	+	-	NA	NA	truncated cell surface fibronectin-binding protein

CAI80980.1	-	-	-	-	-	-	-	-	-	-	-	-	+	-	NA	NA	truncated cell surface fibronectin-binding protein
CAI80981.1	-	-	-	-	-	-	-	-	-	-	-	-	+	-	NA	NA	truncated cell surface fibronectin-binding protein
CAI80982.1	-	-	-	-	-	-	-	-	-	-	-	-	+	-	NA	NA	truncated cell surface fibronectin-binding protein
CAI80984.1	-	-	-	-	-	-	-	-	-	-	-	-	+	-	NA	NA	hypothetical protein SAB1295
CAI80985.1	-	-	-	-	-	-	-	-	-	-	-	-	+	-	COG0604	CR	probable alcohol dehydrogenase
CAI80986.1	-	-	-	-	-	-	-	-	-	-	-	-	+	-	COG1846	K	conserved hypothetical protein
CAI80989.1	-	-	-	-	-	-	-	-	-	-	-	-	+	-	NA	NA	truncated cell surface fibronectin-binding protein
AFR72500.1	+	+	+	-	+	-	-	+	-	-	+	-	+	-	NA	NA	putative exported protein
AFR72501.1	+	+	+	-	+	-	-	+	-	-	-	-	+	-	NA	NA	putative lipoprotein
CAI81349.1	-	-	-	-	-	-	-	-	-	-	-	-	+	-	NA	NA	conserved hypothetical protein
CAI81350.1	-	-	-	-	-	-	-	-	-	-	-	-	+	-	NA	NA	conserved hypothetical protein
AFR72502.1	+	+	+	-	-	-	-	-	-	-	-	-	-	-	NA	NA	putative lipoprotein
CAI81359.1	-	-	-	-	-	-	-	-	-	-	-	-	+	-	COG3591	E	serine proteinase
CAI81363.1	-	-	-	-	-	-	-	-	-	-	-	-	+	-	NA	NA	hypothetical protein SAB1674c, partial
AFR72503.1	+	+	+	+	+	-	-	+	+	+	-	-	+	+	NA	NA	putative lipoprotein
AFR72504.1	+	+	+	-	+	+	-	+	+	+	+	-	+	-	NA	NA	putative lipoprotein
CAI81377.1	-	-	-	-	-	-	-	-	-	-	-	-	+	-	NA	NA	hypothetical protein SAB1688
CAI81378.1	-	-	-	-	-	-	-	-	-	-	-	-	+	-	NA	NA	hypothetical protein SAB1689c
CAI81381.1	-	-	-	-	-	-	-	-	-	-	-	-	+	-	NA	NA	hypothetical protein SAB1692
CAI81384.1	-	-	-	-	-	-	-	-	-	-	-	-	+	-	NA	NA	hypothetical protein SAB1695
CAI81385.1	-	-	-	-	-	-	-	-	-	-	-	-	+	-	NA	NA	enterotoxin G
AFR72505.1	+	+	+	-	-	-	-	-	-	-	-	-	-	-	NA	NA	putative lipoprotein
CAI81387.1	-	-	-	-	-	-	-	-	-	-	-	-	+	-	NA	NA	enterotoxin type C variant
CAI81391.1	-	-	-	-	-	-	-	-	-	-	-	-	+	-	NA	NA	hypothetical protein SAB1702c
CAI81394.1	-	-	-	-	-	-	-	-	-	-	-	-	+	-	NA	NA	phage-related tail fiber
AFR72506.1	+	+	+	-	-	-	-	-	-	-	-	-	-	-	NA	NA	putative lipoprotein
AFR72064.1	+	-	-	-	-	-	-	-	-	-	-	-	-	-	NA	NA	hypothetical protein C248_0050
AFR72507.1	+	+	-	-	-	-	-	-	-	-	-	-	-	-	NA	NA	putative lipoprotein
CAI81413.1	-	-	-	-	-	-	-	-	-	-	-	-	+	-	NA	NA	hypothetical phage-related protein
CAI81416.1	-	-	-	-	-	-	-	-	-	-	-	-	+	-	COG3728	X	hypothetical mobile-element-associated protein
AFR72508.1	+	+	-	-	-	-	-	-	-	-	-	-	-	-	NA	NA	putative lipoprotein
CAI81420.1	-	-	-	-	-	-	-	-	-	-	-	-	+	-	NA	NA	hypothetical phage-related protein
AFR72509.1	+	+	+	-	+	-	-	+	-	-	+	-	+	-	NA	NA	putative lipoprotein
AFR72510.1	+	+	+	-	-	-	-	+	-	+	+	-	+	-	NA	NA	hypothetical protein C248_0497
CAI81439.1	-	-	-	-	-	-	-	-	-	-	-	-	+	-	NA	NA	hypothetical protein SAB1750c
CAI81441.1	-	-	-	-	-	-	-	-	-	-	-	-	+	-	NA	NA	hypothetical protein SAB1752c
CAI81444.1	-	-	-	-	-	-	-	-	-	-	-	-	+	-	NA	NA	phage anti-repressor protein
CAI81445.1	-	-	-	-	-	-	-	-	-	-	-	-	+	-	NA	NA	hypothetical phage-related protein
CAI81447.1	-	-	-	-	-	-	-	-	-	-	-	-	+	-	NA	NA	hypothetical protein SAB1758
AFR72511.1	+	+	+	-	+	-	-	+	-	+	-	-	+	-	NA	NA	hypothetical protein C248_0498
CAI81448.1	-	-	-	-	-	-	-	-	-	-	-	-	+	-	NA	NA	hypothetical protein SAB1759
CAI81450.1	-	-	-	-	-	-	-	-	-	-	-	-	+	-	NA	NA	hypothetical protein SAB1761c
CAI81452.1	-	-	-	-	-	-	-	-	-	-	-	-	+	-	COG1232	H	protoporphyrinogen oxidase
CAI81453.1	-	-	-	-	-	-	-	-	-	-	-	-	+	-	COG0276	H	ferrochelatase
CAI81454.1	-	-	-	-	-	-	-	-	-	-	-	-	+	-	COG0407	H	uroporphyrinogen decarboxylase
CAI81567.1	-	-	-	-	-	-	-	-	-	-	-	-	+	-	NA	NA	truncated hypothetical protein
CAI81568.1	-	-	-	-	-	-	-	-	-	-	-	-	+	-	COG0697	GER	hypothetical protein SAB1879c
AFR72512.1	+	+	+	-	+	-	-	+	-	+	+	-	-	-	NA	NA	hypothetical protein C248_0499
CAI81570.1	-	-	-	-	-	-	-	-	-	-	-	-	+	-	NA	NA	hypothetical protein SAB1881c
CAI81577.1	-	-	-	-	-	-	-	-	-	-	-	-	+	-	NA	NA	hypothetical protein SAB1888c
CAI81579.1	-	-	-	-	-	-	-	-	-	-	-	-	+	-	NA	NA	hypothetical protein SAB1890

AFR72513.1	+	+	-	-	+	-	-	+	-	-	+	-	+	-	NA	NA	putative membrane protein
CAI81582.1	-	-	-	-	-	-	-	-	-	-	-	-	+	-	NA	NA	hypothetical protein SAB1893c
CAI81589.1	-	-	-	-	-	-	-	-	-	-	-	-	+	-	NA	NA	hypothetical protein SAB1900c
CAI81590.1	-	-	-	-	-	-	-	-	-	-	-	-	+	-	NA	NA	hypothetical protein SAB1901c
Saureus08S 00974_00487	-	+	-	+	-	-	-	-	-	+	-	-	-	-	COG0037	J	tRNA(Ile)-lysidine synthase
AFR72575.1	+	+	-	+	-	-	-	-	+	+	-	-	-	-	COG0634	F	putative hypoxanthine phosphoribosyltransferase
AFR72576.1	+	+	+	+	-	-	+	+	+	+	-	-	-	+	COG0465	O	putative cell division protein
AFR72065.1	+	-	-	-	-	-	-	-	-	-	-	-	-	-	NA	NA	type I restriction-modification system restriction subunit
CAI81734.1	-	-	-	-	-	-	-	-	-	-	-	-	+	-	COG3464	X	transposase
CAI81735.1	-	-	-	-	-	-	-	-	-	-	-	-	+	-	COG3464	X	probable transposase
CAI81740.1	-	-	-	-	-	-	-	-	-	-	-	-	+	-	COG1272	U	probable hemolysin
AFR72577.1	+	+	+	+	-	-	+	+	+	+	-	-	-	+	COG1281	O	putative chaperonin
AFR72578.1	+	+	+	+	-	-	+	+	+	+	-	-	-	+	COG0031	E	Cysteine synthase
CAI82058.1	-	-	-	-	-	-	-	-	-	-	-	-	+	-	NA	NA	hypothetical protein SAB2370c
AFR72579.1	+	+	+	+	-	-	+	+	+	+	-	-	-	+	COG0294	H	dihydropteroate synthase
CAI82064.1	-	-	-	-	-	-	-	-	-	-	-	-	+	-	NA	NA	hypothetical protein SAB2376c
AFR72580.1	+	+	+	+	-	-	+	+	+	+	-	-	-	+	COG1539	H	dihydroneopterin aldolase
CAI82075.1	-	-	-	-	-	-	-	-	-	-	-	-	+	-	NA	NA	hypothetical protein SAB2387
AFR72581.1	+	+	+	+	-	-	+	+	+	+	-	-	-	+	COG0801	H	putative 2-amino-4-hydroxy-6-hydroxymethyldihydropteridine pyrophosphokinase
YP_005732836	-	-	-	-	-	-	-	-	-	-	-	-	-	+	NA	NA	conserved hypothetical protein
YP_00573283	-	-	-	-	-	-	-	-	-	-	-	-	-	+	NA	NA	conserved hypothetical protein
AFR72583.1	+	+	+	+	-	-	+	+	+	+	-	-	-	+	COG1190	J	lysyl-tRNA synthetase
YP_00573283	-	-	-	-	-	-	-	-	-	-	-	-	-	+	NA	NA	conserved hypothetical protein
YP_005732840	-	-	-	-	-	-	-	-	-	-	-	-	-	+	NA	NA	conserved hypothetical protein
YP_005732842	-	-	-	-	-	-	-	-	-	-	-	-	-	+	NA	NA	conserved hypothetical protein
YP_005732844	-	-	-	-	-	-	-	-	-	-	-	-	-	+	NA	NA	conserved hypothetical protein
YP_005732846	-	-	-	-	-	-	-	-	-	-	-	-	-	+	NA	NA	conserved hypothetical protein
YP_005732847	-	-	-	-	-	-	-	-	-	-	-	-	-	+	NA	NA	conserved hypothetical protein
AFR72584.1	+	+	+	+	-	-	+	+	-	+	-	-	-	+	COG1167	KE	GntR family regulatory protein
YP_005732849	-	-	-	-	-	-	-	-	-	-	-	-	-	+	NA	NA	probable HMG-CoA synthase
Saureus08S 00974_00545	-	+	+	+	-	+	-	+	+	+	+	-	-	+	COG0590	J	tRNA-specific adenosine deaminase
YP_005732865	-	-	-	-	-	-	-	-	-	-	-	-	-	+	COG0550	L	DNA topoisomerase III
YP_005732866	-	-	-	-	-	-	-	-	-	-	-	-	-	+	NA	NA	conserved hypothetical protein
YP_005732867	-	-	-	-	-	-	-	-	-	-	-	-	-	+	NA	NA	cassette chromosome recombinase A
YP_005732868	-	-	-	-	-	-	-	-	-	-	-	-	-	+	NA	NA	conserved hypothetical protein
AFR72991.1															COG0561	HR	putative haloacid dehalogenase-like hydrolase
YP_005732869	-	-	-	-	-	-	-	-	-	-	-	-	-	+	COG2217	P	cadmium-translocating P-type ATPase
YP_005732870	-	-	-	-	-	-	-	-	-	-	-	-	-	+	COG0607	P	metallo-beta-lactamase family protein
YP_005732871	-	-	-	-	-	-	-	-	-	-	-	-	-	+	COG0491	R	metallo-beta-lactamase family protein
YP_005732875	-	-	-	-	-	-	-	-	-	-	-	-	-	+	NA	NA	copper P-type ATPase AtkB
YP_005732876	-	-	-	-	-	-	-	-	-	-	-	-	-	+	NA	NA	lipoprotein, putative
Saureus08S 00974_00547	-	+	+	+	-	+	-	+	+	+	+	-	-	+	COG0431	C	FMN-dependent NADPH-azoreductase
AFR72066.1	+	-	-	-	-	-	-	-	-	-	-	-	-	-	COG1518	V	Cas1 family protein
AFR72030.1	+	-	+	-	-	+	-	+	-	-	-	-	+	+	COG3887	T	putative membrane protein
YP_005733149	-	-	-	-	-	-	-	-	-	-	-	-	-	+	COG1396	K	repressor
YP_005733150	-	-	-	-	-	-	-	-	-	-	-	-	-	+	NA	NA	conserved hypothetical protein
YP_005733151	-	-	-	-	-	-	-	-	-	-	-	-	-	+	NA	NA	phage regulatory protein, Rha family
YP_005733153	-	-	-	-	-	-	-	-	-	-	-	-	-	+	NA	NA	conserved hypothetical protein

YP_005733155	-	-	-	-	-	-	-	-	-	-	-	-	-	+	NA	NA	conserved hypothetical protein
Saureus08S 00974_00548	-	+	+	+	-	+	-	-	+	+	-	-	-	+	NA	NA	Serine-aspartate repeat-containing protein C precursor
Saureus08S 00974_00549	-	+	+	+	-	+	-	+	+	+	-	-	-	+	NA	NA	Serine-aspartate repeat-containing protein D precursor
YP_005733169	-	-	-	-	-	-	-	-	-	-	-	-	-	+	NA	NA	conserved hypothetical protein
Saureus08S 00974_00550	-	+	+	+	-	+	-	+	+	+	-	-	-	+	NA	NA	Serine-aspartate repeat-containing protein E precursor
Saureus08S 00974_00551	-	+	+	+	-	+	-	+	+	+	+	-	-	+	COG0438	M	putative poly(glycerol-phosphate) alpha-glucosyltransferase
YP_005733195	-	-	-	-	-	-	-	-	-	-	-	-	-	+	NA	NA	prophage L54a, tail tape meausure protein,
Saureus08S 00974_00552	-	+	-	-	-	-	-	-	-	+	-	-	-	+	COG2826	X	Integrase core domain protein
Saureus08S 00974_00553	-	+	+	+	-	+	-	+	-	+	-	+	-	+	COG0438	M	putative poly(glycerol-phosphate) alpha-glucosyltransferase
Saureus08S 00974_00554	-	+	-	-	-	-	-	-	-	-	+	-	-	+	COG1469	H	GTP cyclohydrolase FolE2
Saureus08S 00974_00555	-	+	-	-	-	-	-	-	-	-	+	-	-	+	COG2120	G	Mycothiol S-conjugate amidase
Saureus08S 00974_00556	-	+	-	-	-	-	-	-	-	-	+	-	-	+	NA	NA	hypothetical protein
YP_005733447	-	-	-	-	-	-	-	-	-	-	-	-	-	+	COG0546	C	indigoidine systhesis protein
Saureus08S 00974_00557	-	+	-	-	-	-	-	-	-	-	-	-	-	+	COG0363	G	Glucosamine-6-phosphate deaminase
AFR72067.1	+	-	-	-	-	-	-	-	-	-	-	-	-	-	NA	NA	CRISPR-associated protein Cas2
YP_005733664	-	-	-	-	-	-	-	-	-	-	-	-	-	+	NA	NA	clumping factor A
YP_005733665	-	-	-	-	-	-	-	-	-	-	-	-	-	+	NA	NA	hypothetical protein SAPIG0865
YP_005733666	-	-	-	-	-	-	-	-	-	-	-	-	-	+	NA	NA	lpxtg-motif cell wall anchor domain
Saureus08S 00974_00558	-	+	-	-	-	-	-	-	-	-	-	-	-	+	COG0269	G	3-hexulose-6-phosphate synthase
Saureus08S 00974_00559	-	+	-	-	-	-	-	-	-	-	-	-	-	+	COG0794	GM	3-hexulose-6-phosphate isomerase
YP_005733749	-	-	-	-	-	-	-	-	-	-	-	-	-	+	COG1074	L	recombination helicase AddA
AFR72867.1	+	+	+	+	-	-	-	-	-	+	-	-	+	+	NA	NA	hypothetical protein C248_0873
AFR72868.1	+	+	+	+	-	-	+	-	+	+	-	-	+	+	NA	NA	putative lipoprotein
YP_005733767	-	-	-	-	-	-	-	-	-	-	-	-	-	+	NA	NA	conjugative transposon protein
YP_005733949	-	-	-	-	-	-	-	-	-	-	-	-	-	+	NA	NA	hypothetical protein SAPIG1152
AFR72871.1	+	+	+	+	-	-	+	-	+	+	-	-	+	+	NA	NA	putative membrane protein
AFR72872.1															COG3153	R	putative acetyltransferase
AFR72873.1	+	+	+	+	-	-	-	-	-	+	-	-	-	-	NA	NA	clumping factor
YP_005734281	-	-	-	-	-	-	-	-	-	-	-	-	-	+	NA	NA	conserved hypothetical protein
AFR72475.1	+	+	+	+	-	+	+	-	+	+	-	-	+	+	NA	NA	secreted von Willebrand factor-binding protein precursor
YP_005734284	-	-	-	-	-	-	-	-	-	-	-	-	-	+	NA	NA	conserved hypothetical protein
YP_005734285	-	-	-	-	-	-	-	-	-	-	-	-	-	+	NA	NA	hypothetical protein SAPIG1489
YP_005734286	-	-	-	-	-	-	-	-	-	-	-	-	-	+	NA	NA	conserved hypothetical protein
AFR72876.1	+	+	+	+	-	-	+	-	+	+	-	-	+	+	NA	NA	putative exported protein
YP_005734292	-	-	-	-	-	-	-	-	-	-	-	-	-	+	NA	NA	conserved hypothetical protein
YP_005734293	-	-	-	-	-	-	-	-	-	-	-	-	-	+	NA	NA	hypothetical phage-related protein
AFR72877.1	+	+	+	+	-	-	+	-	+	+	-	-	+	+	NA	NA	hypothetical protein C248_0883
AFR72068.1	+	-	-	-	-	-	-	-	-	-	-	-	-	-	COG1353	V	CRISPR-associated Csm1 family protein
AFR72878.1	+	+	+	+	-	-	+	-	+	+	-	-	+	+	COG1525	L	thermonuclease precursor
AFR72880.1	-	-	-	-	-	-	-	-	-	-	-	-	-	+	COG1278	K	putative cold shock protein

AFR72881.1	+	+	+	+	-	-	+	-	+	+	-	-	-	-	NA	NA	hypothetical protein C248_0887
AFR72882.1	+	+	+	+	-	-	+	-	+	+	-	-	-	+	NA	NA	hypothetical protein C248_0888
YP_005734599	-	-	-	-	-	-	-	-	-	-	-	-	-	+	COG0115	EH	D-amino acid aminotransferase
AFR72883.1	+	+	+	+	-	-	+	-	+	+	-	-	-	+	NA	NA	putative exported protein
AFR72884.1	+	+	+	+	-	-	+	-	+	+	-	-	-	+	NA	NA	Hypothetical protein C248_0890
Saureus08S 00974_00791	-	+	+	+	-	-	+	-	+	+	-	-	-	-	NA	NA	hypothetical protein
YP_005734680	-	-	-	-	-	-	-	-	-	-	-	-	-	+	NA	NA	conserved hypothetical protein
YP_005734789	-	-	-	-	-	-	-	-	-	-	-	-	-	+	NA	NA	conserved hypothetical protein
YP_005734790	-	-	-	-	-	-	-	-	-	-	-	-	-	+	COG2826	X	transposase InsI for insertion sequence element
AFR72885.1	+	+	+	+	-	-	+	-	+	+	-	-	-	+	NA	NA	hypothetical protein C248_0891
YP_005734791	-	-	-	-	-	-	-	-	-	-	-	-	-	+	NA	NA	N-region of transposase of
YP_005734793	-	-	-	-	-	-	-	-	-	-	-	-	-	+	NA	NA	conserved hypothetical protein
YP_005734794	-	-	-	-	-	-	-	-	-	-	-	-	-	+	COG3942	M	transfer complex protein TraG
YP_005734795	-	-	-	-	-	-	-	-	-	-	-	-	-	+	NA	NA	membrane protein, putative
YP_005734796	-	-	-	-	-	-	-	-	-	-	-	-	-	+	NA	NA	ftsk/spoiiie family protein
YP_005734797	-	-	-	-	-	-	-	-	-	-	-	-	-	+	NA	NA	ftsk/spoiiie family protein
YP_005734798	-	-	-	-	-	-	-	-	-	-	-	-	-	+	NA	NA	conserved hypothetical protein
YP_005734799	-	-	-	-	-	-	-	-	-	-	-	-	-	+	NA	NA	conserved hypothetical protein
YP_005734800	-	-	-	-	-	-	-	-	-	-	-	-	-	+	NA	NA	conserved hypothetical protein
AFR72886.1	+	+	+	+	-	-	+	-	+	+	-	-	-	+	NA	NA	Hypothetical protein C248_0892
YP_005734801	-	-	-	-	-	-	-	-	-	-	-	-	-	+	NA	NA	conserved hypothetical protein
YP_005734802	-	-	-	-	-	-	-	-	-	-	-	-	-	+	NA	NA	replication initiation factor family protein
YP_005734803	-	-	-	-	-	-	-	-	-	-	-	-	-	+	NA	NA	replication initiation factor family protein
YP_005734804	-	-	-	-	-	-	-	-	-	-	-	-	-	+	NA	NA	conserved hypothetical protein
YP_005734805	-	-	-	-	-	-	-	-	-	-	-	-	-	+	NA	NA	conserved hypothetical protein
YP_005734806	-	-	-	-	-	-	-	-	-	-	-	-	-	+	NA	NA	conserved hypothetical protein
YP_005734807	-	-	-	-	-	-	-	-	-	-	-	-	-	+	NA	NA	conserved hypothetical protein
Saureus08S 00974_00794	-	+	+	+	-	-	+	-	+	+	-	-	-	+	COG0406	G	Putative phosphoserine phosphatase 2
AFR72069.1	+	-	-	-	-	-	-	-	-	-	-	-	-	-	NA	NA	CRISPR-associated Csm2 protein
AFR72888.1	+	+	+	+	-	-	+	-	+	+	-	-	-	+	COG1279	E	putative LysE type translocator protein
YP_005734970	-	-	-	-	-	-	-	-	-	-	-	-	-	+	NA	NA	transcriptional antiterminator, BglG family/DNA-binding protein
AFR72889.1	+	+	+	+	-	-	+	-	+	+	-	-	-	+	COG1670	JO	hypothetical protein C248_0895
AFR72890.1	+	+	+	+	-	-	+	-	+	+	-	-	-	+	COG1764	V	OsmC-like protein
AFR72891.1	+	+	+	+	-	-	+	-	+	+	-	-	-	+	COG0710	E	putative type I 3-dehydroquinase
AFR72966.1	+	+	-	+	-	-	-	-	-	+	-	-	-	+	NA	NA	Putative transposon integrase
AFR72968.1	+	+	-	+	-	-	-	-	-	+	-	-	-	+	NA	NA	Hypothetical protein C248_0974
AFR72970.1	+	+	-	+	-	-	-	-	-	+	-	-	-	-	NA	NA	hypothetical protein C248_0976
AFR72971.1	+	+	-	+	-	-	-	-	-	+	-	-	-	+	NA	NA	hypothetical protein C248_0977
AFR72972.1	+	+	-	+	-	-	-	-	-	+	-	-	-	+	COG1396	K	transcriptional regulator, putative
AFR72973.1	+	+	-	+	-	-	-	-	-	+	-	-	-	+	COG0480	J	tetracycline resistance protein
AFR72070.1	+	-	-	-	-	-	-	-	-	-	-	-	-	-	NA	NA	CRISPR-associated RAMP protein, Csm3 family
AFR72974.1	+	+	-	+	-	-	-	-	-	+	-	-	-	+	NA	NA	Hypothetical protein C248_0980
AFR72975.1	+	+	-	+	-	-	-	-	-	+	-	-	-	+	COG0791	M	hypothetical protein C248_0981
AFR72976.1	+	+	-	+	-	-	-	-	-	+	-	-	-	+	NA	NA	Hypothetical protein C248_0982
AFR72977.1	+	+	-	+	-	-	-	-	-	+	-	-	-	+	COG0433	L	hypothetical protein C248_0983
AFR72979.1	+	+	-	+	-	-	-	-	-	+	-	-	-	+	NA	NA	Antirestriction protein
AFR72980.1	+	+	-	+	-	-	-	-	-	+	-	-	-	+	NA	NA	hypothetical protein C248_0986
AFR72981.1	+	+	-	+	-	-	-	-	-	+	-	-	-	+	NA	NA	Transcriptional regulator
AFR72983.1	+	+	-	+	-	-	-	-	-	+	-	-	-	+	COG1674	D	hypothetical protein C248_0989
AFR72984.1	+	+	-	+	-	-	-	-	-	+	-	-	-	+	NA	NA	hypothetical protein C248_0990

AFR72985.1	+	+	-	+	-	-	-	-	-	+	-	-	-	-	NA	NA	hypothetical protein C248_0991
AFR72071.1	+	-	-	-	-	-	-	-	-	-	-	-	-	-	NA	NA	CRISPR-associated RAMP protein
AFR72987.1	+	+	-	+	-	-	-	-	-	+	-	-	-	+	COG3666	X	putative transposase
AFR72989.1	+	-	-	-	-	-	-	-	-	+	-	-	-	+	NA	NA	Putative transposase
AFR72990.1	+	+	-	+	-	-	-	-	-	+	-	-	-	+	COG0446	I	coenzyme A disulfide reductase
AFR72992.1	+	+	-	+	-	-	-	-	-	+	-	-	-	-	COG2151	O	hypothetical protein C248_0998
AFR72994.1	+	+	-	+	-	-	-	-	-	+	-	-	-	-	NA	NA	putative membrane protein
AFR72995.1	+	+	-	+	-	-	-	-	-	+	-	-	-	-	COG0542	O	putative ATPase subunit of an ATP-dependent
AFR73458.1	+	+	+	+	+	+	+	+	+	-	+	+	+	+	NA	NA	hypothetical protein C248_1468
AFR73459.1	+	+	+	+	+	+	+	+	+	-	+	+	+	+	COG1413	R	Conserved virulence factor C
AFR73460.1	+	+	+	+	+	+	+	+	+	-	+	+	+	+	NA	NA	hypothetical protein C248_1470
AFR72072.1	+	-	-	-	-	-	-	-	-	-	-	-	-	-	NA	NA	CRISPR-associated RAMP protein, Csm5 family
AFR73462.1	+	+	+	+	+	+	+	+	+	-	+	+	+	+	COG1738	S	putative membrane protein
AFR73463.1	+	+	+	+	+	+	+	+	+	-	+	+	+	+	COG0328	L	hypothetical protein C248_1473
AFR73464.1	+	+	+	+	-	-	-	-	+	-	-	-	-	+	NA	NA	Extracellular matrix-binding protein ebhB
Saureus08S 00974_01342	-	+	+	+	+	+	+	+	+	+	-	+	+	+	NA	NA	Quinolone resistance protein NorB
Saureus08S 00974_01343	-	+	+	+	+	+	+	+	+	+	-	+	+	+	COG0531	E	Serine/threonine exchanger SteT
Saureus08S 00974_01344	-	+	+	+	+	+	+	+	+	+	-	+	+	+	COG1171	E	L-threonine dehydratase catabolic TdcB
Saureus08S 00974_01345	-	+	+	-	+	+	+	+	+	-	+	-	-	+	COG0686	E	Alanine dehydrogenase 1
Saureus08S 00974_01346	-	+	-	-	-	-	-	+	-	-	-	-	-	-	COG0258	L	5'-3' exonuclease
Saureus08S 00974_01397	-	+	-	+	-	+	+	+	-	+	-	-	+	+	NA	NA	Bacteriophage holin
AFR72382.1	+	+	+	+	+	+	+	+	+	+	+	+	+	+	NA	NA	putative phage protein
AFR72073.1	+	-	-	-	-	-	-	-	-	-	-	-	-	-	NA	NA	CRISPR-associated protein, Csm6 family
AFR72381.1	+	+	-	+	+	+	+	+	-	+	-	-	-	+	NA	NA	putative phage protein
AFR72380.1	+	+	-	+	+	+	+	+	+	+	-	-	+	+	NA	NA	putative phage protein
AFR72379.1	+	+	-	+	+	+	+	-	-	+	-	+	-	+	NA	NA	putative phage protein
AFR72378.1	+	+	-	+	+	+	+	+	+	+	+	-	+	+	NA	NA	putative phage protein
AFR72377.1	+	+	-	-	+	+	+	-	-	+	-	-	-	+	NA	NA	putative phage protein
AFR72376.1	+	+	-	-	+	+	+	-	+	+	+	+	-	+	NA	NA	putative phage protein
AFR72375.1	+	+	-	-	+	+	+	-	-	+	-	-	-	+	NA	NA	putative phage protein
AFR72374.1	+	+	-	-	+	-	+	-	-	+	+	+	-	+	NA	NA	putative phage protein
AFR72373.1	+	+	-	-	+	+	+	-	-	+	+	+	-	+	NA	NA	hypothetical protein C248_0360
AFR72372.1	+	+	-	-	+	+	+	-	-	+	+	+	-	+	NA	NA	hypothetical protein C248_0359
AFR72074.1	+	-	-	-	-	-	-	-	-	-	-	-	-	-	NA	NA	CRISPR-associated protein C
AFR72371.1	+	+	-	-	+	+	-	+	+	+	+	+	-	+	COG5492	R	major tail protein
AFR73789.1	+	+	+	-	-	+	+	-	+	-	-	+	-	+	NA	NA	putative exported protein
AFR73790.1	+	+	+	+	+	+	+	+	+	-	+	+	+	+	COG1187	J	RNA pseudouridine synthase
AFR73791.1	+	+	+	+	+	+	+	+	+	+	+	+	+	+	COG2244	M	Polysaccharide biosynthesis protein
AFR73792.1	+	+	+	+	+	+	+	+	+	+	+	+	+	+	COG2081	R	putative exported protein
AFR73793.1	+	+	+	+	-	-	-	+	+	+	+	-	-	+	NA	NA	putative surface anchored protein
AFR73794.1	+	+	+	+	+	+	+	+	+	+	+	+	+	+	COG0607	P	hypothetical protein C248_1804
AFR73795.1	+	+	+	+	+	+	+	+	+	+	+	+	+	+	COG0495	J	leucyl-tRNA synthetase
AFR73796.1	+	+	+	+	+	+	+	+	+	+	+	+	+	+	COG2814	G	putative membrane protein
AFR73797.1	+	+	+	+	+	+	+	+	+	+	+	+	+	+	COG1242	R	hypothetical protein C248_1807
AFR72075.1	+	-	+	+	-	-	-	-	-	-	-	-	-	-	NA	NA	hypothetical protein C248_0061
AFR73798.1	+	+	+	+	+	+	+	+	+	+	-	-	+	+	COG0500	QR	hypothetical protein C248_1808

AFR73799.1	+	+	+	+	+	+	+	+	+	+	+	+	+	+	COG1846	K	Toxin repressor
AFR73800.1	+	+	+	+	+	+	+	+	-	-	+	+	+	+	COG2267	I	hypothetical protein C248_1810
AFR73802.1	+	+	+	-	-	+	-	-	-	-	+	+	+	+	COG0506	E	proline dehydrogenase
Saureus08S 00974_01807	+	-	-	+	-	-	-	-	-	+	-	+	-	-	COG0219	J	tRNA (cytidine(34)-2'-O)-methyltransferase
Saureus08S 00974_01808	-	+	-	+	-	-	+	-	-	+	-	+	-	-	COG1600	J	Epoxyqueuosine reductase
Saureus08S 00974_01809	-	+	-	+	-	-	+	-	+	+	+	+	+	+	COG1126	E	L-cystine import ATP-binding protein TcyC
Saureus08S 00974_01810	-	+	-	+	-	-	+	-	-	+	-	+	-	-	COG0834	ET	Arginine transport system permease protein ArtQ
Saureus08S 00974_01811	-	+	-	+	-	-	+	-	-	+	-	+	-	-	COG3641	S	Phosphotransferase system, EIIC
Saureus08S 00974_01841	-	+	-	+	-	-	+	-	+	+	-	+	-	-	COG0735	P	Peroxide-responsive repressor PerR
AFR72076.1	+	-	-	-	-	-	-	-	-	-	-	-	-	-	NA	NA	Putative transposase
AFR72031.1	+	-	+	-	-	+	-	+	-	-	-	-	+	+	COG0359	J	50S ribosomal protein L9
Saureus08S00974_01	-	+	-	+	-	-	+	-	+	+	-	+	-	-	COG0111	HR	Glyoxylate/hydroxypyruvate reductase B
Saureus08S 00974_01843	-	+	-	+	-	-	+	-	+	+	-	+	-	-	COG1225	O	Putative peroxiredoxin bcp
Saureus08S 00974_01844	-	+	-	+	-	-	+	-	+	+	-	+	-	-	COG0001	H	Glutamate-1-semialdehyde 2,1-aminomutase 2
Saureus08S 00974_01845	-	+	-	+	-	-	+	-	+	+	-	+	-	-	COG4129	S	Fusaric acid resistance protein family protein
Saureus08S 00974_01846	-	+	-	+	-	-	+	-	+	+	-	+	-	-	COG1132	V	Putative multidrug export ATP-binding/permease protein
Saureus08S 00974_01847	-	+	-	+	-	-	+	-	+	+	-	+	-	-	COG3557	S	hypothetical protein
Saureus08S 00974_01848	-	+	-	+	-	-	+	-	+	+	-	+	-	-	COG1194	L	putative A/G-specific adenine glycosylase YfhQ
Saureus08S 00974_01849	-	+	-	+	-	-	+	-	+	+	-	+	-	-	COG1988	R	hypothetical protein
Saureus08S 00974_02069	-	+	-	-	-	+	-	-	-	-	-	-	-	-	COG3666	X	Transposase DDE domain protein
Saureus08S 00974_02070	-	+	-	-	-	-	-	-	-	+	-	-	-	-	COG0449	M	Glutamine--fructose-6-phosphate aminotransferase
AFR72077.1	+	-	-	-	-	-	-	-	-	-	-	-	-	-	COG4584	X	Hypothetical protein C248_0063
Saureus08S 00974_02071	-	+	+	-	-	-	+	+	-	+	+	-	+	-	COG2213	G	PTS system mannitol-specific EIICB component
AFR74134.1	+	+	+	-	-	-	+	-	-	+	+	+	+	-	COG1762	GT	putative transcriptional antiterminator
AFR74135.1	+	+	+	+	-	-	+	+	-	+	+	+	+	+	COG4668	G	PTS system, mannitol-specific IIA component
AFR74136.1	+	+	+	+	-	-	+	+	-	+	+	+	+	+	COG0246	G	putative mannitol-1-phosphate 5-dehydrogenase
AFR74137.1	+	+	+	+	+	+	+	+	-	+	+	+	+	+	NA	NA	fmtB protein
AFR74138.1	+	+	+	+	-	-	+	+	-	+	+	+	+	+	COG1109	G	putative phosphoglucosamine mutase
AFR74139.1	+	+	+	+	-	-	+	+	-	+	+	+	+	+	COG4856	S	putative exported protein
AFR74140.1	+	+	+	+	-	-	+	+	-	+	+	+	+	+	COG1624	T	putative membrane protein
AFR74141.1	+	+	+	+	-	-	+	+	-	+	+	+	+	+	COG0010	E	arginase
AFR74142.1	+	+	+	+	-	-	+	+	-	+	+	+	+	+	NA	NA	hypothetical protein C248_2202
AFR72078.1	+	-	-	-	-	-	-	-	-	-	-	-	-	-	NA	NA	putative transposase
AFR74143.1	+	+	+	-	-	-	-	-	-	+	-	-	+	+	COG2814	G	Putative multidrug resistance transporter
Saureus08S 00974_02088	-	+	-	-	-	-	-	-	-	-	-	-	+	-	NA	NA	Multidrug resistance efflux pump SepA
Saureus08S [illegible]	-	+	-	-	-	-	-	-	-	-	-	-	+	-	COG0477	GEPR	Multidrug resistance protein 3

00974_02089	-	+	-	-	-	-	-	-	-	-	-	-	+	-	COG0477	GEPR	Multidrug resistance protein 3
AFR74377.1	+	+	-	-	+	+	+	-	-	+	-	-	+	+	COG0789	K	HTH-type transcriptional activator tipA
AFR74378.1	+	+	-	-	+	+	+	+	+	+	+	+	+	+	COG2197	TK	putative response regulator
AFR74379.1	+	+	-	-	+	+	+	+	-	+	+	+	+	+	COG4585	T	putative histidine kinase
AFR74380.1	+	+	-	-	+	-	+	+	-	+	+	+	+	+	COG2203	T	hypothetical protein C248_2440
AFR74381.1	+	+	-	-	+	+	+	+	-	+	+	+	+	+	COG2181	CP	putative nitrate reductase gamma chain
AFR74382.1	+	+	-	-	+	+	+	+	-	+	+	+	+	+	COG2180	CPO	respiratory nitrate reductase delta chain
AFR74383.1	+	+	-	-	+	+	+	+	-	+	+	+	+	+	COG1140	CP	nitrate reductase beta chain
AFR72079.1	+	-	-	+	-	-	-	-	-	-	-	-	-	+	COG1484	L	IstB-like ATP binding protein
AFR74384.1	+	+	-	-	+	+	+	+	-	+	+	+	+	+	COG5013	CP	nitrate reductase alpha chain
AFR74385.1	+	+	-	-	+	+	+	+	-	+	+	+	+	+	COG0007	H	Tetrapyrrole methyltransferase
AFR74386.1	+	+	-	-	+	+	+	+	-	+	+	+	+	+	COG2146	PQ	assimilatory nitrite reductase small subunit
AFR74387.1	+	+	-	-	+	+	+	+	-	-	+	+	+	+	COG1251	C	nitrite reductase large subunit
AFR74388.1	+	+	-	-	+	+	+	+	-	+	+	+	+	+	COG2138	H	Regulatory protein
AFR74389.1	+	+	-	-	+	+	+	+	-	+	+	+	+	+	NA	NA	acetyltransferase GNAT family protein
AFR74390.1	+	+	-	-	+	+	+	+	-	+	+	+	+	+	COG2116	P	putative nitrite transporter
AFR74391.1	+	+	-	-	+	+	+	+	-	+	+	+	+	+	NA	NA	putative membrane protein
AFR74392.1	+	+	-	-	+	+	+	+	-	-	+	+	+	+	COG3832	S	hypothetical protein C248_2452
AFR72080.1	+	-	-	+	-	-	-	-	-	-	-	-	-	-	NA	NA	hypothetical protein C248_0066
AFH68375.1	-	-	+	-	-	-	-	-	-	-	-	-	-	-	NA	NA	hypothetical protein ST398NM01_2800
AFH68381.1	-	-	+	-	-	-	-	-	-	-	-	-	-	-	NA	NA	hypothetical protein ST398NM01_2801
AFR72081.1	+	-	-	-	-	-	-	-	-	-	-	-	-	-	NA	NA	Transposase
AFH68713.1	-	-	+	-	-	-	-	-	-	-	-	-	-	-	NA	NA	Hypothetical protein ST398NM01_2819
AFR72336.1	+	-	-	-	-	-	-	-	-	-	-	-	-	+	NA	NA	putative phage protein
AFH68721.1	-	-	+	-	-	-	-	-	-	-	-	-	-	-	NA	NA	hypothetical protein ST398NM01_2821
AFR72337.1	+	-	-	-	-	-	-	-	-	-	-	-	-	-	NA	NA	hypothetical protein C248_0324
AFR72338.1	+	-	-	-	+	+	-	+	-	-	-	+	-	+	NA	NA	putative DNA-binding protein
AFR72339.1	+	-	+	-	-	+	+	-	+	+	+	+	+	-	NA	NA	putative phage protein
AFH68744.1	-	-	+	-	-	-	-	-	-	-	-	-	-	-	NA	NA	hypothetical protein ST398NM01_0516
AFH68746.1	-	-	+	-	-	-	-	-	-	-	-	-	-	-	NA	NA	hypothetical protein ST398NM01_2822
AFH68747.1	-	-	+	-	-	-	-	-	-	-	-	-	-	-	NA	NA	hypothetical protein ST398NM01_2823
AFR72340.1	+	-	-	-	-	-	-	-	-	-	-	+	-	+	NA	NA	putative phage protein
AFR72032.1	+	-	+	-	-	+	-	+	-	-	-	-	+	+	COG0305	L	DnaB-like helicase
AFH68763.1	-	-	+	-	-	+	-	-	-	-	+	-	+	-	NA	NA	Acetyltransferase
AFH68764.1	-	-	+	-	-	-	+	-	-	-	+	+	+	-	COG5438	S	putative membrane spanning protein
AFH68765.1	-	-	+	-	-	-	+	-	-	-	+	+	+	-	COG5438	S	putative membrane spanning protein
AFH68766.1	-	-	+	-	-	-	+	-	-	-	+	+	+	-	COG0583	K	GltC
AFH68767.1	-	-	+	-	-	-	+	-	-	-	-	+	+	-	COG0070	E	Glutamate synthase [NADPH] large chain
AFR72341.1	+	-	-	-	-	-	-	-	-	-	-	+	-	+	NA	NA	putative phage protein
AFH68768.1	-	-	+	-	-	-	+	-	-	-	-	+	+	-	COG0493	ER	Glutamate synthase [NADPH] small chain
AFH68769.1	-	-	-	+	-	-	+	-	-	-	-	+	+	-	COG1264	G	PTS system, trehalose-specific IIBC component
AFH68770.1	-	-	-	+	-	-	+	-	-	-	-	+	+	-	COG0366	G	Trehalose-6-phosphate hydrolase
AFH68771.1	-	-	-	+	-	-	+	-	-	-	-	+	+	-	COG2188	K	Trehalose operon transcriptional repressor
AFH68772.1	-	-	-	+	-	-	+	-	-	-	-	+	+	-	COG3153	R	Acetyltransferase
AFH68773.1	-	-	+	+	-	-	+	+	-	-	-	+	+	-	COG2812	L	DNA polymerase III subunit gamma/tau
AFH68774.1	-	-	+	+	-	-	+	+	-	-	-	+	+	-	COG0718	R	putative transcriptional regulatory protein
AFH68775.1	-	-	+	+	-	-	+	+	-	-	-	-	+	-	COG0353	L	RecR
AFH68776.1	-	-	+	+	-	-	+	+	-	-	-	-	-	-	NA	NA	Arginine decarboxylase
AFH68777.1	-	-	+	+	-	-	+	+	-	-	-	-	-	-	COG0125	F	Thymidylate kinase
AFR72342.1	+	-	-	-	-	-	-	-	-	-	-	-	-	+	NA	NA	putative phage protein
AFH68778.1	-	-	+	+	-	-	+	+	-	-	-	-	-	-	COG3870	S	putative cytosolic protein

AFH68779.1	-	-	+	+	-	-	-	+	-	-	-	-	-	-	NA	NA	DNA polymerase III, delta' subunit
AFH68780.1	-	-	+	+	-	-	-	+	-	-	-	-	-	-	COG1774	T	Phosphorelay inhibitor
AFH68781.1	-	-	+	+	-	-	-	-	-	-	-	-	-	-	COG4467	L	Initiation-control protein
AFR72343.1	+	-	-	-	-	-	-	-	-	-	-	+	-	+	NA	NA	putative phage protein
AFH68815.1	-	-	-	+	-	-	-	+	-	+	-	-	-	+	COG0214	H	Pyridoxine biosynthesis protein
AFH68816.1	-	-	-	+	-	-	+	+	-	+	-	-	-	-	COG0311	H	Pyridoxine biosynthesis amidotransferase
AFH68817.1	-	-	+	-	-	-	-	-	-	-	-	-	-	-	NA	NA	hypothetical protein ST398NM01_2825
AFH68818.1	-	-	+	-	-	-	-	+	-	-	-	-	-	-	COG1972	F	hypothetical protein ST398NM01_0596
AFH68819.1	-	-	+	-	-	-	-	-	-	-	-	-	-	-	COG4463	K	CtsR
AFH68820.1	-	-	+	-	-	-	-	-	-	-	-	-	-	-	COG3880	O	ClpC ATPase
AFH68850.1	-	-	+	-	-	-	-	-	+	-	+	-	-	-	COG1069	G	L-ribulokinase
AFH68851.1	-	-	-	+	-	-	+	-	+	+	+	-	-	-	COG0451	M	L-threonine 3-dehydrogenase
AFR72344.1	+	-	-	-	-	-	-	-	-	-	-	-	-	+	NA	NA	putative DNA polymerase
AFH68852.1	-	-	+	-	-	+	-	+	+	-	+	-	-	+	COG0115	EH	Branched-chain amino acid aminotransferase
AFH68853.1	-	-	+	-	-	+	-	+	+	-	+	-	-	+	COG0546	C	Putative phosphatase
AFH68854.1	-	-	+	+	-	+	-	+	+	-	-	-	-	+	COG1428	F	Deoxyadenosine kinase
AFH68855.1	-	-	+	+	-	+	-	+	+	-	+	-	-	+	COG1428	F	Deoxyguanosine kinase
AFR72345.1	+	-	-	-	-	-	-	-	-	-	-	-	-	-	NA	NA	Hypothetical protein C248_0332
AFH69094.1	-	-	+	-	-	-	-	-	-	-	-	-	-	-	NA	NA	hypothetical protein ST398NM01_2845
AFR72346.1	+	-	-	+	+	+	+	+	-	+	+	+	-	-	NA	NA	Hypothetical Protein C248_0333
AFR72347.1	+	-	+	+	+	+	+	-	-	+	-	-	+	-	NA	NA	PVL ORF-50-like family
AFR72348.1	+	-	+	+	-	+	+	+	+	+	+	+	+	+	NA	NA	putative phage protein
AFH69378.1	-	-	+	-	-	-	-	-	-	-	-	-	-	-	NA	NA	hypothetical protein ST398NM01_2861
AFH69379.1	-	-	+	-	-	+	-	+	-	-	-	-	+	-	NA	NA	hypothetical protein ST398NM01_2862
AFR72349.1	+	-	+	+	+	+	+	+	-	+	-	-	+	-	NA	NA	PVL orf 52-like protein
AFR72350.1	+	-	-	-	-	-	-	-	-	-	-	-	-	-	NA	NA	Putative dUTP pyrophosphatase
AFR72033.1	+	-	+	-	-	+	-	+	-	-	-	-	+	+	COG0104	F	putative adenylosuccinate synthetase
AFR72351.1	+	-	-	-	-	-	+	-	-	-	-	-	-	-	NA	NA	concerved hypothetical protein
AFR72352.1	+	-	-	+	-	-	-	-	+	-	-	-	-	-	NA	NA	hypothetical protein C248_0339
AFR72353.1	+	-	+	+	+	-	-	-	-	-	-	-	-	+	NA	NA	putative phage protein
AFR72354.1	+	-	+	+	-	+	+	+	-	+	+	+	+	+	NA	NA	Transcriptional activator RinB
AFH70181.1	-	-	+	-	-	-	-	-	-	-	-	-	-	-	NA	NA	hypothetical protein ST398NM01_1156
AFH70182.1	-	-	+	-	-	-	-	-	-	-	-	-	-	-	NA	NA	hypothetical protein ST398NM01_2911
AFH70183.1	-	-	+	-	-	-	-	-	-	-	-	-	-	-	NA	NA	hypothetical protein ST398NM01_2912
AFH70185.1	-	-	+	-	-	-	-	-	-	-	-	-	-	-	NA	NA	Chemotaxis-inhibiting protein
AFH70188.1	-	-	+	-	-	-	-	-	-	-	-	-	-	-	NA	NA	hypothetical protein ST398NM01_2914
AFR72355.1	+	-	+	-	+	+	-	-	-	+	+	+	-	+	NA	NA	Hypothetical protein C248_0342
AFH70190.1	-	-	+	-	+	+	-	-	-	+	+	-	-	-	NA	NA	hypothetical protein ST398NM01_2915
AFH70191.1	-	-	+	-	+	+	-	-	-	+	-	-	-	-	NA	NA	hypothetical protein ST398NM01_2916
AFH70192.1	-	-	+	-	+	+	-	-	-	+	+	-	-	-	NA	NA	Phage-related protein
AFH70193.1	-	-	+	-	+	+	-	-	-	+	+	-	-	-	NA	NA	Phage-related protein
AFH70194.1	-	-	+	-	+	-	-	-	-	+	+	-	-	-	COG0739	M	hypothetical protein ST398NM01_2919
AFH70195.1	-	-	+	-	-	+	-	-	-	+	-	-	-	-	NA	NA	hypothetical protein ST398NM01_2920
AFH70196.1	-	-	+	-	-	+	-	-	-	+	-	-	-	-	NA	NA	hypothetical protein ST398NM01_2921
AFH70197.1	-	-	+	-	-	+	-	-	-	-	-	-	-	-	NA	NA	Phage protein
AFH70198.1	-	-	+	-	-	+	-	-	-	+	-	-	-	-	NA	NA	prophage pi2 protein 39
AFH70199.1	-	-	+	-	-	+	-	-	-	+	-	-	-	-	NA	NA	hypothetical protein ST398NM01_2924
AFR72356.1	+	-	-	-	-	+	-	-	-	-	-	+	-	+	NA	NA	E family protein
AFH70201.1	-	-	+	-	-	+	-	-	-	+	-	-	-	-	NA	NA	Phage protein
AFH70202.1	-	-	+	-	-	+	-	-	-	+	-	-	-	-	NA	NA	Gp6 protein
AFH70203.1	-	-	+	-	-	+	-	-	-	+	-	-	+	-	NA	NA	Phage protein

AFH70204.1	-	-	+	-	-	+	-	-	-	+	-	-	-	-	NA	NA	Major capsid protein
AFH70205.1	-	-	+	-	-	+	-	-	-	+	-	-	-	-	COG0740	O	ClpP
AFH70206.1	-	-	+	-	-	+	-	-	-	+	-	-	-	-	NA	NA	Portal protein
AFH70208.1	-	-	+	-	-	+	-	-	-	+	-	-	-	-	NA	NA	Phage-related protein
AFH70209.1	-	-	+	-	-	-	-	-	-	-	-	-	-	-	NA	NA	hypothetical protein ST398NM01_2932
AFR72357.1	+	-	-	-	-	+	-	-	-	-	-	+	-	+	NA	NA	hypothetical protein C248_0344
AFH70210.1	-	-	+	-	-	+	-	-	-	+	-	-	-	-	COG1403	V	HNH endonuclease family protein
AFH70211.1	-	-	+	-	+	+	-	-	-	+	-	-	-	-	NA	NA	Phage transcriptional activator
AFH70215.1	-	-	+	+	+	+	+	+	-	+	+	+	+	+	NA	NA	hypothetical protein ST398NM01_0362
AFH70216.1	-	-	+	-	+	+	-	+	-	-	-	-	-	-	NA	NA	Deoxyuridine 5'-triphosphate nucleotidohydrolase
AFH70217.1	-	-	+	-	-	-	-	-	+	-	-	-	-	-	NA	NA	hypothetical protein ST398NM01_2936
AFH70218.1	-	-	+	+	+	+	+	+	-	-	+	+	+	+	NA	NA	Phage protein
AFH70219.1	-	-	+	-	-	-	-	-	-	-	-	-	-	-	NA	NA	Hypothetical protein ST398NM01_2937
AFR72358.1	+	-	-	-	-	+	-	-	-	-	-	+	-	+	COG0553	KL	putative phage protein
AFH70222.1	-	-	+	+	+	+	-	-	-	-	-	-	-	-	NA	NA	Phage protein
AFH70223.1	-	-	+	-	-	-	-	-	-	-	-	-	-	-	NA	NA	hypothetical protein ST398NM01_2940
AFH70224.1	-	-	+	-	-	-	-	-	+	-	-	-	-	-	NA	NA	Phage protein
AFH70225.1	-	-	+	-	+	+	+	+	+	-	+	+	+	-	COG0629	L	Single-strand DNA binding protein
AFH70226.1	-	-	+	-	-	-	-	-	-	-	-	-	-	-	NA	NA	Metal-dependent hydrolase
AFH70227.1	-	-	+	-	-	-	-	-	-	-	-	-	-	-	NA	NA	Rect
AFH70228.1	-	-	+	-	-	-	-	-	-	-	-	-	-	-	NA	NA	Hypothetical protein ST398NM01_2945
AFH70229.1	-	-	+	-	-	-	-	-	+	-	-	-	-	-	NA	NA	hypothetical protein ST398NM01_2946
AFR72359.1	+	-	-	-	-	+	+	+	-	-	+	+	+	+	NA	NA	phage regulatory protein
AFH70230.1	-	-	+	-	+	+	+	+	+	+	+	+	+	-	NA	NA	putative cytosolic protein
AFH70231.1	-	-	+	-	-	+	+	-	-	+	-	-	+	-	NA	NA	putative cytosolic protein
AFH70232.1	-	-	+	-	-	-	-	-	-	-	-	-	-	-	NA	NA	hypothetical protein ST398NM01_2949
AFH70234.1	-	-	+	-	-	-	-	-	-	+	-	-	-	-	COG4707	X	Hypothetical protein ST398NM01_2951
AFH70235.1	-	-	+	-	-	-	-	-	-	+	-	-	-	-	NA	NA	hypothetical protein ST398NM01_2952
AFH70236.1	-	-	+	-	-	+	-	-	-	+	-	+	-	-	NA	NA	Phage protein
AFH70237.1	-	-	+	-	+	+	+	+	-	+	+	+	+	-	COG3645	X	Phage antirepressor protein
AFH70238.1	-	-	+	-	-	-	-	-	-	-	-	-	-	-	NA	NA	Phage protein
AFH70239.1	-	-	+	-	-	-	-	-	-	-	-	-	-	-	NA	NA	hypothetical protein ST398NM01_2956
AFR72360.1	+	-	-	-	+	+	-	-	-	-	+	+	-	+	COG1403	V	HNH endonuclease family protein
AFR72034.1	+	-	-	-	-	-	-	-	-	-	-	-	-	-	NA	NA	Hypothetical Protein C248_0020
AFH70240.1	-	-	+	-	-	-	-	-	-	-	-	+	-	-	NA	NA	Phage transcriptional regulator, Cro/CI family
AFH70241.1	-	-	+	-	+	+	-	-	-	+	-	+	+	-	COG1396	K	Phage transcriptional repressor
AFH70242.1	-	-	+	-	-	-	-	-	-	-	-	-	-	-	NA	NA	DNA polymerase III alpha subunit
AFH70243.1	-	-	+	-	-	-	-	-	-	-	-	-	-	-	NA	NA	hypothetical protein ST398NM01_2959
AFH70244.1	-	-	+	-	-	-	-	-	-	+	+	-	-	-	NA	NA	putative cytosolic protein
AFH70247.1	-	-	+	-	-	-	-	-	+	-	+	-	-	-	NA	NA	Sphingomyelin phosphodiesterase
AFR72361.1	+	-	-	-	+	+	+	-	-	+	+	+	-	+	NA	NA	terminase small subunit
AFH70405.1	-	-	+	-	-	-	-	-	-	-	-	-	-	-	NA	NA	Mannitol-1-phosphate 5-dehydrogenase
AFH70412.1	-	-	+	+	-	-	-	-	-	-	-	-	-	-	NA	NA	hypothetical protein ST398NM01_2970
AFH70413.1	-	-	+	-	-	-	-	-	-	-	-	-	-	-	COG0489	D	ApbC
AFR72362.1	+	-	+	-	+	+	+	-	-	+	-	+	-	+	COG4626	X	terminase large subunit
AFH70762.1	-	-	+	+	-	-	-	-	-	+	-	-	+	-	NA	NA	Type II restriction-modification system methylation subunit
AFH70769.1	-	-	+	+	-	-	-	-	-	+	-	-	+	-	NA	NA	Fibronectin-binding protein
AFR72363.1	+	-	-	-	+	+	+	-	-	+	+	+	-	+	NA	NA	portal protein
APE87178.1	-	-	-	+	-	-	-	-	-	-	-	-	-	+	NA	NA	hypothetical protein AS852_00350
APE87179.1	-	-	-	+	-	-	-	-	-	-	-	-	-	-	NA	NA	hypothetical protein AS852_00355
APE87181.1	-	-	-	+	-	-	-	-	-	-	-	-	-	-	NA	NA	hypothetical protein AS852_00365

AFR72364.1	+	-	-	-	+	+	+	-	-	+	+	+	-	+	COG0740	O	phage protease
APE87185.1	-	-	-	+	-	-	-	-	-	-	-	-	-	-	NA	NA	transposase
APE87186.1	-	-	-	+	-	-	-	-	-	-	-	-	-	-	NA	NA	hypothetical protein AS852_00395
APE87187.1	-	-	-	+	-	-	-	-	-	-	-	-	-	-	NA	NA	hydratase
APE87188.1	-	-	-	+	-	-	-	-	-	-	-	-	-	-	NA	NA	hypothetical protein AS852_00405
AFR72365.1	+	-	-	-	+	+	+	-	-	+	+	+	-	+	NA	NA	putative phage capsid protein
APE87533.1	-	-	-	+	-	-	+	-	-	-	-	-	-	-	NA	NA	hypothetical protein AS852_02250
APE89767.1	-	-	-	+	-	-	-	-	-	-	-	-	-	-	NA	NA	hypothetical protein AS852_02310
AFR72366.1	+	-	-	-	+	+	+	-	-	+	+	+	-	+	NA	NA	putative phage protein
APE87544.1	-	-	-	+	-	-	-	-	-	-	-	-	-	-	COG4123	J	hypothetical protein AS852_02330
APE87545.1	-	-	-	+	-	-	-	-	-	-	-	-	-	-	COG2827	L	hypothetical protein AS852_02335
APE87546.1	-	-	-	+	-	-	-	-	-	-	-	-	-	-	COG0313	J	16S rRNA (cytidine(1402)-2'-O)-methyltransferase
APE87547.1	-	-	-	+	-	-	-	-	-	-	-	-	-	-	NA	NA	hypothetical protein AS852_02345
APE87548.1	-	-	-	+	-	-	-	-	-	-	-	-	-	-	COG0073	J	methionine--tRNA ligase
APE87549.1	-	-	-	+	-	-	-	-	-	-	-	-	-	-	COG0084	N	hydrolase TatD
APE87550.1	-	-	-	+	-	-	-	-	-	-	-	-	-	-	COG1658	J	ribonuclease M5
APE89768.1	-	-	-	+	-	-	-	-	-	-	-	-	-	-	COG0030	J	16S rRNA (adenine(1518)-N(6)/adenine(1519)-N(6))-dimethyltransferase
AFR72367.1	+	-	-	-	+	+	+	-	-	+	+	+	-	+	NA	NA	putative phage protein
APE87551.1	-	-	-	+	-	-	-	-	-	-	-	-	-	-	COG4466	S	hypothetical protein AS852_02370
AFR72368.1	+	-	+	-	+	+	+	-	-	+	+	+	-	+	NA	NA	putative phage protein
AFR72369.1	+	-	-	-	+	+	+	-	-	+	+	+	-	+	NA	NA	putative phage protein
AFR72370.1	+	-	-	-	+	+	+	-	-	+	+	+	-	+	NA	NA	major tail protein
AFR72035.1	+	-	+	-	-	+	-	+	-	-	+	-	+	+	COG0745	TK	response regulator protein
APE87872.1	-	-	-	+	-	-	-	-	+	-	-	-	-	+	COG0778	C	nitroreductase
APE88873.1	-	-	-	+	-	-	-	+	-	-	-	-	+	+	NA	NA	autolysin
APE88875.1	-	-	-	+	-	-	-	+	-	-	-	-	-	-	NA	NA	phage tail protein
APE88876.1	-	-	-	+	-	-	+	-	-	-	-	-	+	-	COG4193	G	cell wall hydrolase
APE88880.1	-	-	-	+	-	-	+	+	+	-	-	-	+	-	NA	NA	hypothetical protein AS852_09410
APE88882.1	-	-	-	+	-	-	+	+	+	-	-	-	+	-	NA	NA	peptidase
APE88883.1	-	-	-	+	-	-	+	+	+	-	-	-	-	-	NA	NA	phage tail protein
APE88884.1	-	-	-	+	-	-	+	+	+	-	-	-	+	-	NA	NA	terminase
APE88885.1	-	-	-	+	-	-	+	-	+	-	-	-	-	-	NA	NA	hypothetical protein AS852_09435
APE88886.1	-	-	-	+	-	-	+	+	+	-	-	-	-	-	NA	NA	hypothetical protein AS852_09440
APE88887.1	-	-	-	+	-	-	+	+	+	-	-	-	-	-	NA	NA	phage tail protein
APE88888.1	-	-	-	+	-	-	+	+	-	-	-	-	-	-	NA	NA	hypothetical protein AS852_09450
APE88889.1	-	-	-	+	-	-	+	+	-	-	-	-	-	-	NA	NA	hypothetical protein AS852_09455
APE88890.1	-	-	-	+	-	-	+	+	-	-	-	-	-	-	NA	NA	phage head-tail adapter protein
APE88891.1	-	-	-	+	-	-	+	+	-	-	-	-	-	-	NA	NA	phage head-tail adapter protein
APE88892.1	-	-	-	+	-	-	+	+	-	-	-	-	+	-	NA	NA	major capsid protein
APE88893.1	-	-	-	+	-	-	+	+	-	-	-	-	-	-	NA	NA	hypothetical protein AS852_09475
APE88894.1	-	-	-	+	-	-	+	-	-	-	-	-	-	-	NA	NA	hypothetical protein AS852_09480
APE88895.1	-	-	-	+	-	-	+	+	-	-	-	-	-	-	NA	NA	phage head morphogenesis protein
APE88896.1	-	-	-	+	-	-	+	+	+	-	-	-	+	-	NA	NA	phage portal protein
APE88897.1	-	-	-	+	-	-	+	+	-	-	-	-	-	-	NA	NA	terminase
APE88898.1	-	-	-	+	-	-	+	+	-	-	-	-	-	-	NA	NA	terminase
APE88899.1	-	-	-	+	+	+	-	-	-	-	+	+	-	-	NA	NA	hypothetical protein AS852_09505
APE88900.1	-	-	-	+	-	-	+	+	-	-	-	-	-	-	NA	NA	hypothetical protein AS852_09510
APE88901.1	-	-	-	+	-	-	-	-	-	-	-	-	-	-	NA	NA	hypothetical protein AS852_09515
APE88906.1	-	-	-	+	+	+	+	-	+	+	+	+	+	+	NA	NA	dUTPase
AFR72036.1	+	-	+	-	-	+	-	+	-	-	+	-	+	+	COG5002	T	sensor kinase protein

Source	Target	Distance	Group
Borrelia burgdorferi	Staphylococcus aureus LA*	7.51763	I
Clostridium botulinum	Staphylococcus aureus LA*	8.32624	I
Borrelia afzelii	Staphylococcus aureus LA*	8.60591	I
Clostridium tetani	Staphylococcus aureus LA*	9.93434	I
Rickettsia africae	Staphylococcus aureus LA*	10.1228	I
Rickettsia akari	Staphylococcus aureus LA*	10.3842	I
Mycoplasma hyorhinis	Staphylococcus aureus LA*	10.6426	I
Anaerococcus prevotii	Staphylococcus aureus LA*	10.9594	I
Finegoldia magna	Staphylococcus aureus LA*	11.0287	I
Rickettsia prowazekii	Staphylococcus aureus LA*	11.0743	I
Rickettsia heilongjiangensis	Staphylococcus aureus LA*	11.3296	I
Rickettsia felis	Staphylococcus aureus LA*	11.3418	I
Rickettsia japonica	Staphylococcus aureus LA*	11.3844	I
Rickettsia philipii	Staphylococcus aureus LA*	11.5516	I
Rickettsia canadensis	Staphylococcus aureus LA*	11.5637	I
Melissococcus plutonius	Staphylococcus aureus LA*	11.5759	I
Rickettsia conorii	Staphylococcus aureus LA*	11.582	I
Rickettsia rickettsii	Staphylococcus aureus LA*	11.5911	I
Clostridium saccharoperbutylacetonicum	Staphylococcus aureus LA*	11.6458	I
Mycoplasma mycoides subsp. mycoides	Staphylococcus aureus LA*	11.6473	I
Brachyspira pilosicoli	Staphylococcus aureus LA*	11.6488	I
Rickettsia typhi	Staphylococcus aureus LA*	11.6701	I
Mycoplasma leachii	Staphylococcus aureus LA*	11.7461	I
Rickettsia australis	Staphylococcus aureus LA*	11.8191	I
Mycoplasma capricolum subsp. capricolum	Staphylococcus aureus LA*	11.8616	I
Rickettsia peacockii	Staphylococcus aureus LA*	11.9022	I
Bacillus cereus	Staphylococcus aureus LA*	11.9376	I
Acinetobacter baumannii	Staphylococcus aureus LA*	12.0547	I
Rickettsia slovaca	Staphylococcus aureus LA*	12.0623	I
Rickettsia bellii	Staphylococcus aureus LA*	12.0957	I
Ureaplasma parvum	Staphylococcus aureus LA*	12.2051	I
Mycoplasma bovis	Staphylococcus aureus LA*	12.2355	II
Rickettsia rhipicephali	Staphylococcus aureus LA*	12.2659	II
Rickettsia massiliae	Staphylococcus aureus LA*	12.2842	II
Brachyspira murdochiie	Staphylococcus aureus LA*	12.2902	II
Mycoplasma hominis	Staphylococcus aureus LA*	12.3043	II
Mycoplasma cynos	Staphylococcus aureus LA*	12.3085	II
Borrelia bissettii	Staphylococcus aureus LA*	12.3814	II
Brachyspira hyodysenteriae	Staphylococcus aureus LA*	12.4027	II
Borrelia garinii	Staphylococcus aureus LA*	12.4554	II
Ureaplasma urealyticum	Staphylococcus aureus LA*	12.4696	II
Arcobacter nitrofigilis	Staphylococcus aureus LA*	12.6246	II
Mycoplasma crocodyli	Staphylococcus aureus LA*	12.6297	II
Brachyspira intermedia	Staphylococcus aureus LA*	12.7857	II

Mycoplasma fermentans	Staphylococcus aureus LA*	12.8003	II
Bacteroides fragilis	Staphylococcus aureus LA*	12.8055	II
Bacillus thuringiensis	Staphylococcus aureus LA*	12.8192	II
Rickettsia parkeri	Staphylococcus aureus LA*	12.8496	II
Brachyspira pilosicoli	Staphylococcus aureus LA*	12.9165	II
Mycoplasma penetrans	Staphylococcus aureus LA*	13.0269	II
Methanosphaera stadtmanae	Staphylococcus aureus LA*	13.0441	II
Arcobacter butzleri	Staphylococcus aureus LA*	13.0563	II
Campylobacter jejuni subsp. jejuni	Staphylococcus aureus LA*	13.0563	II
Mycoplasma conjunctivae	Staphylococcus aureus LA*	13.1723	II
Lysinibacillus sphaericus	Staphylococcus aureus LA*	13.2174	II
Mycoplasma arthritidis	Staphylococcus aureus LA*	13.2554	II
Arcobacter sp.	Staphylococcus aureus LA*	13.2964	II
Rickettsia montanensis	Staphylococcus aureus LA*	13.5771	II
Lactobacillus salivarius	Staphylococcus aureus LA*	13.7372	II
Mycoplasma agalactiae	Staphylococcus aureus LA*	13.8436	II
Orientia tsutsugamushi	Staphylococcus aureus LA*	13.8588	II
Mesoplasma florum	Staphylococcus aureus LA*	13.9014	II
Francisella tularensis	Staphylococcus aureus LA*	13.9652	II
Campylobacter lari	Staphylococcus aureus LA*	14.0412	II
Mycoplasma hyopneumoniae	Staphylococcus aureus LA*	14.2373	II
Bacillus anthracis	Staphylococcus aureus LA*	14.3981	II
Mycoplasma suis	Staphylococcus aureus LA*	14.5805	II
Mycoplasma gallisepticum	Staphylococcus aureus LA*	14.6705	II
Campylobacter hominis	Staphylococcus aureus LA*	14.9501	II
Ehrlichia ruminantium	Staphylococcus aureus LA*	15.0292	II
Leptospira interrogans	Staphylococcus aureus LA*	15.0687	II
Clostridium novyi	Staphylococcus aureus LA*	15.1325	II
Clostridium perfringens	Staphylococcus aureus LA*	15.4517	II
Flavobacterium branchiophilum	Staphylococcus aureus LA*	15.5622	II
Francisella sp.	Staphylococcus aureus LA*	15.5916	II
Methanobrevibacter smithii	Staphylococcus aureus LA*	15.6955	II
Clostridium beijerinckii	Staphylococcus aureus LA*	15.8074	II
Butyrivibrio proteoclasticus	Staphylococcus aureus LA*	15.8196	II
Eubacterium eligens	Staphylococcus aureus LA*	15.853	II
Bacillus weihenstephanensis	Staphylococcus aureus LA*	15.8682	II
Acidianus hospitalis	Staphylococcus aureus LA*	15.8864	II
Clostridium difficile	Staphylococcus aureus LA*	15.9807	II
Acholeplasma laidlawii	Staphylococcus aureus LA*	15.9898	II
Campylobacter fetus subsp. fetus	Staphylococcus aureus LA*	15.9924	II
Fusobacterium nucleatum subsp. nucleatum	Staphylococcus aureus LA*	16.1296	II
Borrelia duttonii	Staphylococcus aureus LA*	16.2056	II
Bacillus megaterium	Staphylococcus aureus LA*	16.3059	II
Francisella philomiragia subsp. philomiragia	Staphylococcus aureus LA*	16.3272	II

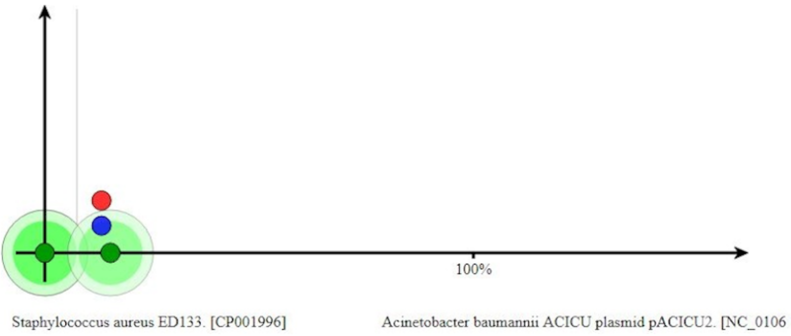
100%
Staphylococcus aureus ED133. [CP001996]
Acinetobacter baumannii ACICU plasmid pACICU2. [NC_0106

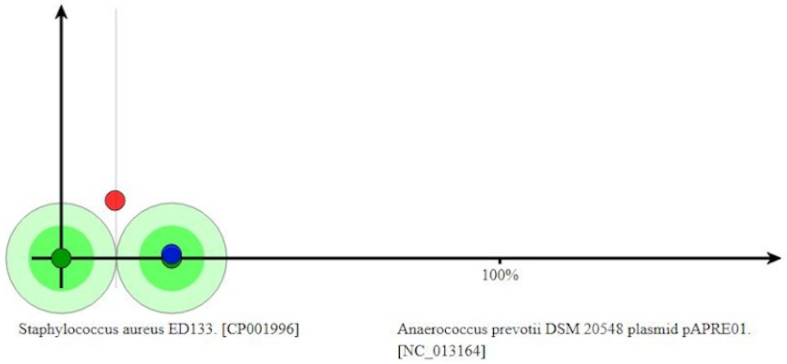
100%
Staphylococcus aureus ED133. [CP001996]
Anaerococcus prevotii DSM 20548 plasmid pAPRE01. [NC_013164]

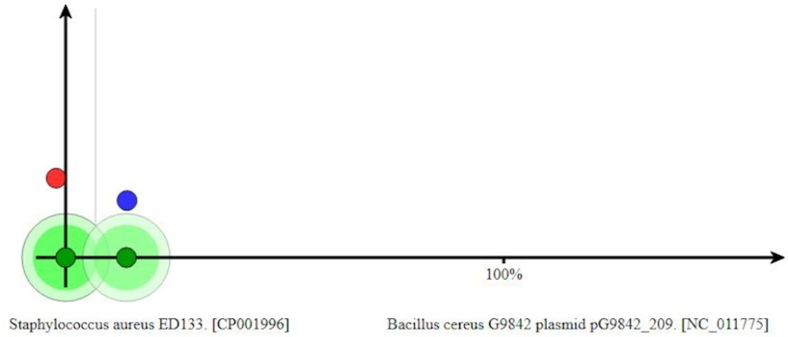
100%
Staphylococcus aureus ED133. [CP001996]
Bacillus cereus G9842 plasmid pG9842_209. [NC_011775]

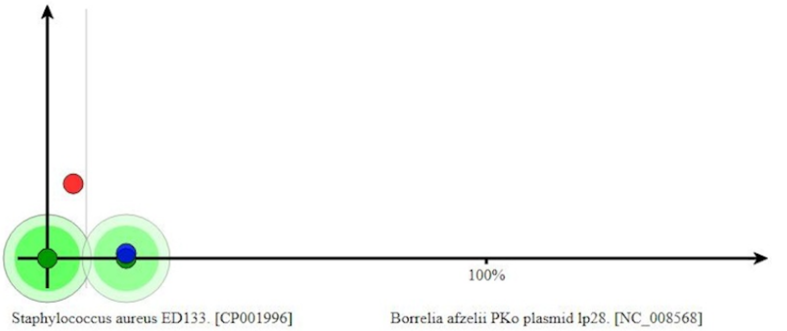
100%
Staphylococcus aureus ED133. [CP001996]
Borrelia afzelii PKo plasmid lp28. [NC_008568]

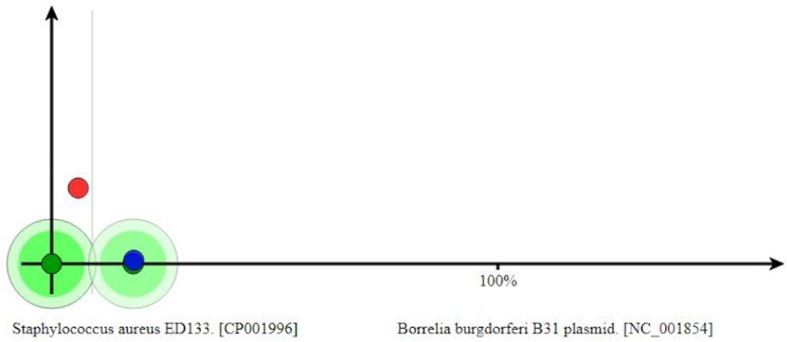
100%
Staphylococcus aureus ED133. [CP001996]
Borrelia burgdorferi B31 plasmid. [NC_001854]

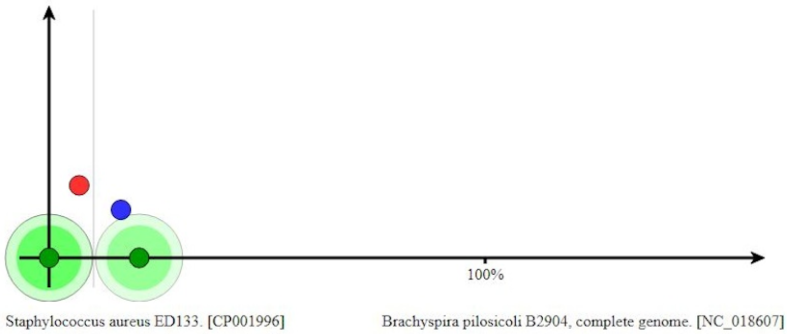
100%
Staphylococcus aureus ED133. [CP001996]
Brachyspira pilosicoli B2904, complete genome. [NC_018607]

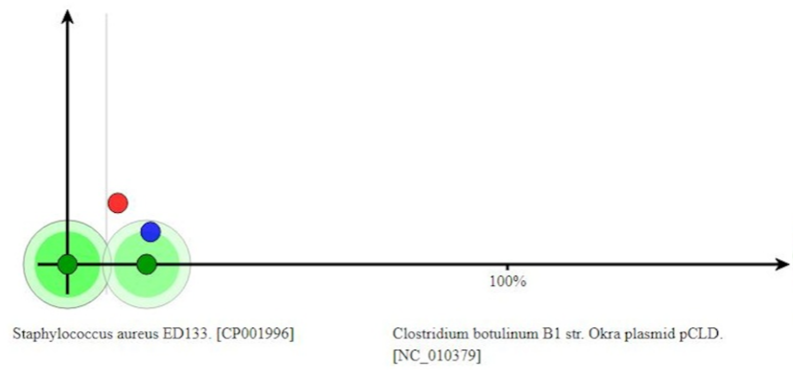
100%
Staphylococcus aureus ED133. [CP001996]
Clostridium botulinum B1 str. Okra plasmid pCLD. [NC_010379]

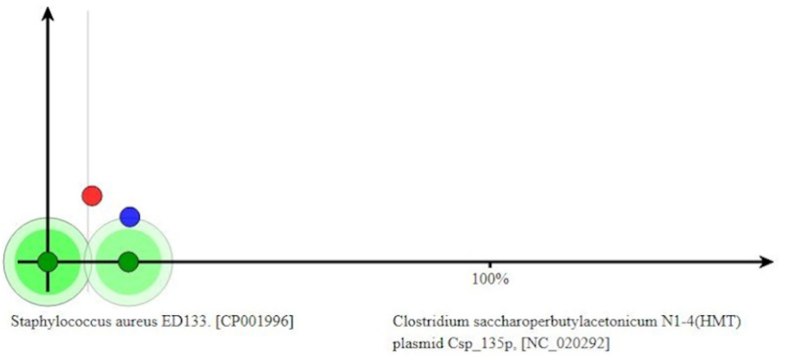
100%
Staphylococcus aureus ED133. [CP001996]
Clostridium saccharoperbutylacetonicum N1-4(HMT) plasmid Csp_135p, [NC_020292]

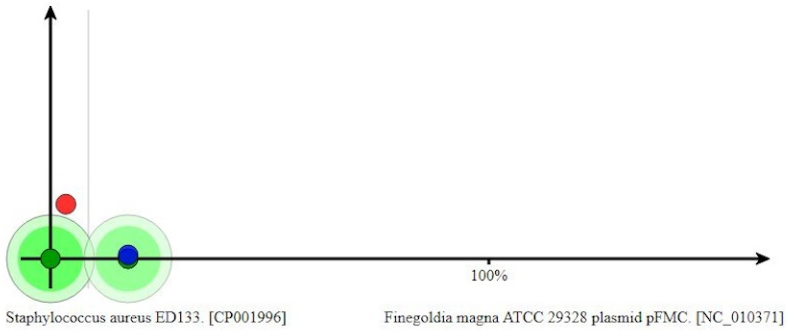
100%
Staphylococcus aureus ED133. [CP001996]
Finegoldia magna ATCC 29328 plasmid pFMC. [NC_010371]

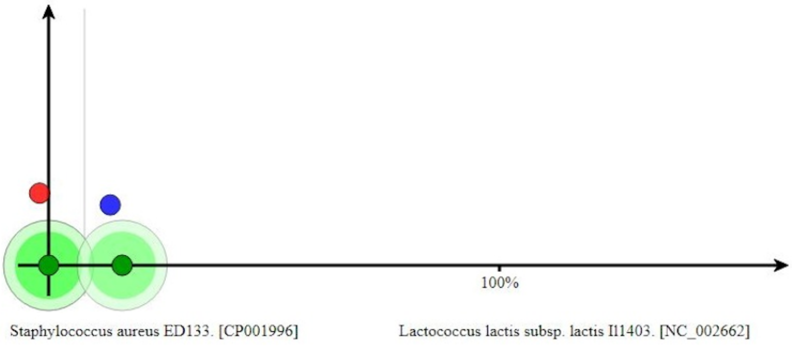
100%
Staphylococcus aureus ED133. [CP001996]
Lactococcus lactis subsp. lactis Il1403. [NC_002662]

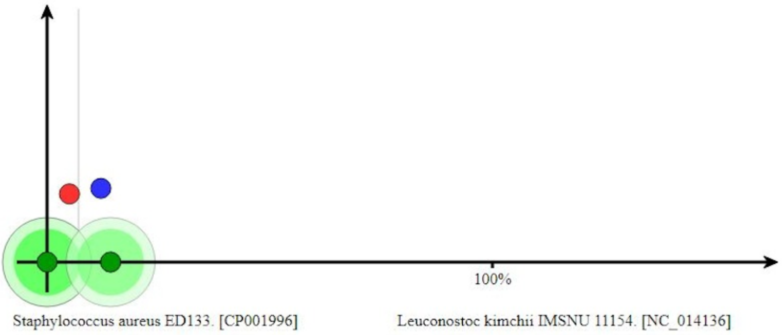
100%
Staphylococcus aureus ED133. [CP001996]
Leuconostoc kimchii IMSNU 11154. [NC_014136]

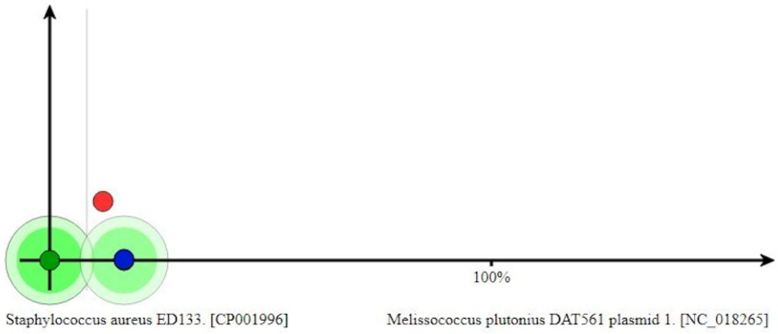
100%
Staphylococcus aureus ED133. [CP001996]
Melissococcus plutonius DAT561 plasmid 1. [NC_018265]

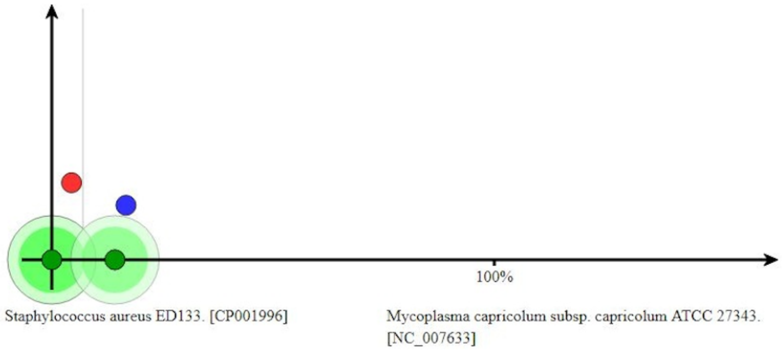
100%
Staphylococcus aureus ED133. [CP001996]
Mycoplasma capricolum subsp. capricolum ATCC 27343. [NC_007633]

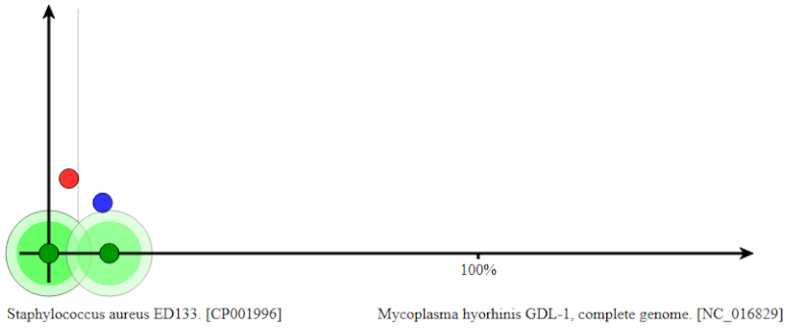
100%
Staphylococcus aureus ED133. [CP001996]
Mycoplasma hyorhinis GDL-1, complete genome. [NC_016829]

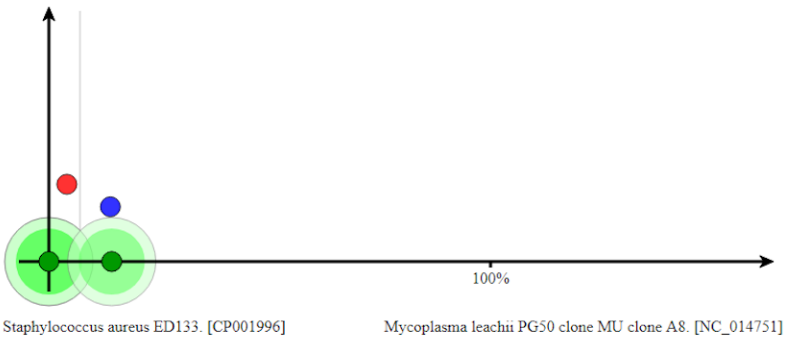
100%
Staphylococcus aureus ED133. [CP001996]
Mycoplasma leachii PG50 clone MU clone A8. [NC_014751]

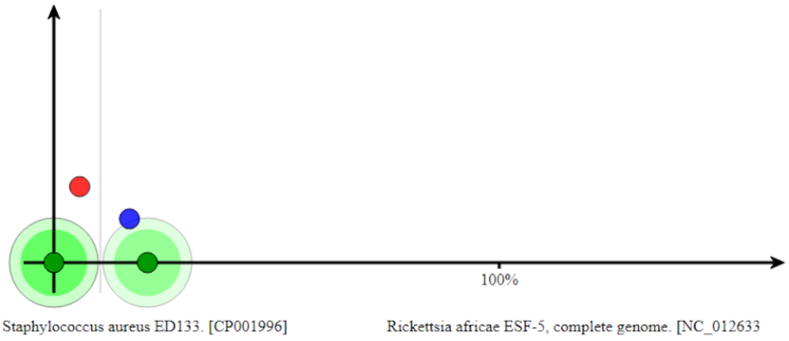
100%
Staphylococcus aureus ED133. [CP001996]
Rickettsia africae ESF-5, complete genome. [NC_012633

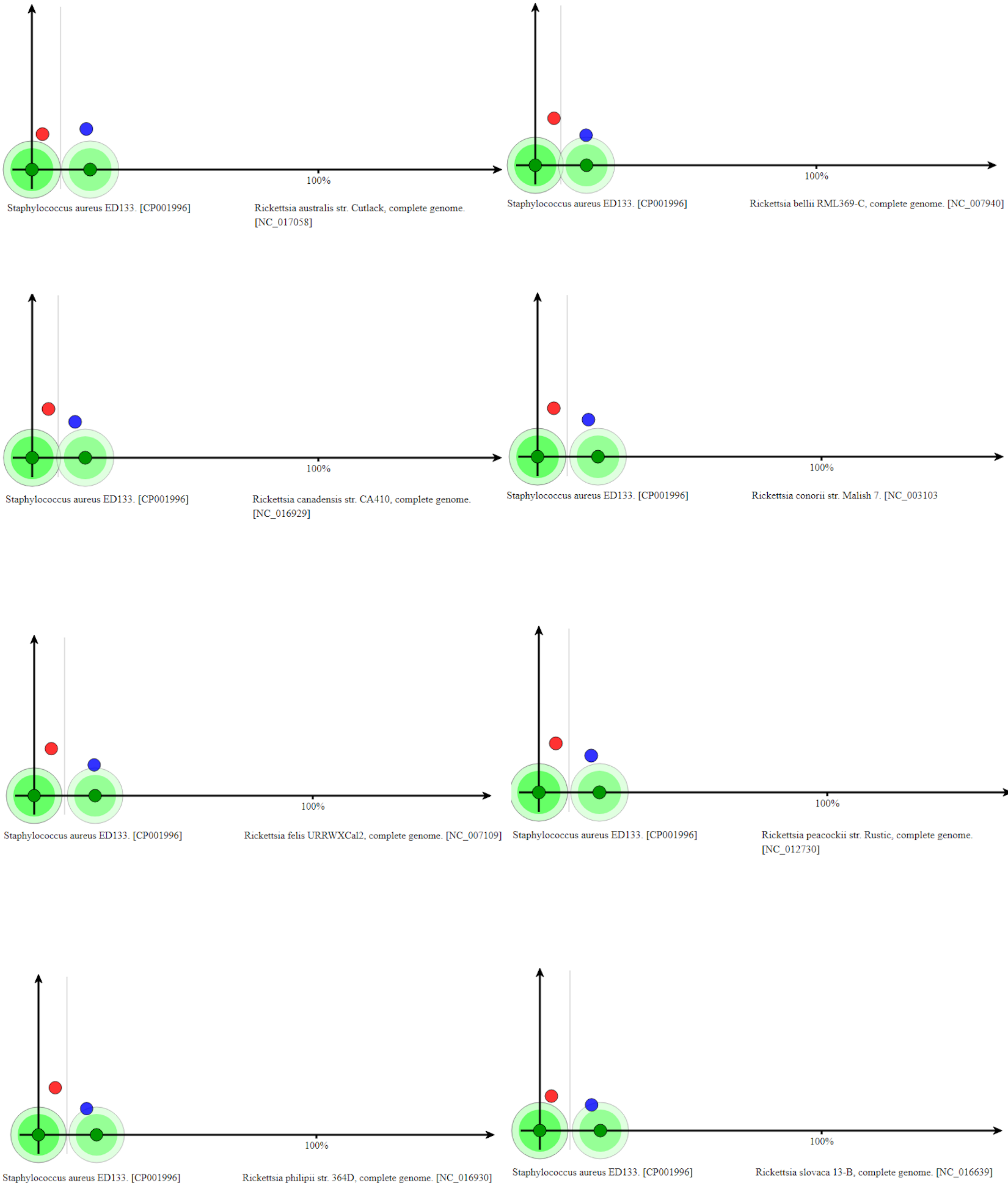

SUPPLEMENTARY DATA. These figures depicts the proposed GI donor-recipient relationship with the aid of Lingvocom 2D projection utility. Two dark green spots on the plots represent OUP of S. aureus ED133 (at centre point) and proposed donors (on principal axis). Light green circle depicts 1/2 of the distance between patterns calculated for chromosomes/plasmids. The island of S. aureus is shown as a red small circle and that of other donors as a blue circle. Islands were plotted along the second principal axis according to distance values between OUP of islands and host chromosome.
